# M2-like GAMs secreting CSTA drive glioblastoma progression via the ITGB4-TGFB1 feedback axis

**DOI:** 10.1186/s12967-026-08009-0

**Published:** 2026-03-14

**Authors:** Jiasheng Wu, Haohao Huang, Xinbo Li, Hongtao Zhu, Yixuan Ma, Xiaojin Liu, Hongbin Liu, Suojun Zhang, Huaqiu Zhang, Ting Lei, Ran Li, Kai Shu, Chao You

**Affiliations:** 1 Department of Neurosurgery, Tongji Hospital, Tongji Medical College, Huazhong University of Science and Technology, Wuhan, China; 2 Department of Neurosurgery, General Hospital of Central Theatre Command of People’s Liberation Army, Wuhan, China; 3 Wuhan University of Science and Technology, Wuhan, China

**Keywords:** CSTA, Glioma associated macrophages/microglia, Glioblastoma, Integrin β4, Molecular docking

## Abstract

**Background:**

Tumor microenvironment (TME) remodeling is a hallmark of gliomas. Glioma-associated macrophage/microglia (GAMs), the most abundant cellular component in the glioma TME, play critical roles in driving glioma progression, though the specific GAM subsets and their regulatory mechanisms remain unclear. This study investigates glioma-GAM crosstalk, identifies GAM subsets that regulate glioblastoma (GBM) progression, clarifies the role and mechanism of CSTA, and explores its clinical relevance.

**Methods:**

With bulk RNA-seq and scRNA-seq datasets, a CSTA⁺ M2-like GAM subset was identified. In vitro and in vivo models confirmed CSTA’s pro-oncogenic effect on GBM. In vitro studies explored the mechanisms of CSTA upregulation in M2-like GAMs and its role in advancing GBM progression. Mass spectrometry and molecular docking predicted and validated CSTA’s interacting protein targets. Clinical cohort analyses evaluated the relationship between CSTA and glioma grades.

**Results:**

M2-like polarization activates p44/p42 (ERK1/2) phosphorylation in the MAPK pathway, upregulating the c-JUN/c-FOS (AP-1) transcription complex and promoting CSTA expression in GAMs. CSTA binds to ITGB4 at glutamate residue 88, activating downstream NF-κB and MAPK signaling in GBM cells to drive progression. Moreover, the CSTA-ITGB4 axis induces GBM cells to secrete TGFB1, which recruits M2-like GAMs and exacerbates TME immunosuppression. Additionally, CSTA levels in peripheral blood and cerebrospinal fluid (CSF) correlate positively with glioma grade.

**Conclusions:**

CSTA⁺ M2-like GAMs promote GBM malignancy via the CSTA-ITGB4 axis, activating downstream NF-κB and MAPK signaling and forming a TGFB1-mediated positive feedback loop. Notably, CSTA correlates with glioma grades and has independent prognostic and potential therapeutic value.

**Graphical Abstract:**

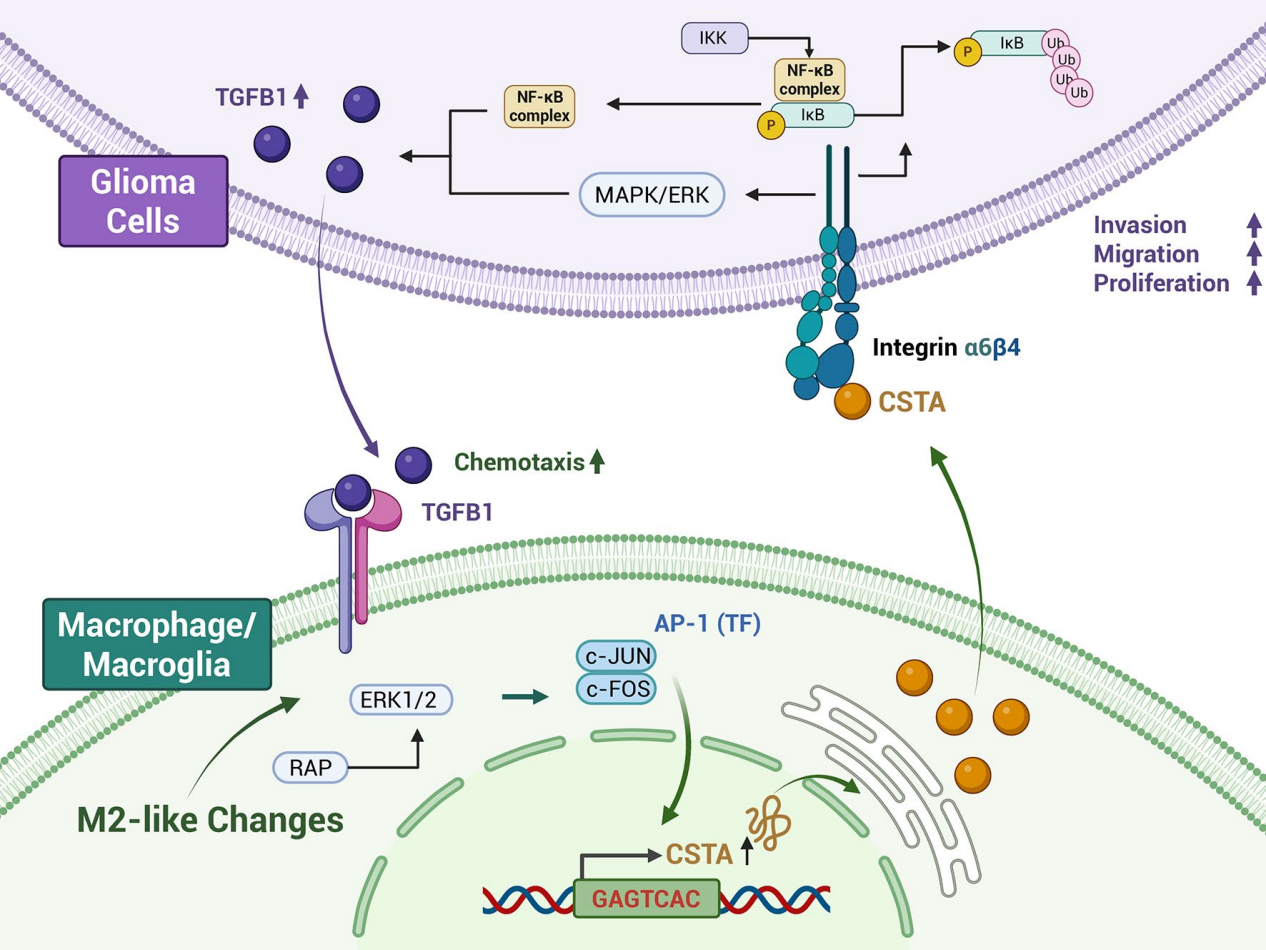

**Supplementary Information:**

The online version contains supplementary material available at 10.1186/s12967-026-08009-0.

## Introduction

Gliomas are the most common malignant tumors of the central nervous system, accounting for approximately 80% of all primary CNS malignancies. They are characterized by high aggressiveness, a propensity for diffuse invasion, high mortality, and resistance to complete eradication by standard therapies [1]. Approximately half of glioma patients have glioblastoma (GBM), which carries a dismal prognosis, with a 5-year survival rate of merely 2–10% [1, 2], and it is prone to developing resistance to the conventional treatment paradigm involving surgical resection combined with radiotherapy/chemotherapy due to factors like intratumoral heterogeneity and aberrant activation of DNA repair pathways [3]. Optimizing the clinical management of GBM remains a crucial objective in the field of neurosurgery. To this end, a deeper understanding of the mechanisms underlying the initiation and progression of gliomas is required.

The blood-brain barrier (BBB) confers unique immune privilege to the central nervous system (CNS) by restricting lymphocyte infiltration, establishing microglia as primary immune sentinels under physiological conditions [4]. Given their high abundance (≈ 15–20% of CNS cells), lymphocytes recruited following BBB disruption in gliomas are vastly outnumbered by these resident microglia and infiltrating macrophages (collectively termed GAMs), which constitute 30–50% of the glioma core [5]. Consequently, dynamic phenotypic and functional shifts in these glioma-associated macrophages/microglia (GAMs) play a pivotal role in regulating glioma initiation and progression. GAMs display high plasticity during this process [6]. In early-stage tumors, some GAMs exert transient anti-tumor effects by phagocytosing abnormal cells; however, as the tumor microenvironment deteriorates (e.g., under hypoxia or metabolite accumulation), they gradually polarize toward a pro-tumor phenotype [7, 8]. This entails functional shifts, including VEGF secretion to promote angiogenesis and MMPs release to degrade extracellular matrix and enhance invasiveness [9, 10]. Furthermore, secretory crosstalk between GAMs and glioma cells directly modulates the malignant phenotype of glioma cells. Canonical molecules such as IL-10 and TGF-β not only reinforce pro-tumor phenotype of GAMs but also directly drive glioma cell proliferation, stemness, and chemo-radiotherapy resistance [11]. GAMs-derived VEGF sustains glioma stem cell stemness via vasculogenic mimicry, thereby promoting GBM progression [12]. These findings support a regulatory network mediated by soluble factor-dependent unidirectional and bidirectional signaling between glioma cells and GAMs. Despite advances in understanding such soluble factor-mediated interactions, the full complexity of the GAMs secretome, including understudied secreted proteins and their precise mechanisms, remains incompletely defined.

In this study, we identified 16 immune signature genes in glioma bulk RNA-seq cohorts using the least absolute shrinkage and selection operator (LASSO) regression and random forest methods, and based on these, pinpointed a prognosis-associated CSTA⁺ M2-like GAM subset in GBM scRNA-seq datasets. In vitro and in vivo models revealed that M2-polarized GAMs upregulate CSTA expression and secretion through the ERK/AP-1 axis. Affinity IP-mass spectrometry identified ITGB4 as the functional plasma membrane receptor of CSTA in GBM. The NF-κB and MAPK are downstream signaling pathways of the CSTA-ITGB4 axis, which accelerates GBM progression and upregulates TGFB1 to form a positive feedback loop by recruiting/polarizing M2-like GAMs. Clinically, serum and cerebrospinal fluid (CSF) CSTA levels correlated positively with glioma grade, were higher in recurrent than primary tumors, and decreased post-surgery. Collectively, we uncovered a novel CSTA-ITGB4-TGFB1-mediated GBM-GAM crosstalk loop, highlighting CSTA as a promising biomarker with prognostic value and potential for monitoring therapeutic efficacy.

## Materials and methods

### Gene screening based on public databases

The approximate classification of these public datasets used in this study was as follows: (1) Bulk-/sc-RNAseq matrices and corresponding metadata; (2) Immune signature gene sets; (3) Nucleic acid and protein sequence information of different species. Detailed information was included in Supplementary table S1.

In the TCGA and CGGA cohorts, we first evaluated the effect of 210 immune signatures on overall survival (OS) time and status with the univariate Cox analysis. Next, we compared the hazard ratios of each signature and took their intersection. With the LASSO regression, the ImmSig was constructed:

$$\begin{aligned}ImmSig=&\sum_{i=1}^{n}Coef(Signature_{i})\cr&\mathrm{*}Enrichment\:fraction({Signature}_{i}).\end{aligned}$$

Parameters are included in Supplementary Table S2. The computational analyses in this section were conducted based on the “glmnet” R package [13].

A neural network built on the TCGA cohort served as the secondary classifier for ImmSig. With differentially expressed genes (DEGs) between high and low ImmSig subgroups, random forest was constructed based on the “randomForest” R package [14]. The branch number with minimized systematic errors was found by 10-fold cross-validation. Next, setting the Mean Decrease > 2 as the threshold, DEGs were screened for generating the neural network. The calculation of the output layer score included:

1. $$\begin{aligned}&\mathrm{s}\mathrm{c}\mathrm{o}\mathrm{r}\mathrm{e}(Hidden\:Layer)\cr&=\sum_{i=1}^{n}weight\:({Input\:Layer}_{i})\cr&\mathrm{*}Genescore\:({Input\:Layer}_{i});\end{aligned}$$
2. $$\begin{aligned}&\mathrm{s}\mathrm{c}\mathrm{o}\mathrm{r}\mathrm{e}\:(Output\:Layer)\cr&=\sum_{i=1}^{n}weight\:({Hidden\:Layer}_{i})\cr&\mathrm{*}score\:({Hidden\:Layer}_{i}).\end{aligned}$$

Relevant parameters are included in Supplementary Table S3. area under the curve (AUC) > 0.7 serves as the accuracy threshold.

### Statistical methods and software in bioinformatic analysis

Relevant methods and procedures from previous studies were referred to for the bioinformatics analysis [15, 16]. In our study, correlations between variables were verified with the Spearman correlation analysis. The Kaplan-Meier (K-M) curves were checked with log-rank test. The scRNA-seq data processing primarily relies on the “Seurat” package. During preprocessing, cells in the scRNA-seq cohort with gene count > 500, UMI > 2000, and mitochondrial gene count < 10% were retained; sequencing depth was then normalized with scale.factor = 10,000 and margin = 1, and the “FindVariableFeatures” function was used to identify the top 5000 most variable genes. Furthermore, the “RunPCA” function was applied to perform PCA on the gene count matrix with 50 computed dimensions, and the “harmony” package was finally adopted to integrate scRNA-seq matrices from different sample sources at lambda = 0.5 with 50 dimensions [17], thus completing the preprocessing workflow.

For clustering, the “FindNeighbors” function was used to analyze the harmony matrix, and the “FindClusters” function was applied at a resolution of 0.8 for dimensionality reduction and identification of single-cell subpopulations. Subsequently, the “FindAllMarkers” function with the Wilcoxon test was used to identify DEGs between subpopulations, with the logFC threshold of > 0.5. Cell type annotation was performed by comparing DEGs from subsets against the CellMarker database (http://117.50.127.228/CellMarker/) [18]. The “InferCNV” method was used to calculate copy number variation levels across cell subtypes [19]. In addition, pseudotime analysis was performed based on the “monocle3” package [20].

The R version used for analyzing and processing RNA-seq and scRNA-seq data was 4.1.2. The protein three-dimensional structures and interactions were simulated with the AlphaFold Server and visualized by PyMol software [21–23].

### Quantitative polymerase chain reaction (qPCR)

In this study, qPCR was used to determine RNA expression changes of target genes and quantify transcription factor-bound DNA sequences in chromatin immunoprecipitation-PCR (ChIP-PCR). Reagents, equipment and primer sequences were listed in Supplementary Table S4. In terms of statistical methods, GAPDH is the internal reference and adopts the 2^(-ΔΔCt) method to calculate differences in Ct values. In ChIP-PCR, the ratio of $2^{\wedge}(-{\Delta}{\Delta}\mathrm{C}\mathrm{t})(ChIP\:\&\:IgG)/2^{\wedge}(-{\Delta}{\Delta}\mathrm{C}\mathrm{t})\left(input\right)$ is used to calculate and compare the percentage ratio to the input.

### Western blotting (WB)

In WB, proteins of different molecular weights were separated by SDS-PAGE, transferred to PVDF membranes via wet transfer, then processed with blocking, antibody incubation and exposure. Band grayscale values were calculated via ImageJ. The differences in target proteins were quantified and compared with Relative ratio = Grayscale (target protein)/Grayscale (GAPDH/𝛽-tubulin). The relative ratio in the negative control was normalized to 1. Phosphorylation indices were calculated by Phosphorylation rates = Relativeratio (phosphorylation)/Relative ratio (overall). Detailed reagents, antibodies, and equipment are listed in Supplementary Table S5.

### Immunofluorescence (IF) staining

The GBM patient tissues (*N* = 5) used for IF staining were derived from our previous work [24]. For relevant ethical details, see Supplementary Table S13. The tyramide signal amplification method was used for IF staining. The fluorescence intensity of IF was quantified by ImageJ. The cell with the highest fluorescence intensity in the same field was set as 100% (reference value), and the intensities of other cells were calculated as ratios relative to this reference. Detailed information is listed in Supplementary Table S6.

### Enzyme-linked immunosorbent assay (ELISA)

ELISA was primarily used to detect CSTA concentrations in CSF and blood samples from clinical patients, as well as the CSTA concentration in the cell culture supernatant, employing the sandwich colorimetric assay. This was performed with the Human (LSBio, LS-F51739-1) and Mouse (LSBio, LS-F14618) CSTA ELISA kit, and the absorbance was measured with the Multiskan FC microplate reader (Thermo Fisher, 1410101). Six standard concentrations (0, 5, 10, 15, 20, 25 ng/mL) were prepared with recombinant CSTA protein (MCE, HY-P7868). A linear function of absorbance vs. CSTA concentration was fitted, with R² ≥ 0.99 as the criterion for valid linearity. The CSTA concentrations in clinical samples were calculated based on this.

### Cell line selection and culture

In this study, four macrophage/microglial cell lines and four glioma cell lines were included. Cells were cultured in complete medium under conditions of 37 °C and 5% CO₂. Detailed information is provided in Supplementary Table S7.

The M2 polarization induction conditions for different macrophage/microglia cell lines were shown in Supplementary Table S8.

### Transfection

Transfection was used to construct cell lines with target gene knockdown and overexpression, to perform transfection of luciferase reporter plasmids, or to enable target genes carrying Flag tags. The transfer of vector plasmids was performed by forming lentiviruses in 293T cells followed by infection of target cells. Knockdown or overexpression was performed using two parallel plasmids, and the cell line with the most significant changes at the RNA or protein level was selected for subsequent experiments. Results of the dual-luciferase reporter assay were recorded by a microplate reader, with the calculation formula as follows:

$$\begin{aligned}&Relativeluciferaseactivity\cr&=\frac{Fireflyluciferaseactivity\left(F\right)}{Renillaluciferaseactivity\left(R\right)}\end{aligned}$$

Relevant information is detailed in Supplementary Table S9.

### Cellular phenotypic experiment

Cellular phenotypic assays included the following: CCK8 assay was used to determine the optimal concentration and duration of CSTA stimulation; EdU and colony formation assays were employed to evaluate changes in proliferation; scratch assay was performed to assess alterations in migration ability; and Transwell assay was conducted to evaluate changes in invasion capacity. Related reagents and equipment are listed in Supplementary Table S10.

### Murine model construction

The murine in vivo model was established with male C57BL/6 mice in 3–6 weeks. After anesthesia with isoflurane in a gas chamber, the mice were subjected to hair removal on the cranial vertex. A xenograft glioma model was constructed by injecting 5 µl of cell suspension into the right prefrontal lobe. The total number of tumor-forming cells per mouse was 5 × 10⁴, consisting of 4 × 10⁴ CT2A cells and 1 × 10⁴ BV2 cells. For relevant ethical details, see Supplementary Table S13.

### In vivo imaging

In vivo imaging was used to dynamically evaluate the differences in the in vivo volume growth rate of gliomas under different treatment conditions. Detailed information is provided in Supplementary Table S11.

### Hematoxylin & Eosin (H&E) staining

Mouse brain tissues were fixed with 4% paraformaldehyde, then entrusted to Bioscience Co., Ltd. for paraffin embedding, sectioning, H&E staining, and scanning. Tumor volume was estimated based on the maximum diameter of the coronal section.

### In vivo experimental arrangement strategies

To ensure the rigor of the results, animal samples with the same intervention measures were further subdivided into two subgroups for different experiments.

1. Survival analysis cohort (*n* = 6): In vivo imaging was conducted on day 7 to confirm tumor formation. Subsequently, only their survival status was monitored. Euthanasia was carried out when any of the following euthanasia endpoints were met weight loss ≥ 20%, persistent neurological deficits, severe disturbance of consciousness or inability to eat/drink independently, persistent huddling, immobility, or unresponsiveness to stimuli.
2. Time-point analysis cohort (*n* = 3): In vivo imaging was performed on days 7, 14, and 21 after tumor implantation. The mice were sacrificed immediately after the final in vivo imaging session as scheduled. Whole brain specimens were collected for paraffin embedding, sectioning, and H&E staining. This subgroup was not included in the survival analysis.

### Immunoprecipitation (IP), Pull Down Assay, and Mass Spectrometry

Co-IP used Fc-tagged CSTA recombinant protein to obtain mass spectrometry samples for exploring its potential protein-protein interactions with plasma membrane surface proteins (affinity IP). ChIP was employed to acquire DNA fragments potentially bound to c-FOS, aiming to verify their binding to the CSTA promoter. Mass spectrometry analysis was commissioned to Meiji Biotechnology. In addition, the pull-down assay system was constructed based on a recombinant protein system. The Fc-CSTA and His-tagged ITGB4 proteins employed in the pull-down assay are recombinant proteins that were designed, expressed, and purified prior to the experiment. The assay was conducted in a cell-free system. In this study, the human and mouse-derived His-tagged recombinant ITGB4 truncation mutant proteins were commissioned to be synthesized by Pujian Biotechnology Co., Ltd. Details of reagents and equipment were provided in Supplementary Table S12.

### Clinical cohort

The human GBM specimens used in this study were sourced from patients with informed consent at Tongji hospital, Tonji medical college of Huazhong University of Science and Technology. Plasma and CSF sampling from GBM patients was conducted 3 days before and 1 week after surgery. Normal controls were derived from patients with simple hydrocephalus. Specimens were transported to the laboratory within 30 min in 4 °C insulated containers and immediately processed upon arrival. After use, they were stored long-term at -80 °C. For relevant ethical details, see Supplementary Table S13.

### Statistical information

Statistical analysis of molecular biology experimental results was primarily performed with GraphPad Prism 10. In terms of statistical methods, we first evaluated the data for normal distribution using the Shapiro-Wilk test and for homogeneity of variance using Levene’s test. For datasets that satisfied these assumptions, the t-test was used for comparisons between two groups, and one or two-way analysis of variance (One or two-way ANOVA) was used for comparisons among three or more groups. For data that did not meet these criteria, we employed non-parametric tests (such as the Mann-Whitney U test). For correlation analysis, the significance of the association between indicators was evaluated through scatter plots combined with linear regression. Survival analysis was based on the K-M method and log-rank test.

## Results

### CSTA^+^ M2-like GAMs are drivers for poor prognosis in GBM

To pinpoint microenvironmental factors tied to prognostic risk in gliomas, we first calculated enrichment fractions for 204 immune signatures using single-sample gene set enrichment analysis (ssGSEA) and its derived algorithms. Univariate Cox analysis identified 83 and 119 signatures with prognostic significance in TCGA and CGGA cohorts, respectively (Supplementary Table S14). Intersection analysis further narrowed these to 75 signatures with consistent prognostic relevance (Figure S1A). LASSO regression determined the optimal penalty coefficient (log(λ) = -3.7) (Figure S1B, S1C), enabling construction of ImmSig using 13 signatures with nonzero coefficients. Principal components significantly differed between high- and low-ImmSig subgroups (Figure S1D, S1E), and ROC curves validated the reliability of ImmSig as a classifier (Figure S1F, S1G). This indicated that ImmSig was a reliable prognostic stratification strategy.

**Fig. 1:**
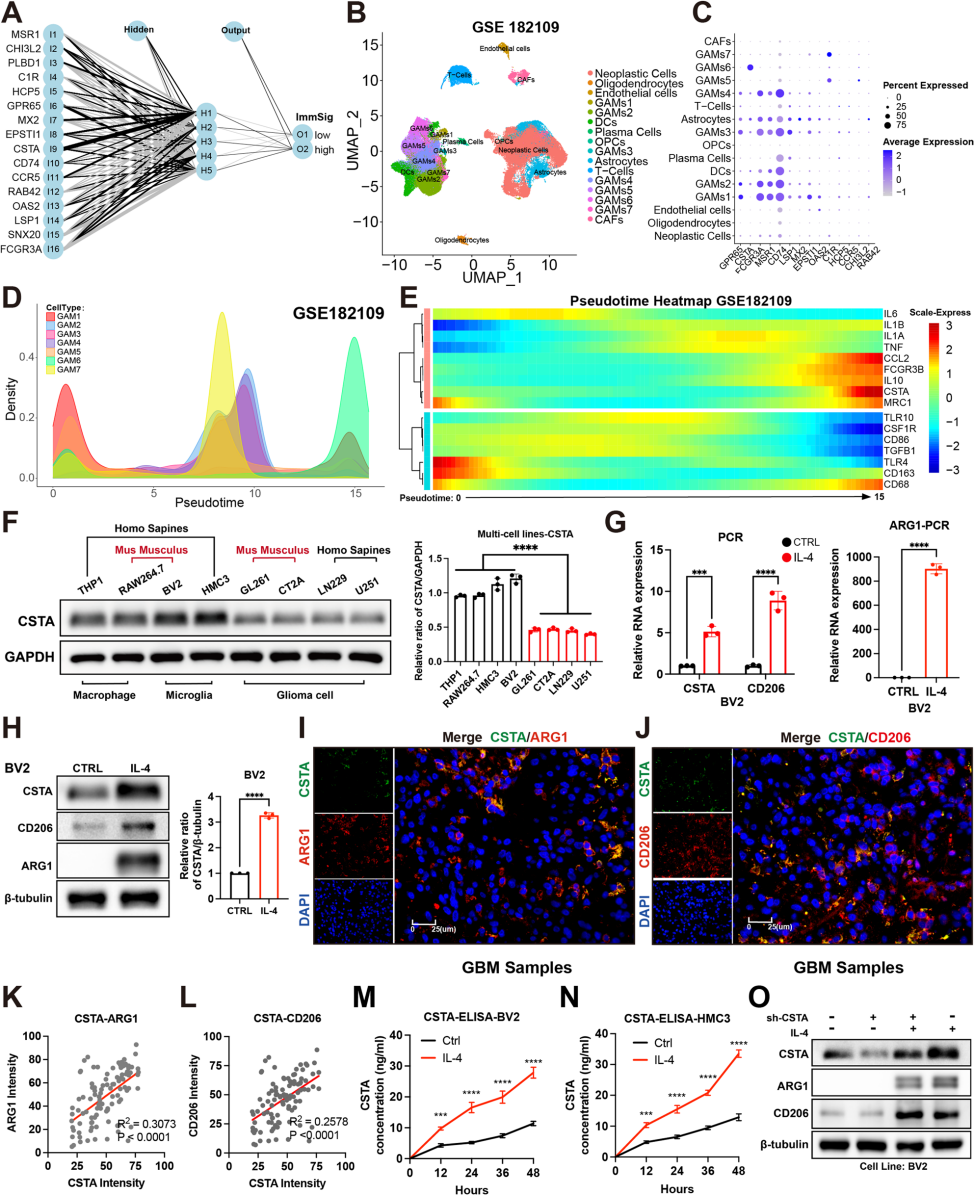
CSTA is a secretory signal of M2-like GAMs. (**A**) The neural network was constructed using the random forest method, comprising 16 immune signature genes as the input layer, 5 hidden layers, and an output layer. (**B**) UMAP illustrating the spatial distribution patterns of distinct cell subpopulations in GBM scRNA-seq data from GSE182109. (**C**) Differential expression profiles of immune signature genes across various cell subpopulations, where bubble size denotes the positive rate within each subpopulation and color intensity indicates the expression level. (**D**) Peak plot showing the distribution of different GAMs subpopulations along the pseudotime axis. (**E**) Differential expression patterns of M1/M2 macrophage markers along the pseudotime axis. (**F**) Differences in basal CSTA protein levels between GAMs and glioma cell lines. **G**,** H.** Differences in RNA (**G**) and protein (**H**) levels of CSTA, CD206, and ARG1 in BV2 cells with or without IL-4 treatment. **I**,** J.** Immunofluorescence (IF) co-localization results of ARG1 (**I**) and CD206 (**J**) with CSTA in GBM patient tissue sections. **K**,** L.** Correlation analysis of fluorescence intensity in co-localized cells between ARG1 (**K**) and CD206 (**L**) with CSTA in GBM patient tissue sections. **M**,** N.** ELISA assay showed the changes in CSTA concentration in the supernatants of BV2 (**M**) and HMC3 (**N**) cells with IL-4 induction time. **O**. Western blot (WB) results showing the effect of CSTA knockdown on protein levels of M2-like polarization markers ARG1 and CD206 in BV2 cells. *p < 0.05, **p < 0.01, ***p < 0.001, ****p < 0.0001

Next, the ImmSIg model was secondarily simplified via signature genes, and 949 DEGs were identified between high- and low-ImmSig subgroups in the TCGA cohort (Figure S1H, Supplementary Table S15). Ten-fold cross-validation set the optimal branch number at 150 (Figure S1I), and 16 signature genes were selected using a Mean Decrease > 2 threshold (Figure S1J); all were confirmed as prognostic risk factors (Figure S1K). Accordingly, these 16 genes constituted the input layer of the neural network (Fig. 1A), with receiver operating characteristic (ROC) curves validating the network as a robust secondary classifier (Figure S1L, S1M).

Given the heterogeneity among microenvironmental components, immune signature genes may exert their primary functions within specific cell subsets. Thus, we mapped their distribution into the scRNA-seq data from GBM. At a resolution of 0.5, 26 cell subsets were identified (Figure S2A), and the InferCNV analysis classified C1, C8, C9, C21, and C23 as neoplastic cells (Figure S2B). Combined with the CellMarker annotations, the cell subpopulation distribution is shown in Fig. 1B. Intersection with inter-subset DEGs retained 14 immune signature genes (Figure S2C), among which CSTA exhibited the most robust specificity, with high expression restricted almost exclusively to GAMs6 (Fig. 1C). Concomitantly, high CSTA expression correlated with poor prognosis in both LGG and GBM (Figure S2D–S2G). Pseudotime trajectory analysis of GAMs localized the GAMs6 subset to the terminal end, suggesting a highly polarized phenotype (Fig. 1D). Based on common macrophage polarization markers, the CSTA⁺ GAM6 subpopulation likely possesses an M2-like phenotype (Fig. 1E). Taken together, the CSTA⁺ M2-like GAMs subset may be one of the key microenvironmental drivers of poor prognosis in GBM.

### CSTA is a secreted signaling molecule of M2-like GAMs

Next, we validated the omics findings using in vitro cell models. GAMs exhibited significantly higher basal CSTA expression than GBM cells (Fig. 1F). Upon M2 polarization, CSTA’s RNA and protein levels increased markedly (Fig. 1G, H, S2H–S2J). In GBM tissues, CSTA co-localized with M2 polarization markers ARG1 and CD206, with their fluorescence intensities showing a significant positive correlation (Fig. 1I–L). Notably, among the pro-GBM GAM markers, the RNA and protein expression levels of SPP1 and TREM2 were also significantly upregulated upon IL-4 induction (Figure S3A-S3D). In GBM tissue sections, IF co-localization of CSTA with SPP1/TREM2 was also detected (Figure S3E), and a significant positive correlation was observed between their fluorescent intensities (Figure S3F, S3G), further confirming the presence of CSTA⁺ M2 GAMs with pro-GBM effects in the glioma microenvironment.

These results led to two functional hypotheses regarding CSTA: (1) it may act as a regulator of M2-like polarization; (2) CSTA may affect GBM cell phenotypes via paracrine signaling—a notion supported by previous reports that CSTA functions as a secretory protein [25, 26]. To test these hypotheses, we first characterized the secretory effect of CSTA. In the BV2 and HMC3 cell models, the level of CSTA in the supernatant exhibited a time-dependent increase and was significantly elevated following IL-4 induction (Fig. 1M, N). Second, we intervened in CSTA expression in GAMs (Figure S2K–S2N). CSTA suppression did not alter the basal phenotype of GAMs or their M2 polarization status (Fig. 1O, S3H–S3J). Since CSTA suppression did not affect M2 polarization, its secretory protein property suggests it may exert pro-GBM effects via paracrine action.

### CSTA acts as a key driver of glioma malignant progression

CSTA reportedly affects cancer cell phenotypes, like hepatocellular carcinoma recruits CSTA to enhance DNA damage repair and induce oxaliplatin resistance [27]. Thus, we investigated whether CSTA can similarly influence the malignant phenotypic repertoire of GBM.

**Fig. 2:**
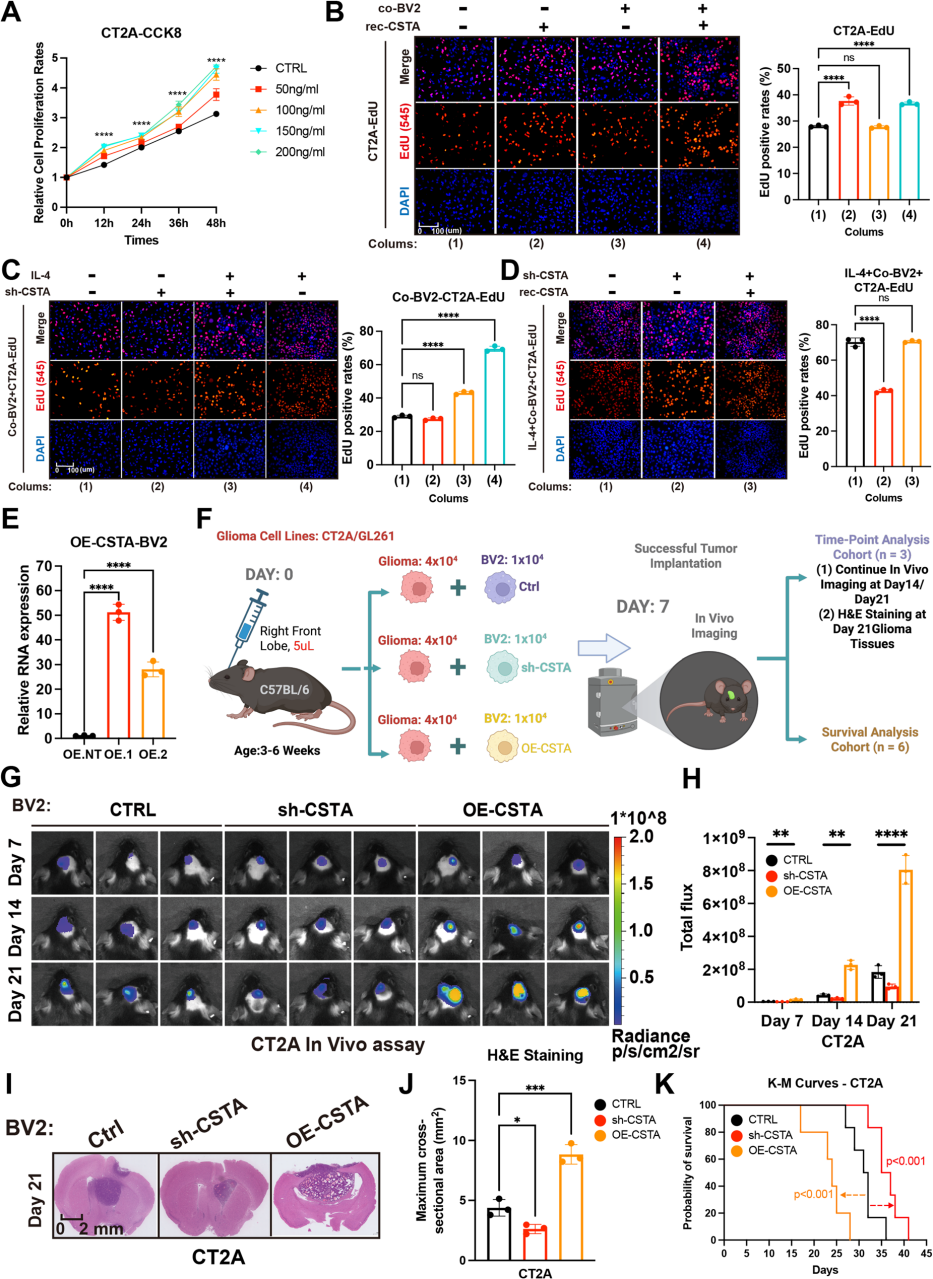
CSTA enhances GBM cells malignancy. **A.** The CCK-8 assay showing the correlation of CT2A cell proliferation rate with CSTA stimulation concentration and time. **B**,** C**,** D.** The EdU assay displaying the effect on CT2A proliferation in BV2-CT2A co-culture system under CSTA recombinant protein stimulation alone (**B**), CSTA intervention after BV2 M2-like polarization (**C**), and exogenous CSTA supplementation after BV2 M2-like polarization plus CSTA intervention (**D**). **E.** The RT-PCR results showing the difference in CSTA overexpression efficacy between two plasmids in BV2, OE.1 was selected to represent OE-CSTA. **F.** The schematic of in vivo animal model construction: Mice were divided into 3 subgroups based on CSTA interventions in mixed-implanted BV2 cells, including untreated control, CSTA-knockdown (sh-CSTA) and CSTA-overexpressing (OE-CSTA) groups. Each group was further divided into time-point analysis cohort (*N* = 3, for tracking tumor volume) and survival analysis cohort (*N* = 6, for survival information). **G**,** H.** In vivo imaging (**G**) and fluorescence readout statistics (**H**) at 7, 14 and 21 days in time-point analysis cohort in the CT2A-BV2 mixed tumor formation model. **I**,** J.** HE staining of mouse brain coronal sections (**I**) and maximum cross-sectional area statistics (**J**) at 21 days in time-point analysis cohort. **K.** Kaplan-Meier (K-M) curves showing survival time and status of different subgroups in survival analysis cohort of the CT2A-BV2 mixed tumor formation model. *p < 0.05, **p < 0.01, ***p < 0.001, ****p < 0.0001

To determine the optimal CSTA stimulation conditions for GBM cells, CCK-8 assays identified 100 ng/ml CSTA with 48-hour treatment as this condition for GBM cells (Fig. 2A, S4A). Proliferation was assessed via EdU and colony formation assays: in CT2A cells, CSTA stimulation increased EdU incorporation and colony formation, indicating enhanced proliferation (Fig. 2B, S4B). CSTA supression in M2-polarized BV2 cells reduced this effect (Fig. 2C, S4C), which was restored by exogenous CSTA supplementation (Fig. 2D, S4D). Scratch and Transwell assays confirmed CSTA also promoted GBM cell migration and invasion (Figure S4E–S4J). These findings were recapitulated across GBM cell lines including GL261, LN229, and U251, confirming that CSTA promotes proliferation, migration, and invasion in vitro (Figure S5–S7).

To validate CSTA’s in vivo pro-tumorigenic effect, we enforced its expression in BV2 cells and used an orthotopic glioma transplantation model (Fig. 2E). Figure 2F outlines subgroup interventions. In the time-specific cohort, elevated CSTA exerted a significant tumor-promoting effect, while its deficiency partially reduced tumor growth (Fig. 2G-J, S8B-S8E). In the survival analysis cohort, in vivo imaging on day 7 showed robust fluorescence in all groups, confirming successful implantation (Figure S8A). Consistent with tumor growth trends, elevated CSTA correlated with shorter survival (Fig. 2K, S8F). Collectively, CSTA exerted consistent pro-tumor effects in vitro and in vivo, functioning as a key driver of GBM malignant progression.

### CSTA is upregulated via ERK/AP-1 axis in M2-polarized GAMs

**Fig. 3:**
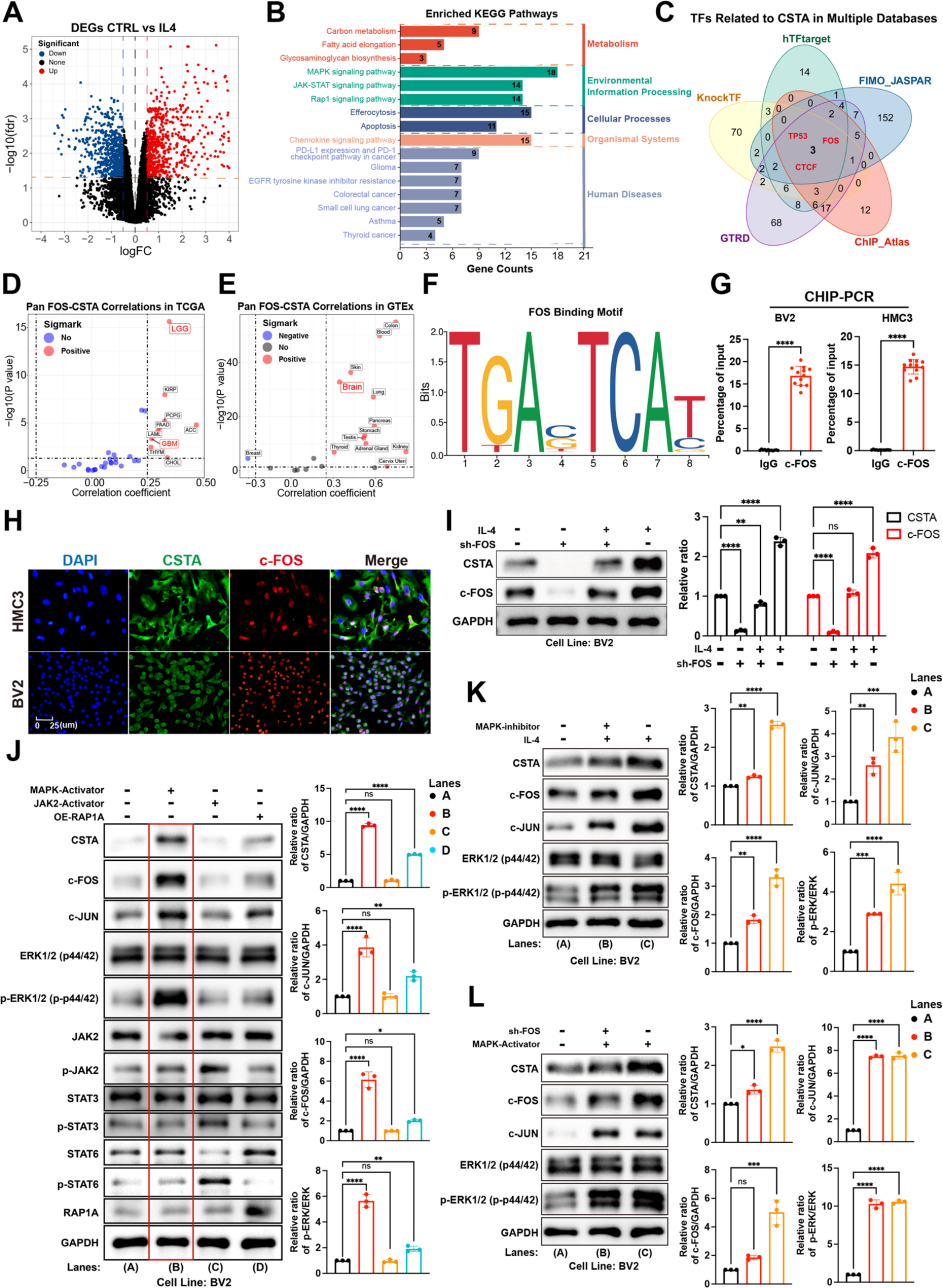
ERK/AP-1 axis promotes the expression of CSTA in GAMs. (**A**) In the GSE242543 cohort, the distribution of differentially expressed genes (DEGs) between human peripheral blood macrophages with and without IL-4 stimulation, with screening criteria set as |LogFC| ≥ 1 and FDR < 0.05. (**B**) KEGG functional enrichment analysis results of the DEGs above. (**C**) The intersection of prediction results for potential transcription factors of CSTA from hTarget, FMO_JASPA, KnockTF, GTRD, and Chip_Atlas databases, retaining three potential transcription factors: TP53, FOS (c-FOS), and CTCF. **D**,** E.** Correlation tests between c-FOS and CSTA in TCGA pan-cancer (**D**) and GTEx normal human tissue (**E**) RNA-seq cohorts, where red dots indicate a significant association (*p* < 0.05 and correlation coefficient ≥ 0.25). **F.** Motif diagram of the potential binding base sequence of c-FOS in the CSTA promoter region. **G.** Results of ChIP-PCR for c-FOS and CSTA promoter fragments in BV2 and HMC3 cells, statistically analyzed based on relative expression compared to the Input group. **H.** Co-localization of c-FOS and CSTA by immunofluorescence (IF) staining in BV2 and HMC3 cell climbing sheets after IL-4-induced polarization. **I.** Effect of c-FOS knockdown on CSTA protein levels in IL-4-induced BV2 cells. **J.** Changes in protein levels of CSTA, c-JUN, c-FOS, and phosphorylated signaling molecules in BV2 cells after separate activation of MAPK, JAK/STAT, and RAP1 signaling pathways. **K**,** L.** Changes in protein levels of CSTA, c-JUN, c-FOS, and phosphorylated signaling molecules in BV2 cells after simultaneous MAPK inhibition and IL-4 induction (**K**), as well as after simultaneous c-FOS knockdown and MAPK activation (**L**). *p < 0.05, **p < 0.01, ***p < 0.001, ****p < 0.0001

Now that it has been established that CSTA is a component of the pro-tumorigenic effects of M2-like GAMs, this raises a new question: by what mechanism is CSTA regulated. The GSE242543 cohort provides comparative RNA-seq data of bone marrow-derived macrophages before and after IL-4 stimulation, and 839 DEGs were identified between the control and IL-4 subgroups (Fig. 3A). Three environmental information processing pathways were significantly enriched in these DEGs, with the MAPK showing the highest enrichment rate (Fig. 3B). Next, after intersected potential transcription factors (TFs) of CSTA in the five databases, TP53, FOS, and CTCF were retained (Fig. 3C). We noted that FOS (i.e., c-FOS) can form as heterodimeric AP-1 transcription factor complex with c-JUN, acting as a downstream effector of the MAPK [28]. Additionally, the levels of CSTA and FOS are significantly positively correlated in all the LGG, GBM, and normal brain tissues (Fig. 3D, E). Based on the binding motif of c-FOS, the potential binding sequence in the CSTA promoter region might be GAGTCAC in humans and TGACTC in mice (Fig. 3F). CHIP-PCR analysis revealed this (Fig. 3G). In IL-4-treated BV2 and HMC3 cells, c-FOS and CSTA exhibited a high degree of co-localization (Fig. 3H). To further clarify their regulatory relationship, c-FOS was inhibited (Figure S9A-S9D). The expression of CSTA was significantly reduced after that (Fig. 3I, S9E).

To further validate the contribution of each environmental information processing pathway to the CSTA levels, we individually activated each of them. The MAPK and JAK/STAT signaling pathways were activated via agonist stimulation, whereas activation of the RAP1 signaling pathway was achieved through upregulation of RAP1A (Figure S9F, S9G). The protein levels of CSTA, c-JUN, and c-FOS increased most significantly when the MAPK was engaged. Induced RAP1 signaling pathway could also slightly upregulate these genes, accompanied by a mild increase in the phosphorylation level of p44/p42 (Fig. 3J, S9H). The RAP1 signaling pathway can activate p44/p42 phosphorylation via the bypass mechanisms [29–31]. Thus, the p44/p42 phosphorylation might be the key regulatory signal for CSTA. Furthermore, inhibiting MAPK activity in M2 could significantly downregulate the CSTA (Fig. 3K, S9I). However, the elevation degree of CSTA was limited when the MAPK was induced in GAMs with c-FOS blockade. (Fig. 3L, S9J).

In summary, the MAPK signaling pathway, via AP-1, plays a pivotal role in regulating CSTA expression, with RAP1 potentially exerting a secondary effect through p44/p42 cross-activation.

### ITGB4 is the surface receptor of CSTA on glioma cells

**Fig. 4:**
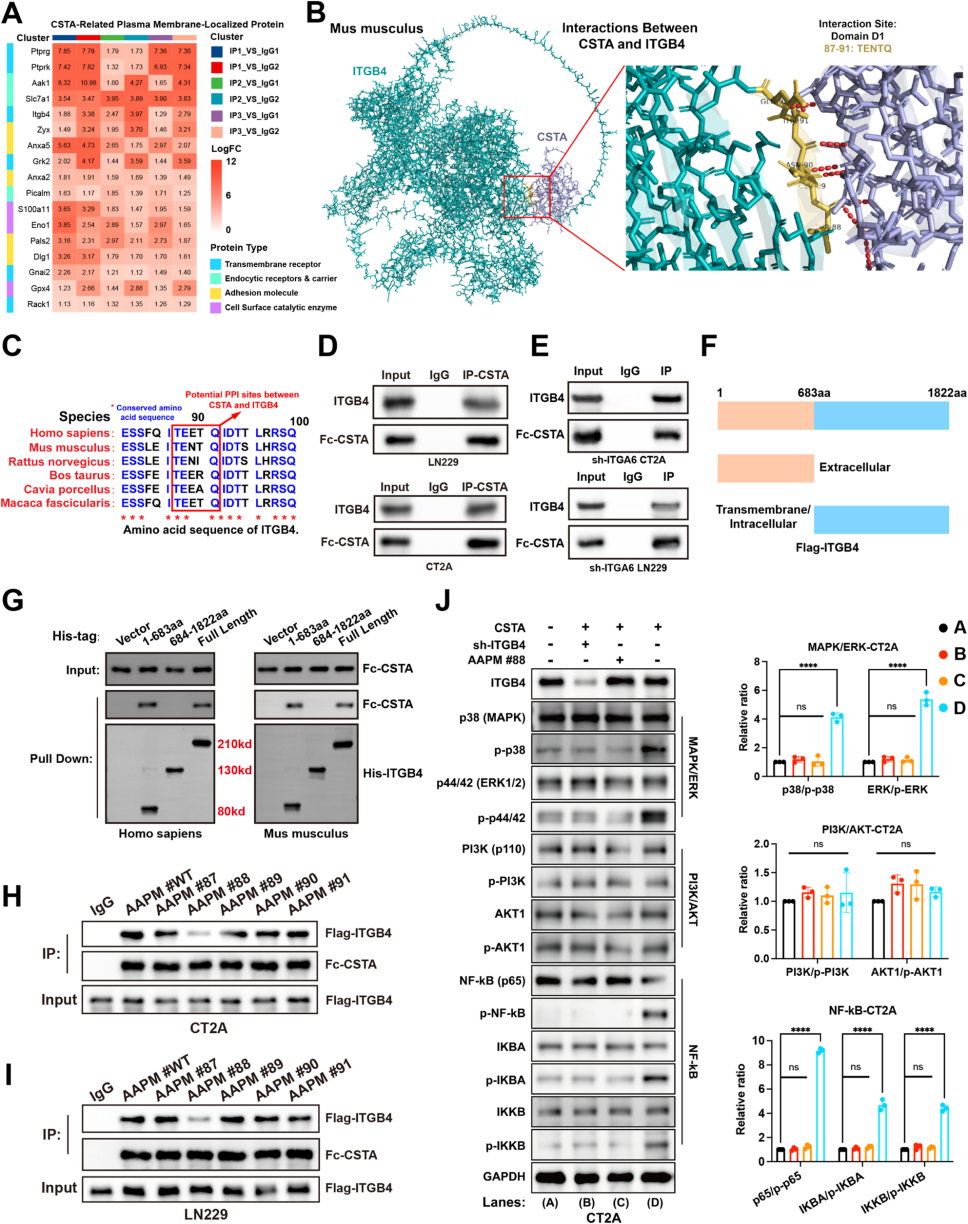
ITGB4 is the functional receptor of CSTA. (**A**) Affinity IP-mass spectrometry to identify glioma plasma membrane proteins potentially binding to CSTA; performed in CT2A membrane protein extracts, including 6 datasets (3 IP vs. 2 IgG, one-to-one comparisons). After intersection, Robust Rank Aggregation (RRA) ranking was applied (top to bottom = high to low RRA rank), with color intensity indicating LogFC levels in corresponding comparisons. (**B**) Molecular docking prediction of murine CSTA and ITGB4, enlarged area shows potential binding sites and corresponding amino acid residues. (**C**) Alignment of ITGB4 amino acid residues 80–100 across species, red box = residues 87–91 co-predicted to interact with CSTA in humans and mice via molecular docking, blue = conserved residues. (**D**) IP validation of CSTA-ITGB4 interaction in CT2A and LN229 cells. (**E**) IP validation of CSTA-ITGB4 interaction in CT2A and LN229 cells with ITGA6 knockdown. (**F**) Brief summary of ITGB4 truncation mutant design: residues 1–683 aa encode the extracellular domain, and residues 684–1822 aa encode the transmembrane and intracellular domains. (**G**) Pull down assay results of His-tagged human and murine ITGB4 truncation mutants and Fc-CSTA recombinant protein. **H**,** I.** Binding efficiency of Fc-CSTA to different ITGB4 AAPM mutants in CT2A (**H**) and LN229 (**I**) cells. **J.** Phosphorylation levels of signaling molecules in ITGB4-related pathways (MAPK, PI3K/AKT, NF-κB) in CT2A cells after CSTA stimulation, and effects of AAPM #88 and sh-ITGB4. *p < 0.05, **p < 0.01, ***p < 0.001, ****p < 0.0001

Given the paracrine property of CSTA, GBM likely relies on membrane receptors to receive its pro-tumorigenic signals—an aspect explored via affinity IP and mass spectrometry. Via Robust Rank Aggregation (RRA) method, PTPRG, PTPRK, AKA1, SLC7A1, and ITGB4 had highest rank (Fig. 4A). Only ITGB4 was consistently a prognostic risk factor in TCGA, CGGA, CPTAC, and Gravendeel cohorts (Figure S10). Thus, ITGB4 was selected for subsequent studies. The interaction sequences of ITGB4-CSTA in both human and murine include ITGB4 amino acids 87–91 (Fig. 4B, S11A), which contains three conserved amino acid residues specifically threonine (T) at 87, glutamate (E) at 88, and glutamine (Q) at 91 (Fig. 4C). Co-IP assays confirmed the interaction between CSTA and ITGB4 across species (Fig. 4D). Second, ITGB4 typically forms a heterodimer with ITGA6 on the plasma membrane to exert its biological functions. To rule out the potential effect of ITGA6 on CSTA binding, CT2A and LN229 cell lines with shITGA6 were established (Figure S11B, S111C). The results of IP demonstrated that the protein-protein interaction between CSTA and ITGB4 was still observed in ITGA6-deficient cells (Fig. 4E). Thirdly, based on molecular docking results, the potential binding region of CSTA on ITGB4 was identified as amino acid residues 87–91, which is located in the extracellular domain (1–683 aa) of ITGB4. To verify this finding, ITGB4 truncation mutants were designed and constructed (Fig. 4F). Pull down assays revealed that CSTA interacted with the full-length and extracellular domain of ITGB4 but not with its transmembrane/intracellular domain (Fig. 4G). These results thus confirm that CSTA binds to the extracellular domain of ITGB4. Next, to map the key amino acid residues mediating the ITGB4-CSTA interaction, cell lines with Flag-tagged ITGB4 and its amino acid point-mutant (AAPM) variant were established (Figure S11D, S11E). Following the E88 AAPM, the binding efficiency between CSTA and ITGB4 significantly decreased (Fig. 4H, I). Thus, E88 of ITGB4 was the key residue mediating its specific interaction with CSTA.

Ample evidence indicates that ITGB4 mainly heterodimerizes with ITGA6 to form functional α6β4 integrin at the plasma membrane, where it serves as a critical node coordinating MAPK, PI3K/AKT, and NF-κB signaling networks [32–34]. To clarify the specific mechanism of downstream signal transduction and verify the uniqueness of ITGB4 as CSTA’s functional receptor, we intervened ITGB4 in GBM (Figure S11F, S11G). The CSTA-ITGB4 axis could enhance the phosphorylation levels of MAPK and NF-κB signaling molecules. Both inhibition of ITGB4 or AAPM at its E88 site could block the generation of downstream signals (Fig. 4J, S11H). Inhibition of downstream MAPK and NF-κB signaling partially reduced the CSTA-induced upregulation of proliferation, invasion and migration capacities in GBM cells (Figure S12). In addition, to exclude the effects of other integrin family proteins that also form heterodimers with ITGA6, the ITGB1 inhibition system was established (Figure S11I, S11J). Similar to ITGB4 intervention, ITGA6 inhibition also abrogated the transduction of CSTA downstream MAPK and NF-κB signaling, whereas ITGB1 intervention exerted no significant effect (Figure S11K, S11L). Finally, functional validation was performed at the cellular level. As shown in Figure S13, ITGA6 inhibition abrogated CSTA-mediated pro-GBM proliferation, invasion and migration, whereas ITGB1 inhibition had no significant effect. These results demonstrate that CSTA signal transduction in GBM cells is dependent on the α6β4 integrin as its receptor.

In summary, CSTA primarily binds to the E88 residue of ITGB4 and transduces the malignant signal to GBM cells by activating the downstream MAPK and NF-κB signaling pathways.

### ITGB4 blockade attenuates the pro-tumorigenic activity of CSTA

Compared with soluble CSTA, its receptor ITGB4, constitutively expressed on glioma cell membranes, is more readily targetable for intervention. Hence, its feasibility was first validated in in vitro models. Regardless of whether exogenous CSTA is administered, the ITGB4 intervention subgroups consistently exhibit a significant inhibitory effect on GBM proliferation (Figure S14A, S144D). The invasive and migratory abilities of GBM also exhibited the same trend (Figure S14B, S14C, S14E, S14F). In LN229 cells, identical experiments yielded consistent patterns (Figure S14G-S12L). Thus, across species, ITGB4 inhibition appears more effective than CSTA depletion. Given the diversity of ligands for the integrin family, ITGB4 inhibition also blocks pro-tumor signals from ITGB4 binding to ligands beyond CSTA [35, 36].

**Fig. 5:**
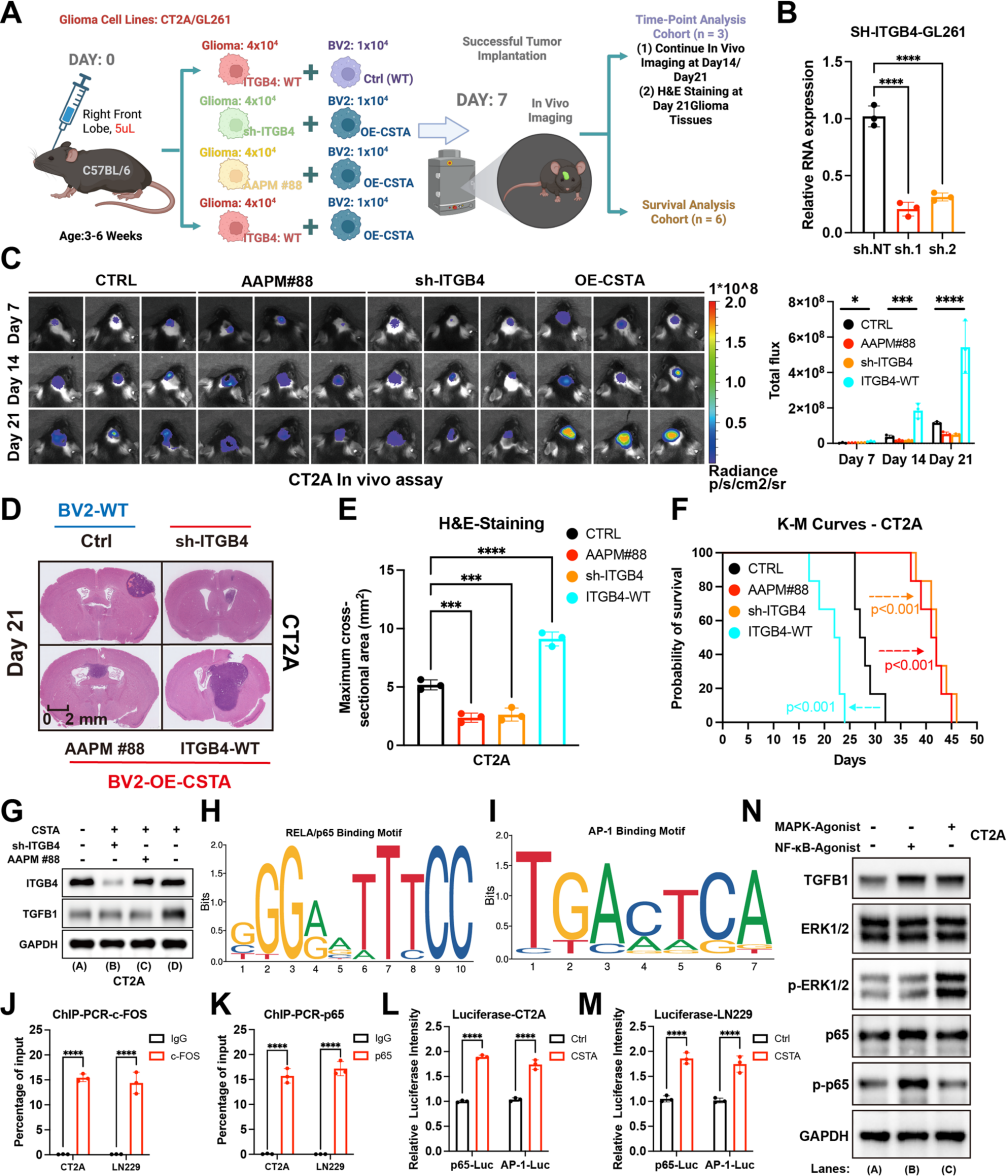
ITGB4 intervention inhibits CSTA-related GBM malignancy. (**A**) Schematic of in vivo ITGB4 intervention experiments: Four groups including CT2A/GL261 (ITGB4 WT) + BV2 WT (negative control), CT2A/GL261 (ITGB4 WT) + BV2 OE-CSTA (positive control), and CT2A/GL261 (ITGB4 AAPM #88 or sh-ITGB4) co-cultured with BV2 OE-CSTA. Each group was further divided into time-point analysis cohort (*N* = 3, for tracking tumor volume) and survival analysis cohort (*N* = 6, for survival information). (**B**) Validation of ITGB4 knockdown in GL261 cells (**C**) In vivo imaging (**H**) and fluorescence readout statistics at 7, 14 and 21 days in time-point analysis cohort in the CT2A-BV2 mixed tumor formation model. **D**,** E.** HE staining of mouse brain coronal sections (**D**) and maximum cross-sectional area statistics (**E**) at 21 days in time-point analysis cohort. **F.** Kaplan-Meier curve showing survival time and status of different subgroups in survival analysis cohort of the CT2A-BV2 mixed tumor formation model. **G.** Protein levels of TGFB1 in CT2A cells after CSTA stimulation, and effects of AAPM #88 and sh-ITGB4. **H**,** I.** Promoter-binding motifs of RELA/p65 (**H**) and AP-**1** (**I**). **J**,** K.** ChIP-PCR validation results for c-FOS (**J**) and p65 (**K**) binding to the TGFB1 promoter region in CT2A and LN229 cells. **L**,** M.** Dual-luciferase reporter assay results demonstrated the differences in the activities of AP-1 and p65 transcription factors in CT2A (**L**) and LN229 (**M**) cells before and after CSTA induction. **N.** Differences in the phosphorylation of key proteins of the relevant signaling pathways and TGFB1 protein levels in CT2A cells following treatment with MAPK and NF-κB signaling pathway agonists. *p < 0.05, **p < 0.01, ***p < 0.001, ****p < 0.0001

Next, the reliability of ITGB4 intervention in vivo was investigated, with key strategies detailed in Fig. 5A. AAPM E88 and sh-ITGB4 GL261 for modeling were constructed synchronously to ensure experimental consistency (Fig. 5B, S15A). In vivo imaging at day 7 in the survival analysis cohort confirmed the successful establishment of a mouse orthotopic GBM xenograft model (Figure S15B). In the time-specific analysis cohort, ITGB4 suppression or AAPM E88 each significantly retarded GBM growth kinetics in vivo. (Fig. 5C, S15C, S15D). Consistently, HE staining demonstrated that these two subgroups had the smallest final tumor volumes when sacrificed on day 21 (Fig. 5D and E, S15E, S15F). In the survival analysis cohort, glioma implantation succeeded, and ITGB4 intervention significantly prolonged the average survival time and increased survival rates of GBM mice (Fig. 5F, S15G).

In summary, intervening in ITGB4 blocks glioma cells’ response to CSTA at the cellular level and exerts robust in vivo inhibitory effects, with consistency across species.

### The CSTA-ITGB4 axis recruits GAMs via TGFB1

**Fig. 6:**
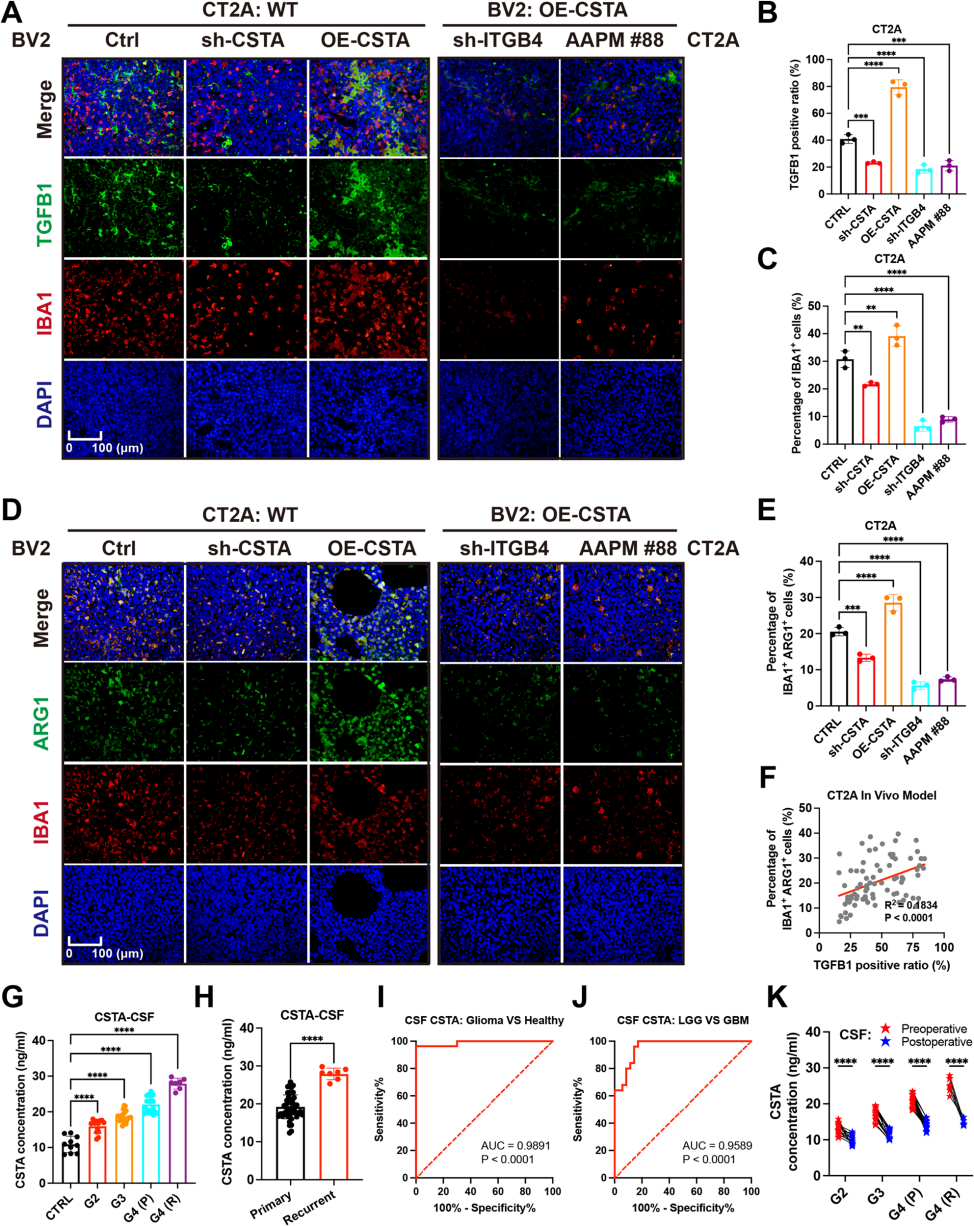
The CSTA-ITGB4-TGFB1 loop and the biomarker potential of CSTA. **A**,** B**,** C.** IF staining of TGFB1 and macrophage/microglia marker IBA1 in brain sections from CT2A/BV2 mixed orthotopic GBM xenograft models (**A**), with statistics on the proportions of TGFB1+ (**B**) and IBA1+ (**C**) cells among total cells across groups. **D**,** E.** IF staining of IBA1 and M2 polarization marker ARG1 (**D**), and statistics on the proportion of IBA1 + ARG1+ cells relative to total cells in the visual field across groups (**E**). **F.** Correlation between TGFB1 + and IBA1 + ARG1+ cell proportions, with each point representing values from an entire field. **G.** CSTA concentrations in cerebrospinal fluid (CSF) samples from patients with different glioma grades. **H.** CSTA concentrations in preoperative CSF samples from primary vs. recurrent glioma patients. **I**,** J.** ROC analysis of CSF CSTA concentrations in glioma vs. normal patients (**I**) and in LGG (G2, G3) vs. GBM (G4) patients (**J**), with corresponding AUC values of 0.9891 and 0.9589. **K.** Paired t-test of CSF CSTA concentrations in patients with different glioma grades before and 1 week after surgery. *p < 0.05, **p < 0.01, ***p < 0.001, ****p < 0.0001

Notably, NF-κB and MAPK signaling can potentially regulate TGFB1 expression, which can recruit GAMs into the microenvironment to promote glioma progression [37–39]. To this end, we further explored whether the CSTA-ITGB4 axis exerted similar changes. CSTA stimulation significantly upregulated TGFB1 protein levels in CT2A and LN229 cells, whereas ITGB4 blockade or AAPM E88 treatment abolished this effect (Fig. 5G, S15H). Next, potential promoter-binding sequences of AP-1 and RELA/p65, the downstream transcription factors of the MAPK and NF-κB signaling pathways, were identified in the FIMO_JASPAR databases (Fig. 5H, I). ChIP-PCR assays confirmed the binding of AP-1 (human: TGACTCG; mouse: TGAAGCA) and RELA/p65 (human: TTCACTTCCC; mouse: TGGGGATCC) to the TGFB1 promoter region (Fig. 5J, K). Dual-luciferase reporter assay results further demonstrated that the transcriptional activities of AP-1 and RELA/p65 were upregulated in GBM cells upon stimulation with recombinant CSTA protein (Fig. 5L, M). In addition, TGFB1 expression was increased in GBM cells following treatment with MAPK and NF-κB agonists (Fig. 5N, S15I). Immunofluorescence staining of mice model tissue sections revealed that reducing CSTA expression or interfering with ITGB4 function decreased the TGFB1 positive rates, while increasing CSTA expression in BV2 cells enhanced this (Fig. 6A and B, S16A, S16B). Similarly, intervening in ITGB4 yields better results than inhibiting CSTA itself. Additionally, Samples with high TGFB1 positive rates tend to have a higher percentage of IBA1^+^ cells. (Fig. 6C, S16C). This indicated that the CSTA-ITGB4 axis could recruit GAMs into tumor tissues by upregulating TGFB1. Further, the level of M2 polarization was quantified by the proportion of ARG1^+^ IBA1^+^ cells, which showed a similar trend across different subgroups as TGFB1 (Fig. 6D and E, S16D, S16E). Finally, the correlation between TGFB1 and M2 polarization level was calculated, manifested as a significant positive correlation (Fig. 6F, S13F).

Overall, TGFB1-related glioma-GAM positive feedback loop existed downstream of the CSTA-ITGB4 axis. This facilitates the chemotaxis of M2 GAMs into the microenvironment, exacerbating the immunosuppression in gliomas.

### CSTA is a biomarker for glioma prognosis and therapeutic efficacy

CSTA is a secreted protein already detected in the serum of patients with diseases such as atopic dermatitis, hepatocellular carcinoma, and liver cirrhosis, which supports its potential as a clinical biomarker [27, 40]. Given that inflammatory responses may affect CSTA levels [41], we measured CSTA concentrations in the serum and CSF of 10 patients with simple hydrocephalus (serving as non-tumor controls) and 55 glioma patients (including 7 cases of recurrent glioma), with all samples processed under identical conditions. The results showed that preoperative CSTA concentrations in both CSF and serum increased progressively with higher glioma grades (Fig. 6G, S16G). Additionally, CSTA levels were significantly higher in patients with recurrent glioma than in those with primary glioma (Fig. 6H, S16H). ROC analysis was then performed to assess the relationship between CSTA and glioma occurrence as well as grading. For distinguishing tumor from non-tumor samples, the AUC values for CSF and serum CSTA were 0.9891 and 0.9873, respectively (Fig. 6I, S16I). When comparing LGG (G2, G3) with GBM (G4), the corresponding AUC values were 0.9589 and 0.9653 (Fig. 6J, S16J). Finally, preoperative and postoperative CSTA concentrations were analyzed. A significant decrease in CSTA levels was observed postoperatively across all glioma grades (Fig. 6K, S16K). In summary, CSTA was closely associated with the occurrence and malignancy of glioma, and could be used to assist in diagnosis, assess disease progression, and evaluate postoperative efficacy.

## Discussion

This study is the first to identify CSTA as a novel information mediator between GAMs and glioma cells, providing new insights into glioma microenvironment regulation. As the most abundant immune population in the TME, GAMs functionally drive glioma progression [6], supported by our scRNA-seq cohort analysis, where UMAP visualization confirmed GAMs as a large subset. Prior studies have established that M2-polarized GAMs promote tumor growth, angiogenesis, and immune evasion via secreted cytokines, chemokines, and proteases [7, 8, 12, 42, 43]. Given the robust secretory capacity of GAMs, the pro-tumor effects of M2-like GAMs may not be entirely mediated by the canonical secretory factors, leaving gaps in the identification of their effector molecules. Therefore, this study first employed bioinformatics approaches for exploration and successfully identified a CSTA⁺ M2-like GAMs subset in the GBM microenvironment. This finding was validated in both in vitro models and GBM tissue sections.

Previous studies have identified CSTA as a small secretory protein [25, 26]. To explore its pro-tumor mechanisms, we proposed two hypotheses: (1) CSTA may regulate M2 polarization of glioma-associated macrophages (GAMs), analogous to how macrophage-derived autocrine CCL15 sustains M2 polarization via CCR1-PI3K-AKT signaling to promote immunosuppression [44]; (2) CSTA may act directly on glioma cells in a paracrine manner, similar to its role in promoting epithelial-mesenchymal transition and poor prognosis in colorectal cancer [45]. To test these, we manipulated CSTA in GAMs. Suppression of CSTA did not significantly alter M2 marker expression in GAMs (ARG1, CD206). Conversely, exogenous CSTA supplementation markedly enhanced glioma cell proliferation, migration, and invasion in vitro. Inhibiting CSTA expression in M2 GAMs attenuated their inherent pro-tumor effects, while in vivo, CSTA levels significantly negatively correlated with glioma volume and prognosis. These findings in glioma supported the hypothesis 2: CSTA, as a downstream effector of M2 GAMs, exerted pro-tumor effects by directly acting on glioma cells.

To dissect the mechanism of CSTA upregulation in M2 GAMs, the KEGG functional enrichment analysis of IL-4-stimulated versus unstimulated cells identified differential activation of MAPK, PI3K-AKT, and RAP1 signaling pathways. In vitro validation revealed robust CSTA induction via MAPK activation and moderate upregulation through RAP1, with crosstalk occurring at ERK1/2 (p44/p42). Mechanistically, the RAP1 effector B-Raf phosphorylates ERK1/2, thereby activating a parallel RAP1/MAPK axis [29–31]. Both MAPK agonism and RAP1A overexpression increased ERK1/2 phosphorylation, establishing ERK1/2 as a key regulator of CSTA. Bioinformatics and ChIP-PCR confirmed that c-FOS, an AP-1 component and MAPK effector, binds motifs GAGTCAC of humans and TGACTC of mice in the CSTA promoter. Collectively, co-localization of c-FOS and CSTA in M2 GAMs and their coordinate upregulation during polarization or MAPK activation implicated the ERK/AP-1 axis. Pharmacological MAPK inhibition or c-FOS knockdown abolished CSTA induction, establishing ERK/AP-1 as the critical regulatory node governing CSTA expression in M2 GAMs.

To address the gap in knowledge of CSTA’s membrane receptors, we identified ITGB4 as a putative receptor via affinity IP coupled mass spectrometry screening, prioritized by integrating survival analyses across multi-database glioma cohorts to focus on prognostically relevant molecules. ITGB4 forms the α6β4 integrin with ITGA6 at the cell membrane, exerting dual functions: mediating cell-matrix adhesion (e.g., in breast cancer, α6β4 promotes in situ growth by anchoring to the basement membrane, while its dynamic disassembly/re-adhesion enables metastatic penetration of basement membranes and vascular walls [46]); and acting as a receptor for extracellular ligands to activate downstream pathways (NF-κB, PI3K/AKT, MAPK) [32–34]. In gliomas, it maintains stemness and drives temozolomide resistance [47, 48]. To define the CSTA-ITGB4 interaction, molecular docking identified ITGB4 residues 87–91 as a potential binding region; point mutation and Co-IP assays confirmed E88 as the critical binding residue. We further confirmed that the CSTA-ITGB4 axis promotes glioma malignancy by activating NF-κB and MAPK signaling. Whether in vitro or in vivo, ITGB4 inhibition suppressed GBM malignancy and improved prognosis.

Notably, CSTA stimulation upregulated TGFB1 in glioma cells. TGFB1 is a key downstream effector of both NF-κB and MAPK signaling pathways [37, 38]. It can both recruits GAMs to the glioma microenvironment and induce their M2 polarization [49, 50]. In this study, IBA1⁺ GAMs and ARG1⁺IBA1⁺ M2-like cells in in vivo models were significantly increased when co-implanting OE-CSTA BV2 cells. Inhibition of ITGB4 reduced TGFB1 expression and secretion, as evidenced by decreased accumulation of GAMs in the microenvironment. Collectively, we identified a novel GAM-glioma crosstalk loop: M2-like GAMs secrete CSTA via ERK/AP-1 upregulation; CSTA activates NF-κB/MAPK via ITGB4 (E88-dependent) to promote malignancy. In turn, downstream TGFB1 recruits GAMs and polarizes them to M2. This positive feedback loop critically drives glioma progression and immunosuppressive microenvironment evolution.

Building on the mechanistic findings, we further explored the clinical relevance of CSTA. The blood and CSF CSTA levels in glioma patients correlate positively with tumor grade, supporting its potential as a non-invasive diagnostic and prognostic marker. Current clinical markers like molecular (IDH1/2 mutation, 1p19q co-deletion) and imaging-based (size, location) reflect only partial tumor cell traits, not the microenvironment comprehensively [51]. By contrast, CSTA integrates GAM function and tumor malignancy, with unique advantages to enhance the comprehensiveness and accuracy of glioma clinical management.

## Conclusion

In summary, our findings identify CSTA as a novel mediator between M2-like GAMs and glioma cells. M2-like GAMs secrete CSTA via the ERK/AP-1 regulatory axis. CSTA then binds ITGB4 (E88 as critical residue) on glioma cells to activate NF-κB and MAPK signaling, which enhances malignancy. Induced TGFB1 further recruits and polarizes GAMs toward M2-like changes, forming a pro-tumor positive feedback loop. Notably, blood/CSF CSTA levels correlate with glioma grade, implying non-invasive diagnostic/prognostic potential. These findings provide novel insights into glioma microenvironment regulation and therapeutic target development.

## Supplementary Information

Below is the link to the electronic supplementary material.

Supplementary Material 1

Supplementary Material 2

Supplementary Material 3

Supplementary Material 4

Supplementary Material 5

Supplementary Material 6

Supplementary Material 7

Supplementary Material 8

Supplementary Material 9

Supplementary Material 10

Supplementary Material 11

Supplementary Material 12

Supplementary Material 13

Supplementary Material 14

Supplementary Material 15

Supplementary Material 16

Supplementary Material 17

Supplementary Material 18

Supplementary Material 19

Supplementary Material 20

Supplementary Material 21

Supplementary Material 22

Supplementary Material 23

Supplementary Material 24

Supplementary Material 25

Supplementary Material 26

Supplementary Material 27

Supplementary Material 28

Supplementary Material 29

Supplementary Material 30

Supplementary Material 31

Supplementary Material 32

Supplementary Material 33

## Data Availability

All data generated or analysed during this study are included in this published article and its supplementary information files.

## Abbreviations

CSTA: Cystatin A
AAPM: Amino acid point-mutant
AUC: Area under the curve
ChIP-PCR: Chromatin immunoprecipitation-PCR
CSF: Cerebrospinal fluid
DEGs: Differentially expressed genes
ELISA: Enzyme-linked immunosorbent assay
GAMs: Glioma-associated macrophage/microglia
GBM: Glioblastoma
IF: Immunofluorescence
IP: Immunoprecipitation
LASSO: Least absolute shrinkage and selection operator
qPCR: Quantitative polymerase chain reaction
ROC: Receiver operating characteristic
RRA: Robust rank aggregation
scRNA-seq: single-cell RNA sequencing
ssGSEA: Single-sample gene set enrichment analysis
TME: Tumor microenvironment
UMAP: Uniform manifold approximation and projection
WB: Western blotting
BBB: Blood-brain barrier
