# Supplementary figures and images for: M2-like GAMs secreting CSTA drive glioblastoma progression via the ITGB4-TGFB1 feedback axis
Source: J Transl Med. 2026 Mar 14;24:567. doi: 10.1186/s12967-026-08009-0 (PMC13101109; doi:10.1186/s12967-026-08009-0)

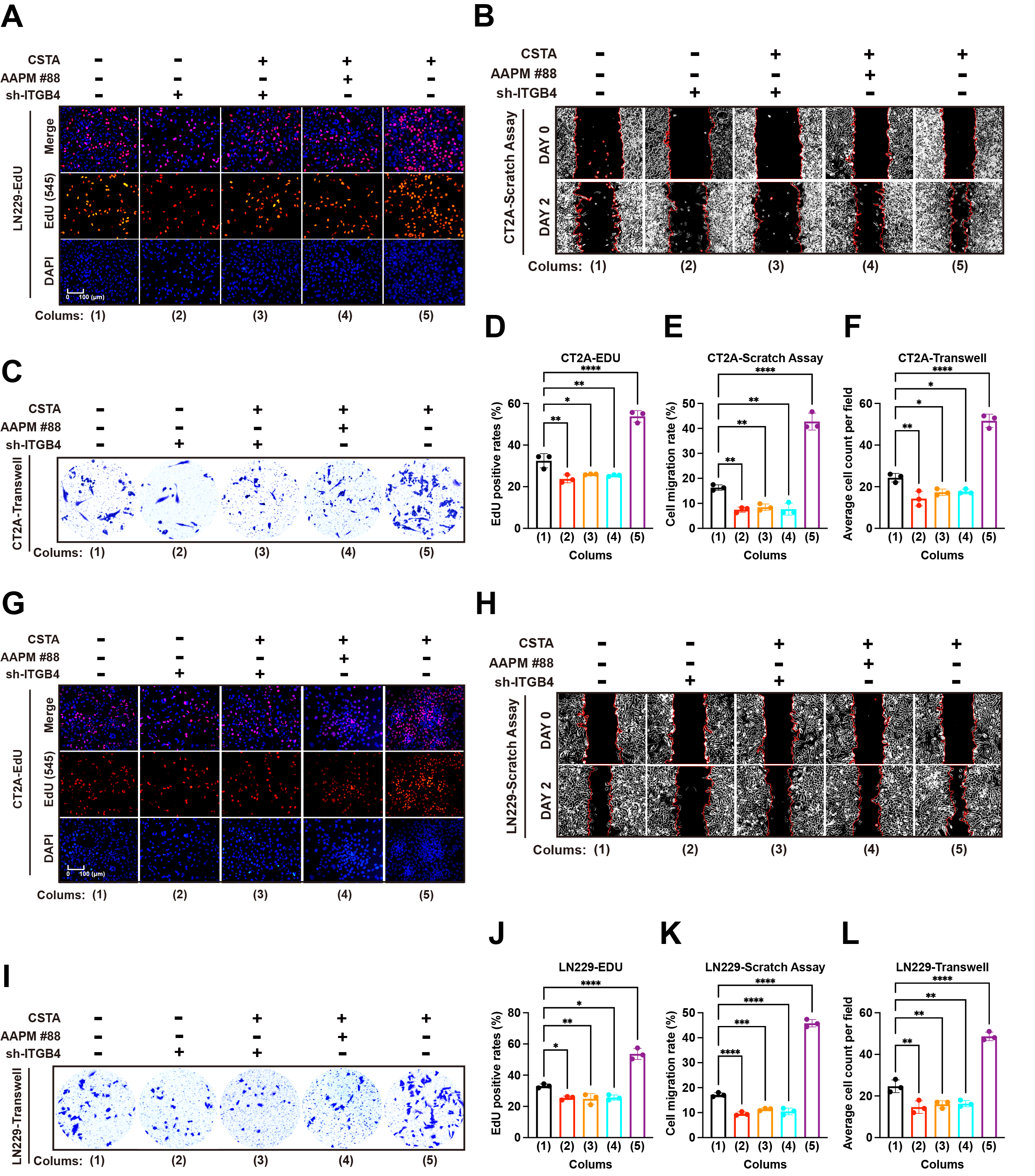

Supplement: Supplementary file 7 — Supplementary Material 7 [file 12967_2026_8009_MOESM7_ESM.tif]

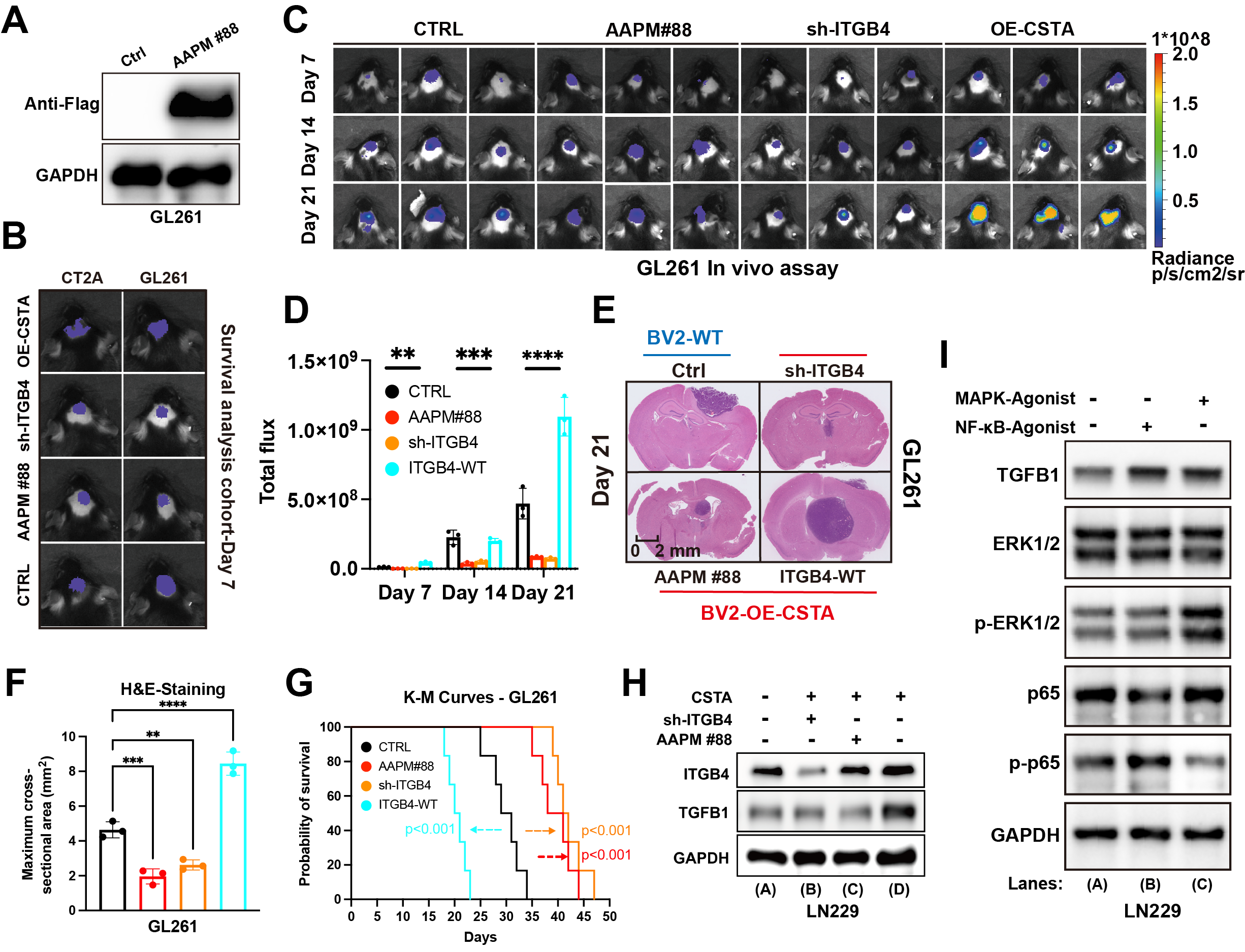

Supplement: Supplementary file 8 — Supplementary Material 8 [file 12967_2026_8009_MOESM8_ESM.tif]

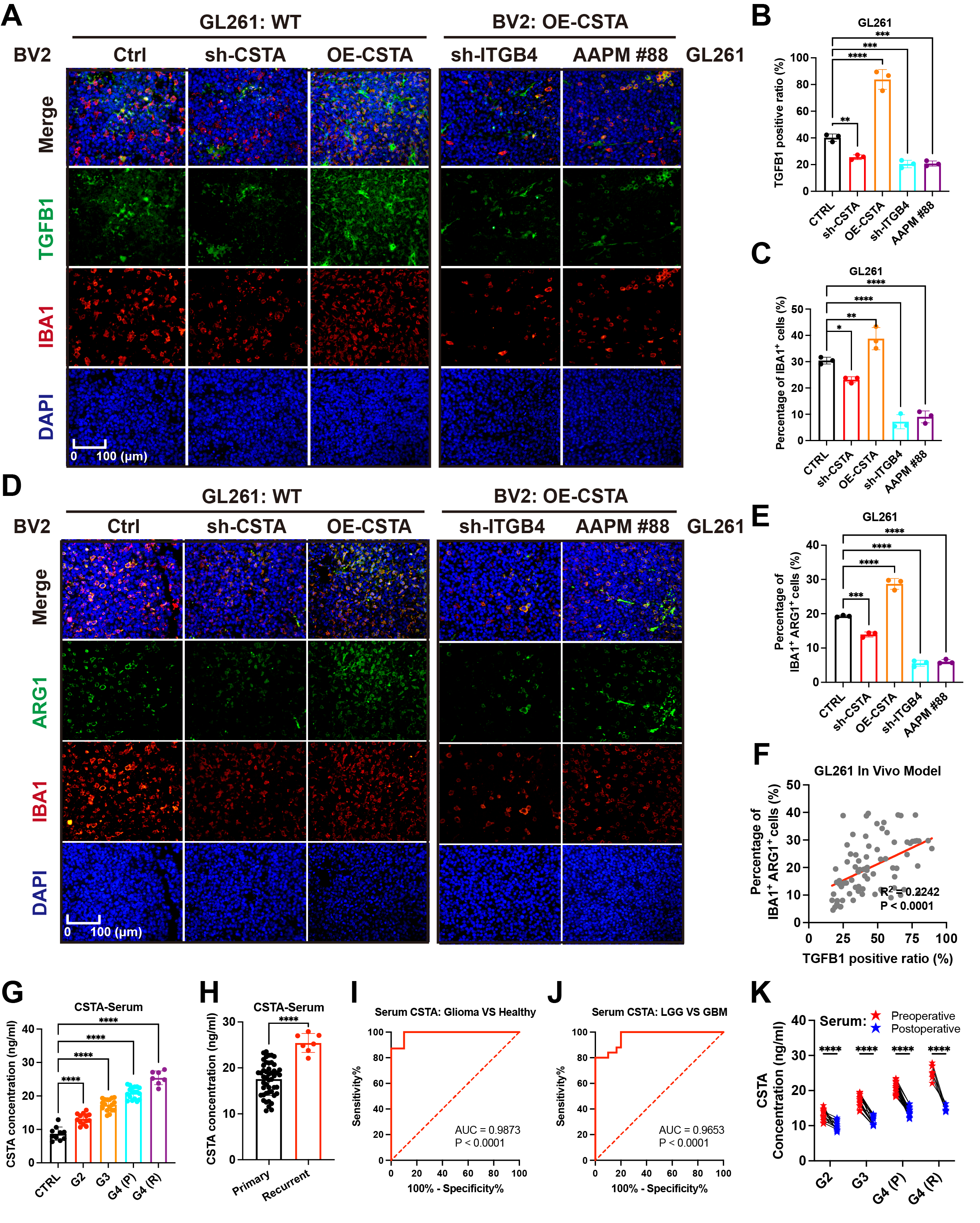

Supplement: Supplementary file 9 — Supplementary Material 9 [file 12967_2026_8009_MOESM9_ESM.tif]

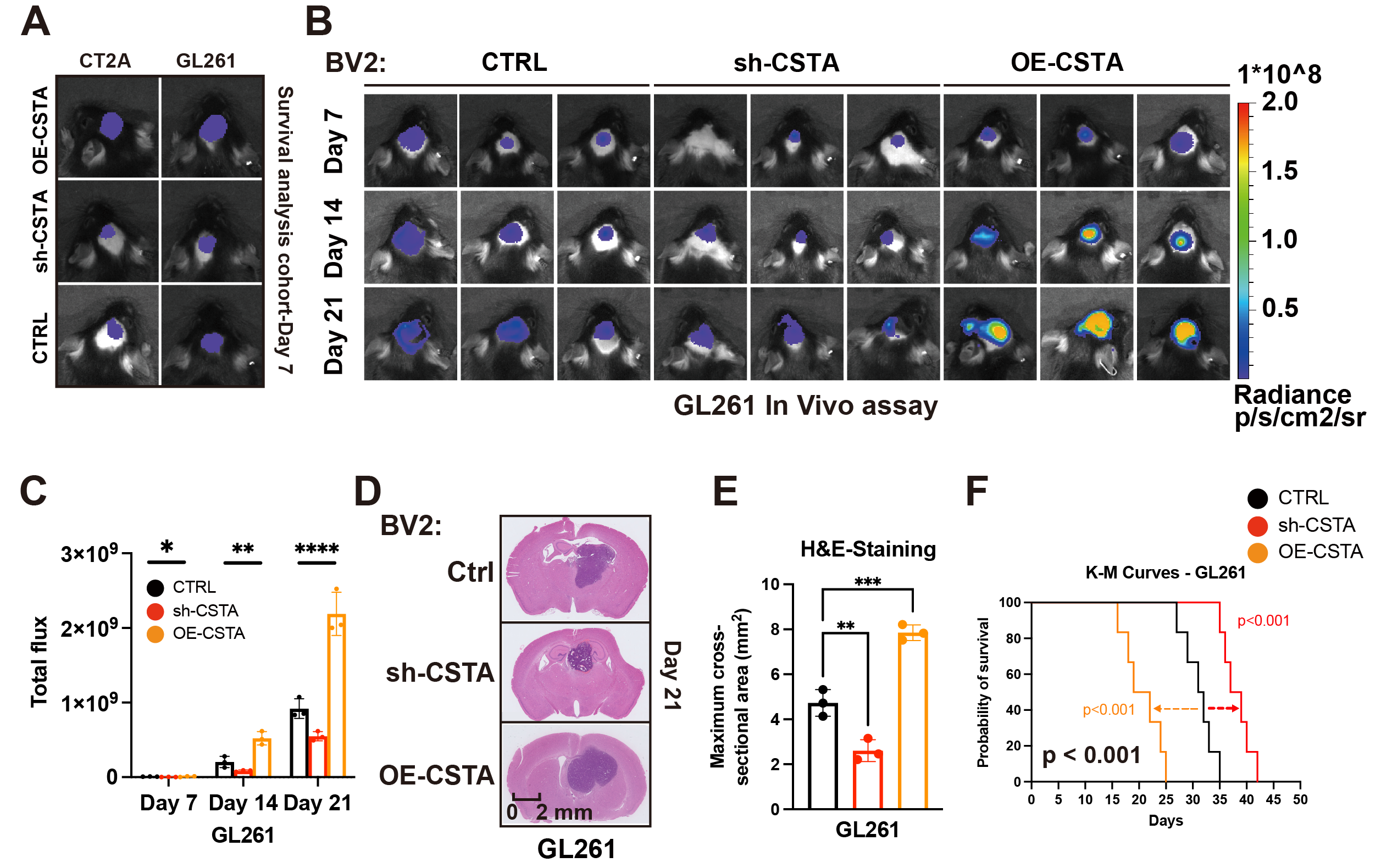

Supplement: Supplementary file 10 — Supplementary Material 10 [file 12967_2026_8009_MOESM10_ESM.tif]

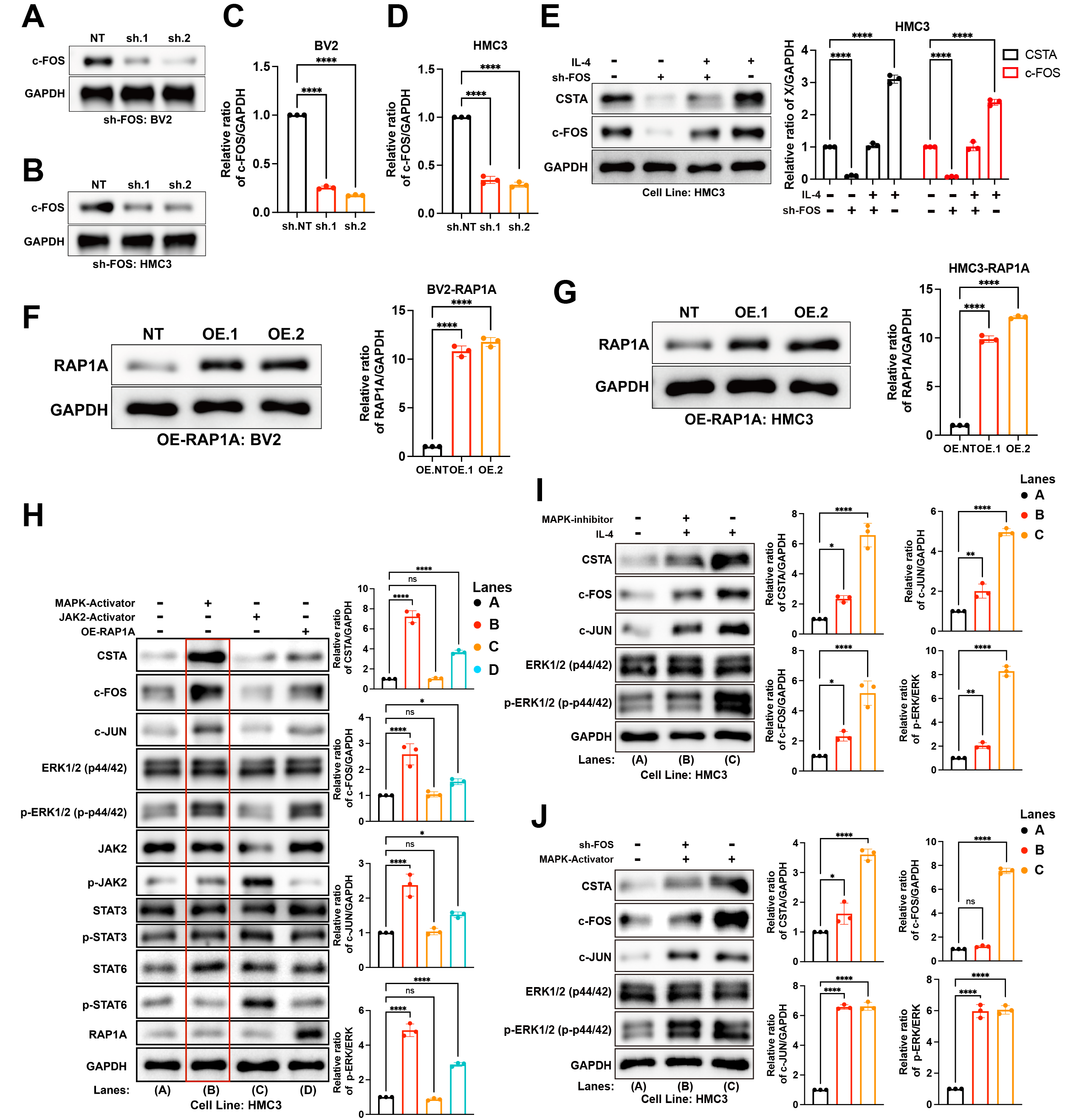

Supplement: Supplementary file 11 — Supplementary Material 11 [file 12967_2026_8009_MOESM11_ESM.tif]

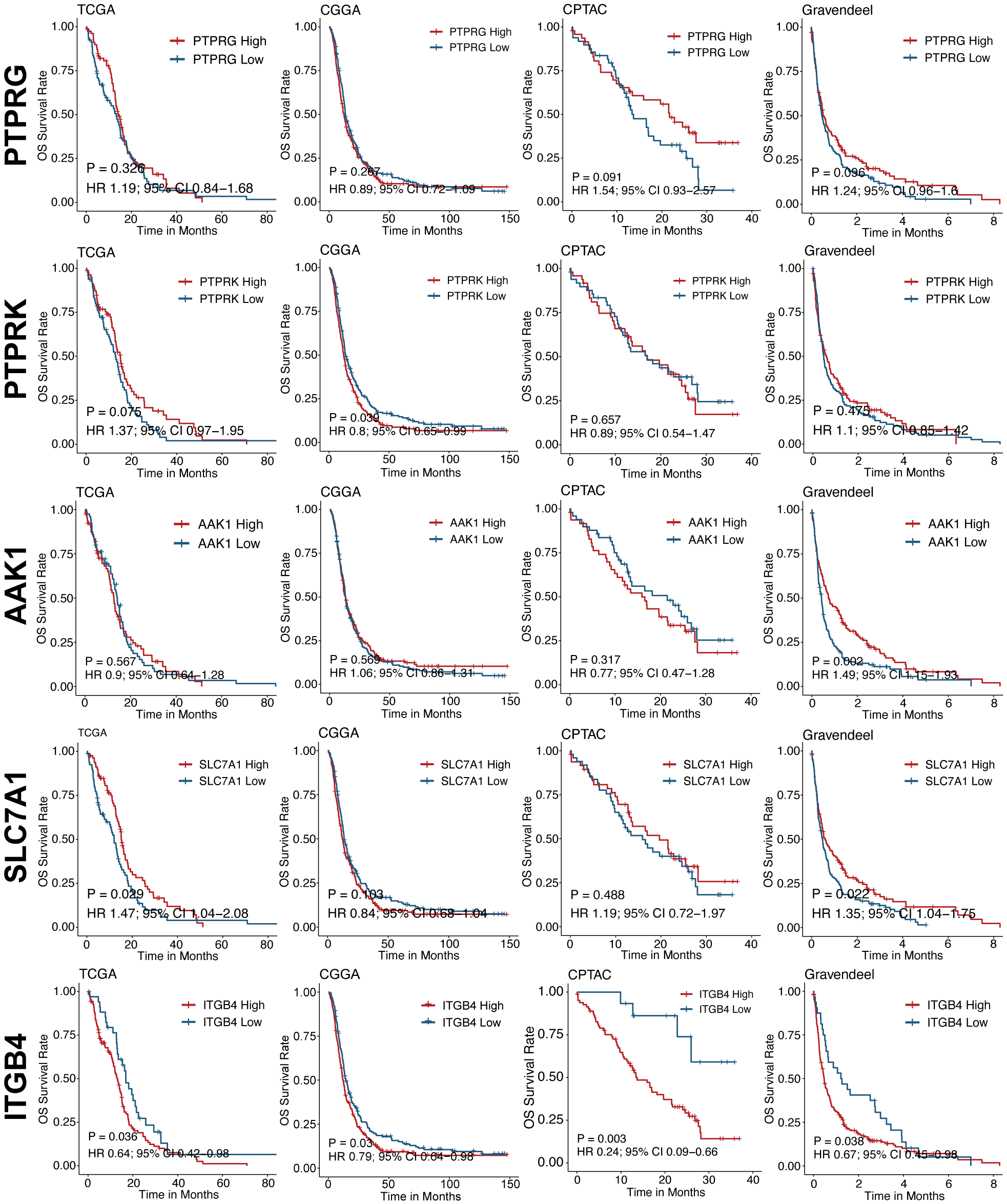

Supplement: Supplementary file 12 — Supplementary Material 12 [file 12967_2026_8009_MOESM12_ESM.tif]

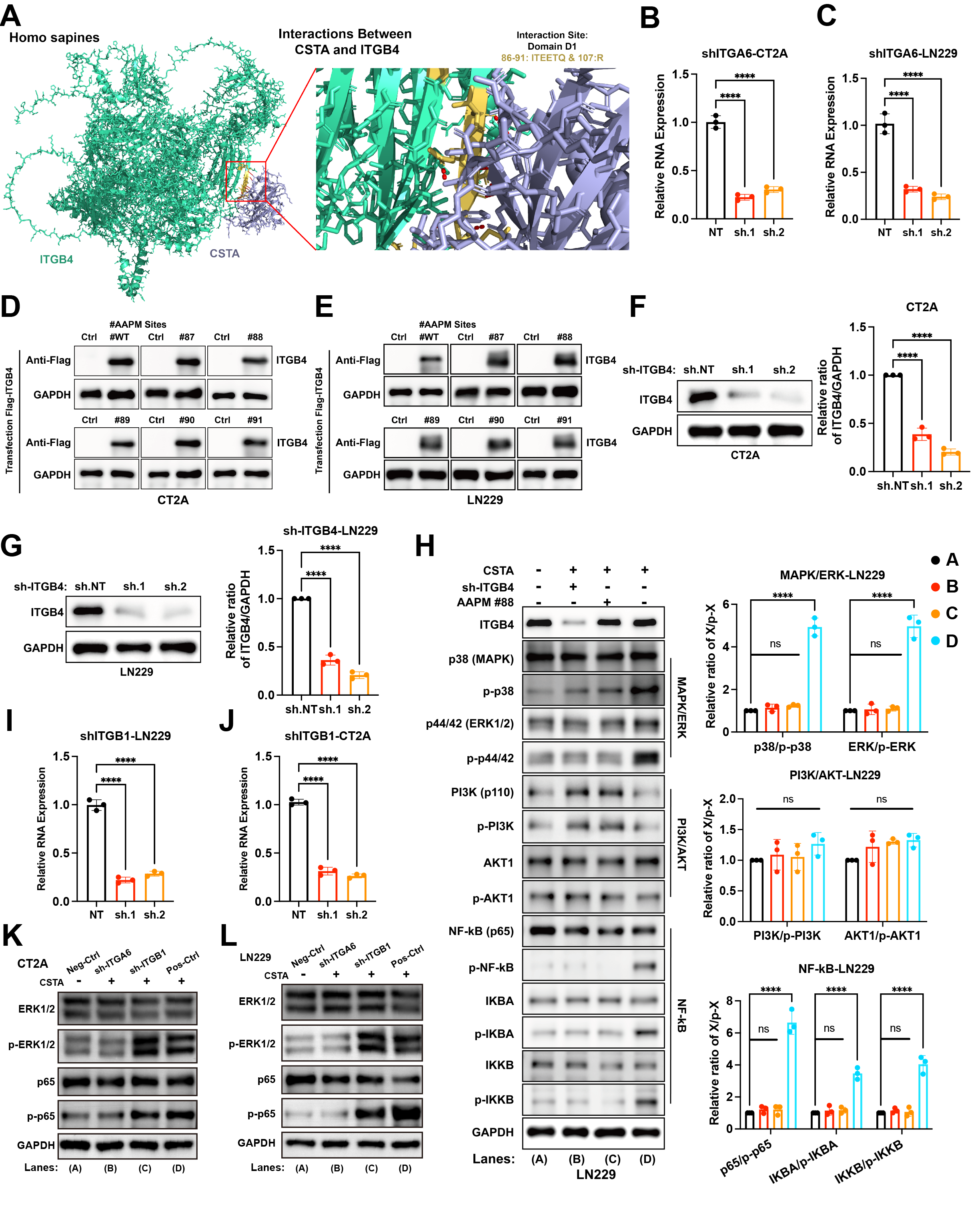

Supplement: Supplementary file 13 — Supplementary Material 13 [file 12967_2026_8009_MOESM13_ESM.tif]

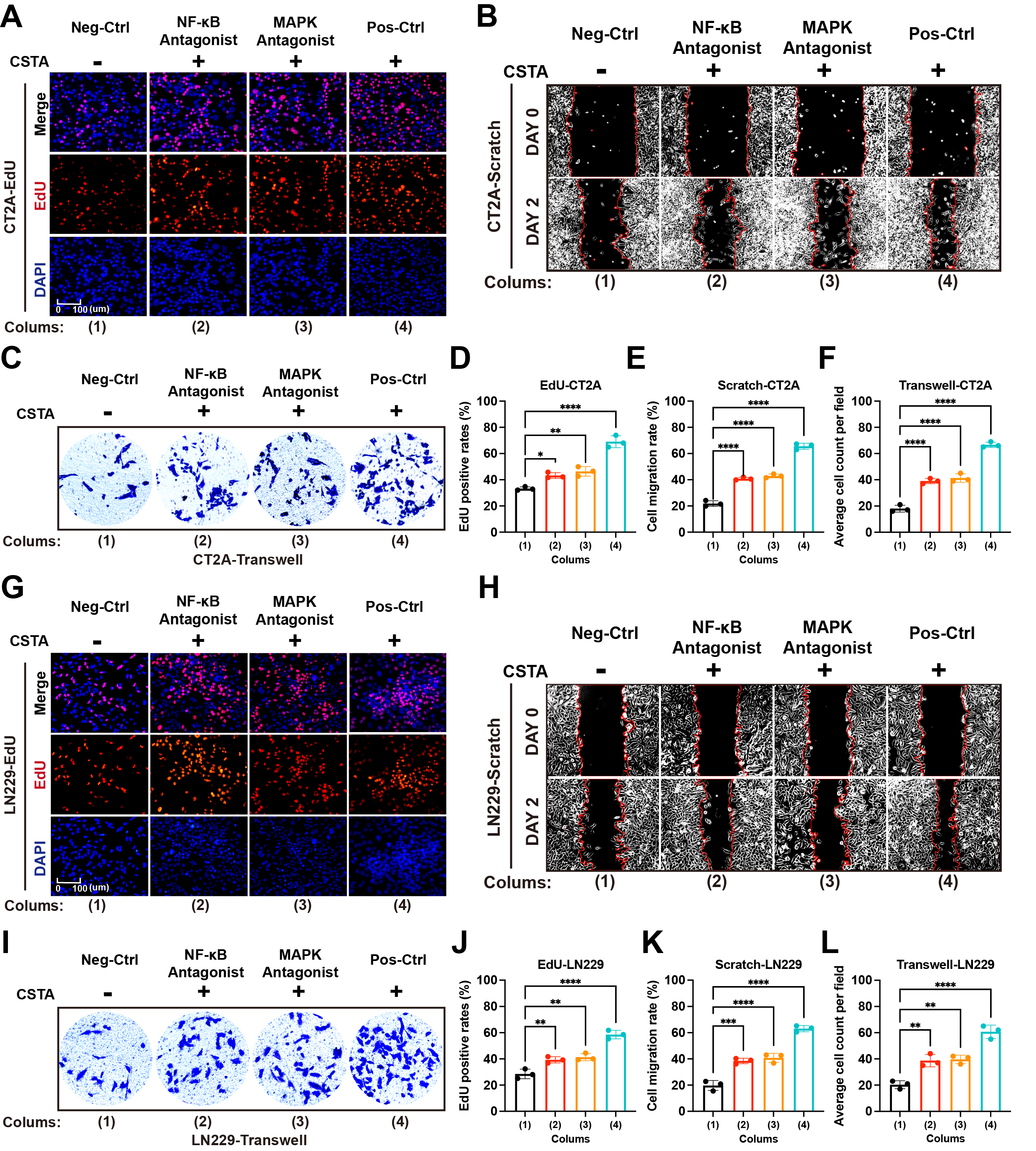

Supplement: Supplementary file 14 — Supplementary Material 14 [file 12967_2026_8009_MOESM14_ESM.tif]

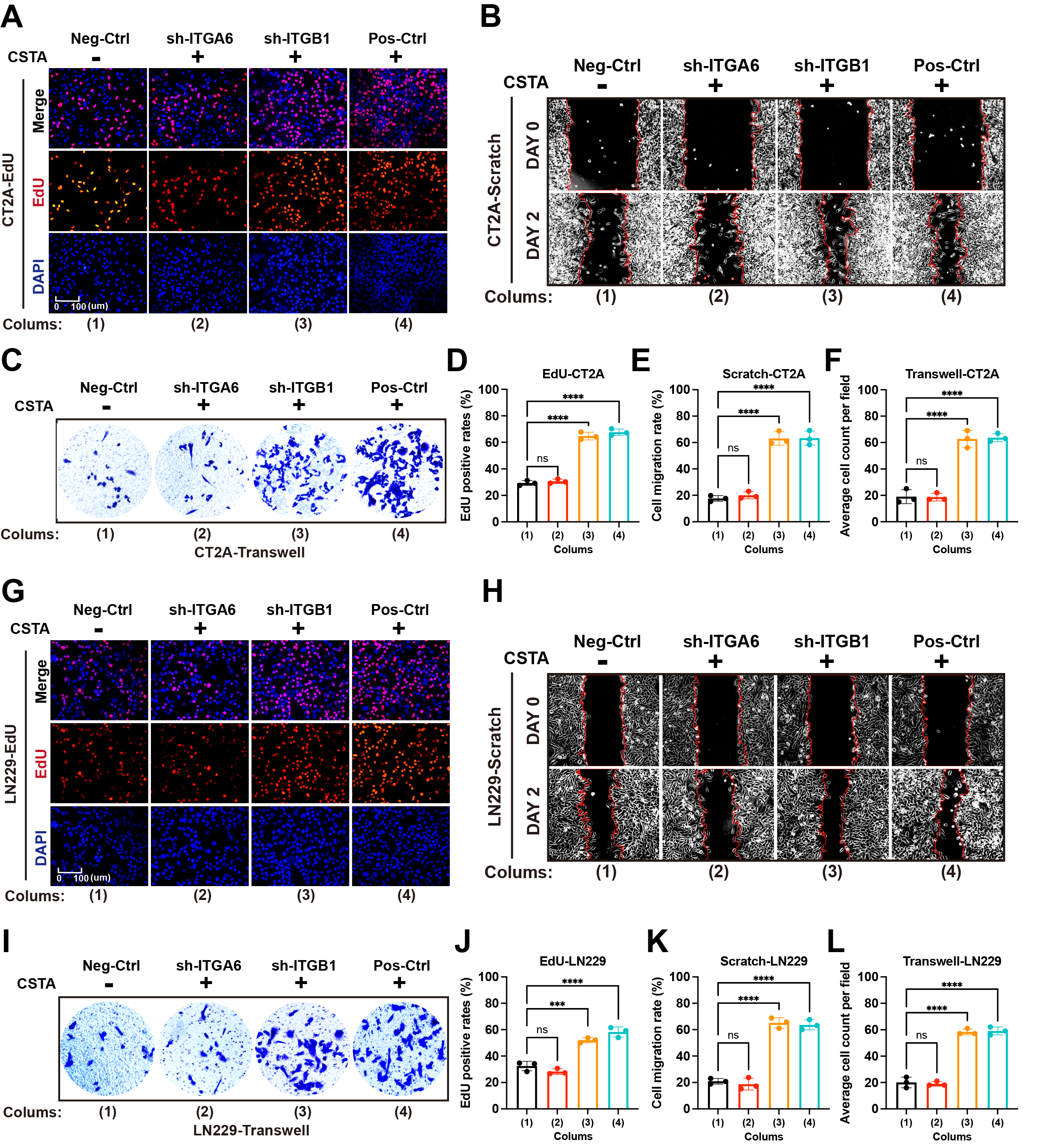

Supplement: Supplementary file 15 — Supplementary Material 15 [file 12967_2026_8009_MOESM15_ESM.tif]

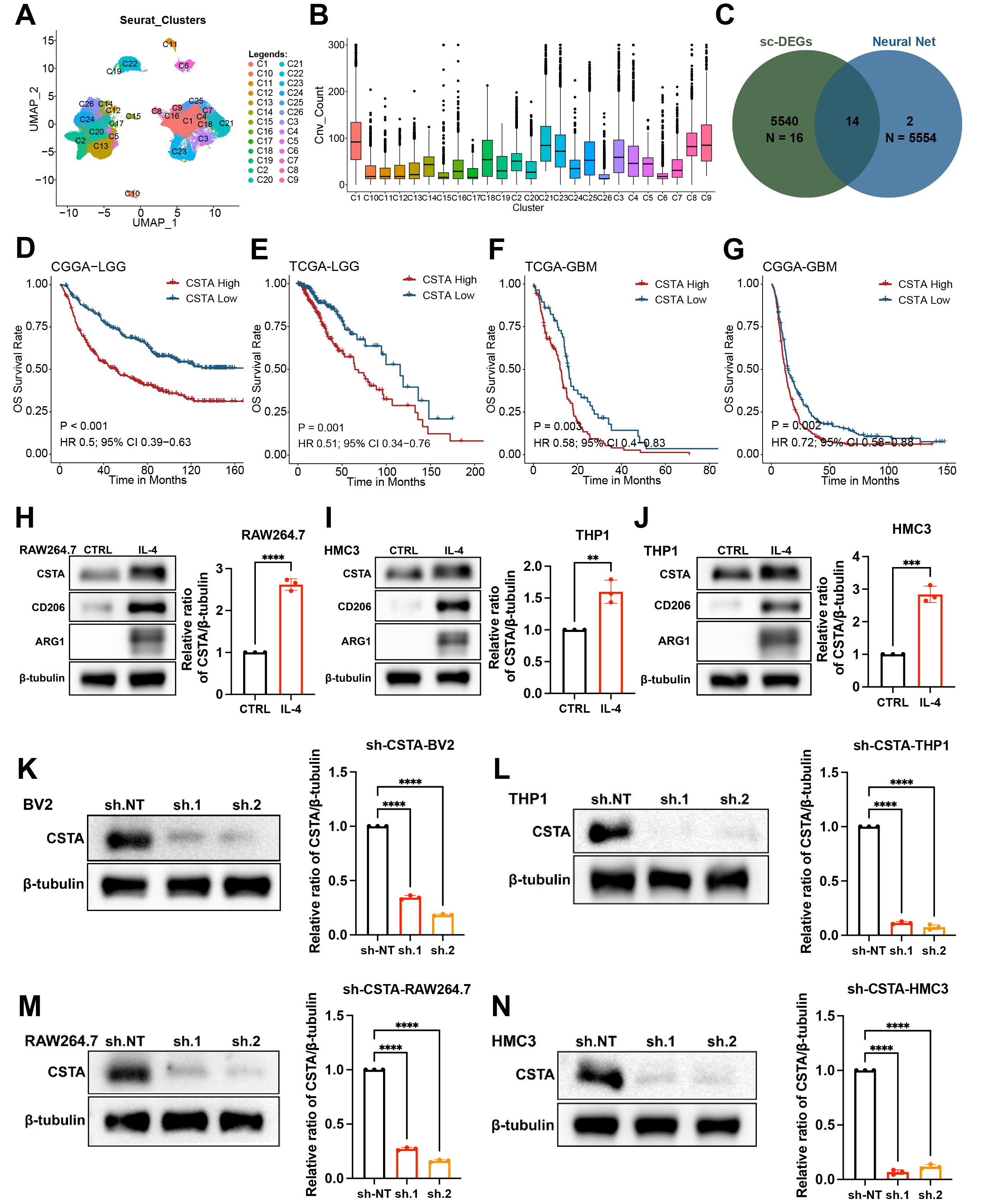

Supplement: Supplementary file 16 — Supplementary Material 16 [file 12967_2026_8009_MOESM16_ESM.tif]

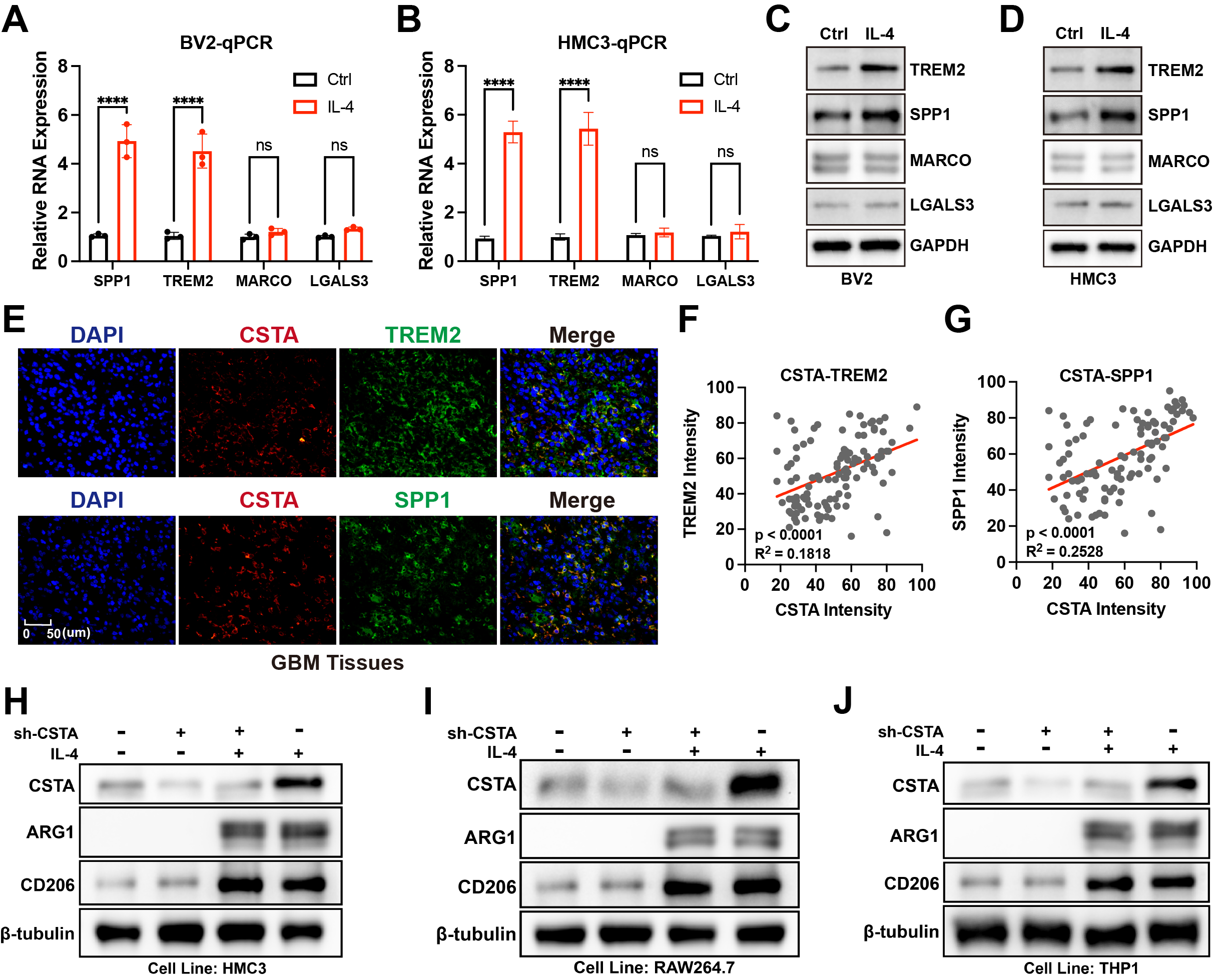

Supplement: Supplementary file 17 — Supplementary Material 17 [file 12967_2026_8009_MOESM17_ESM.tif]

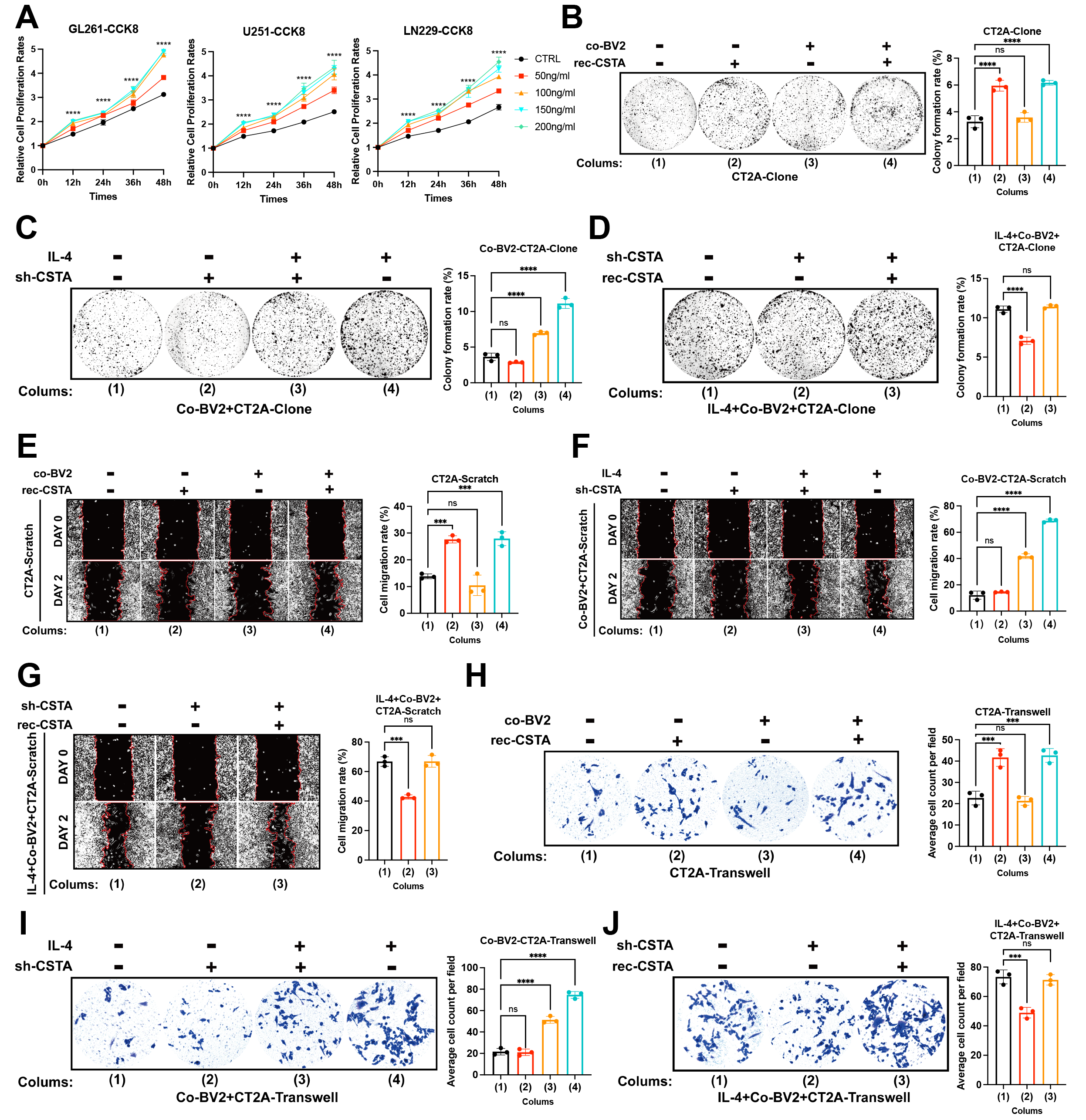

Supplement: Supplementary file 18 — Supplementary Material 18 [file 12967_2026_8009_MOESM18_ESM.tif]

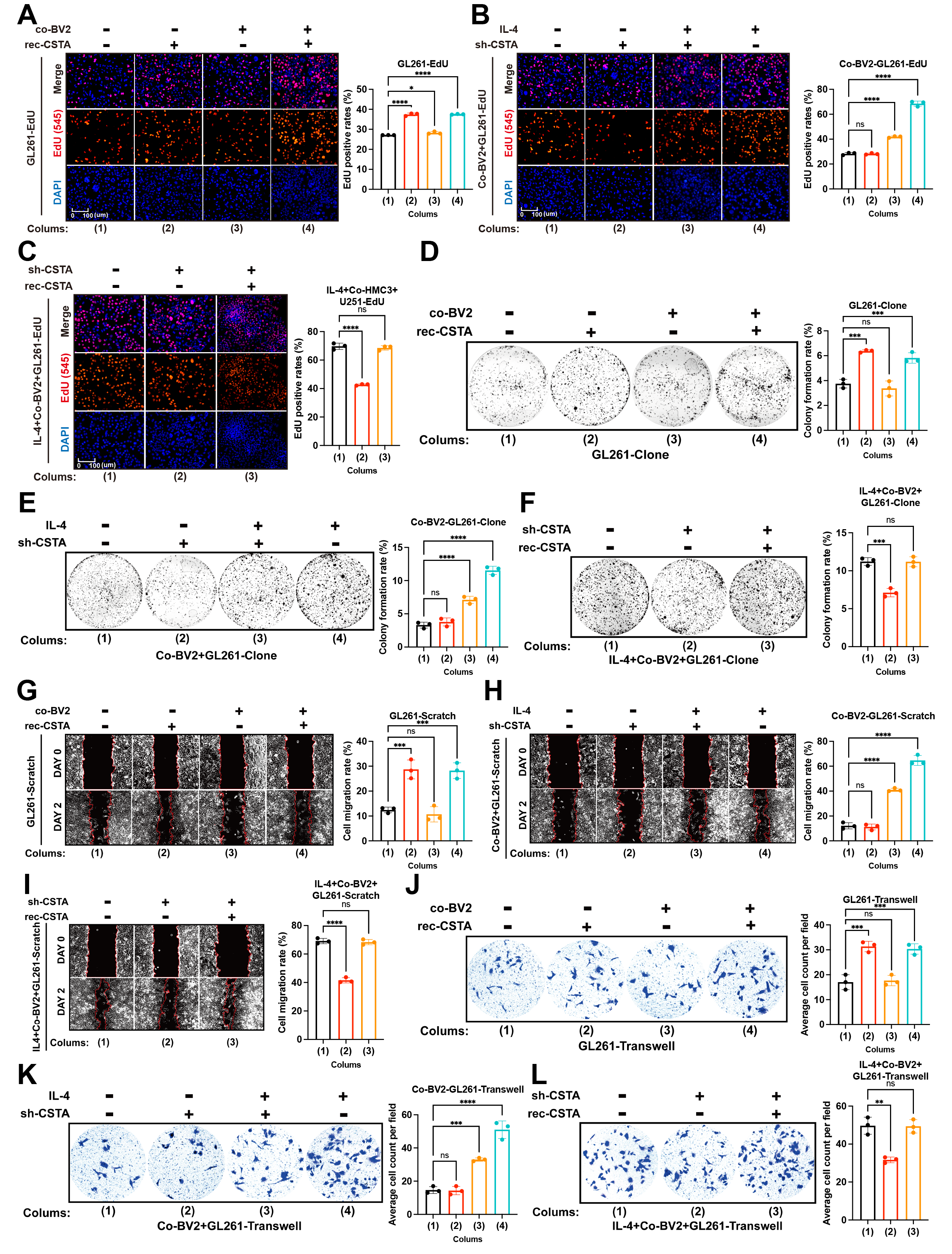

Supplement: Supplementary file 19 — Supplementary Material 19 [file 12967_2026_8009_MOESM19_ESM.tif]

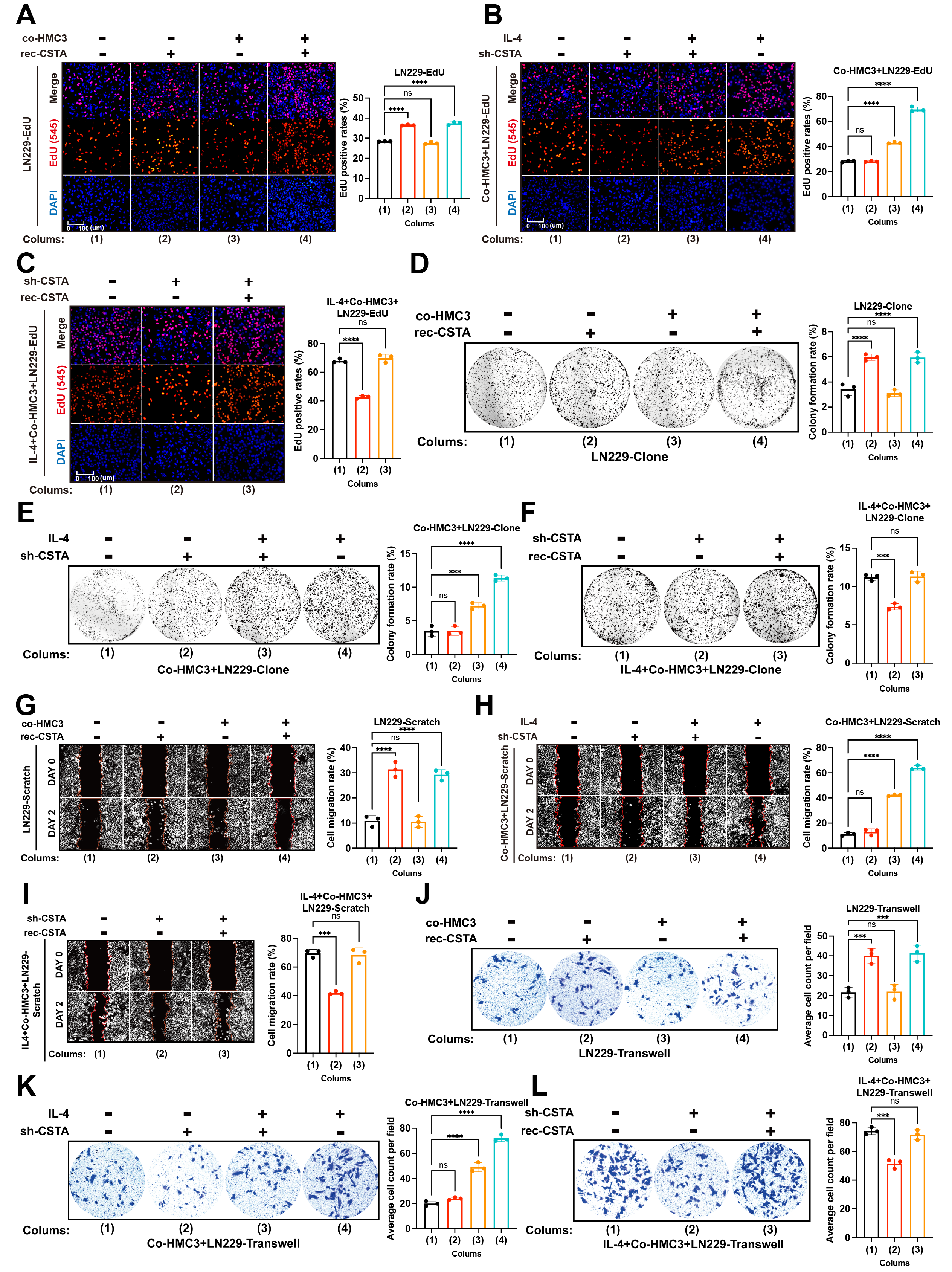

Supplement: Supplementary file 20 — Supplementary Material 20 [file 12967_2026_8009_MOESM20_ESM.tif]

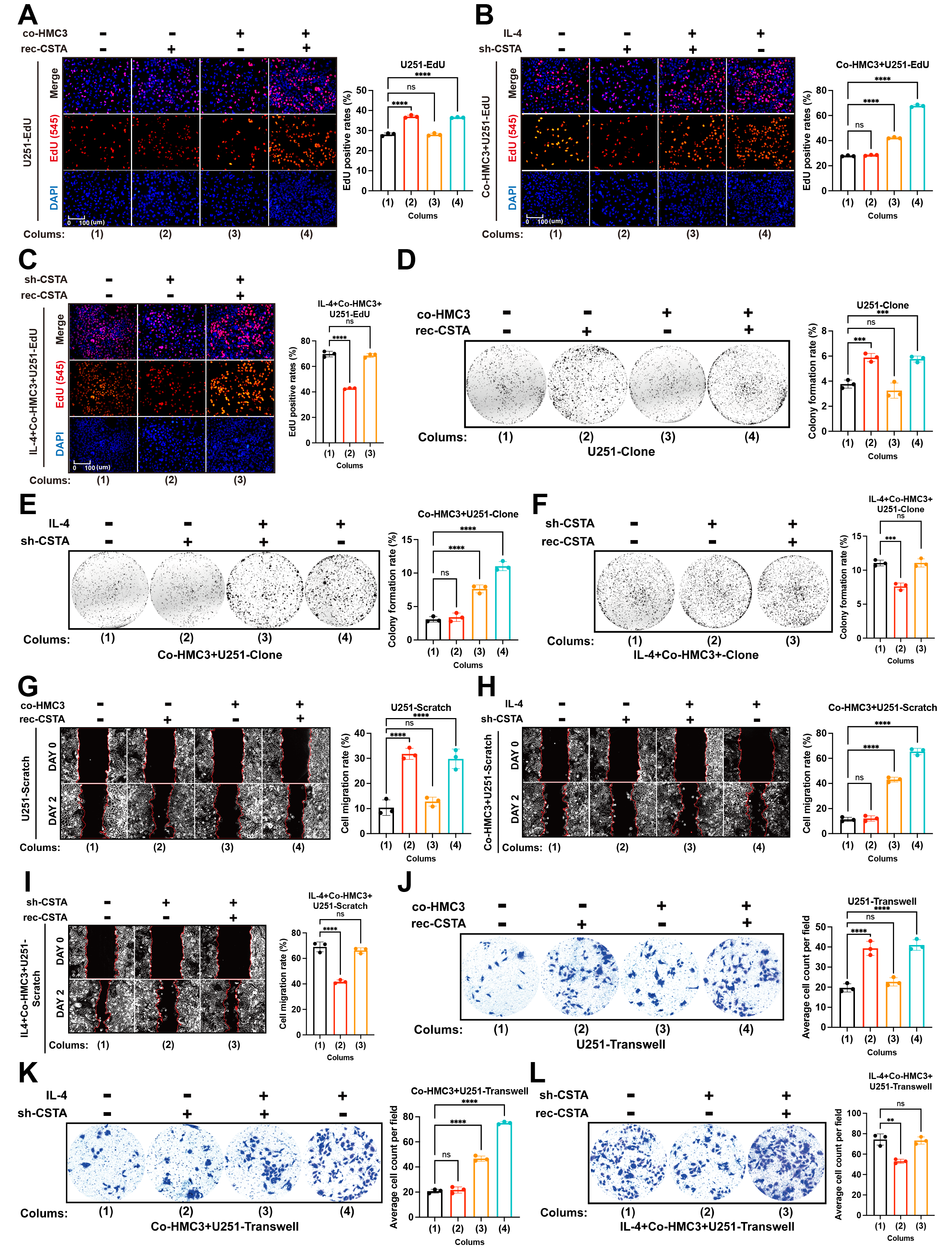

Supplement: Supplementary file 21 — Supplementary Material 21 [file 12967_2026_8009_MOESM21_ESM.tif]

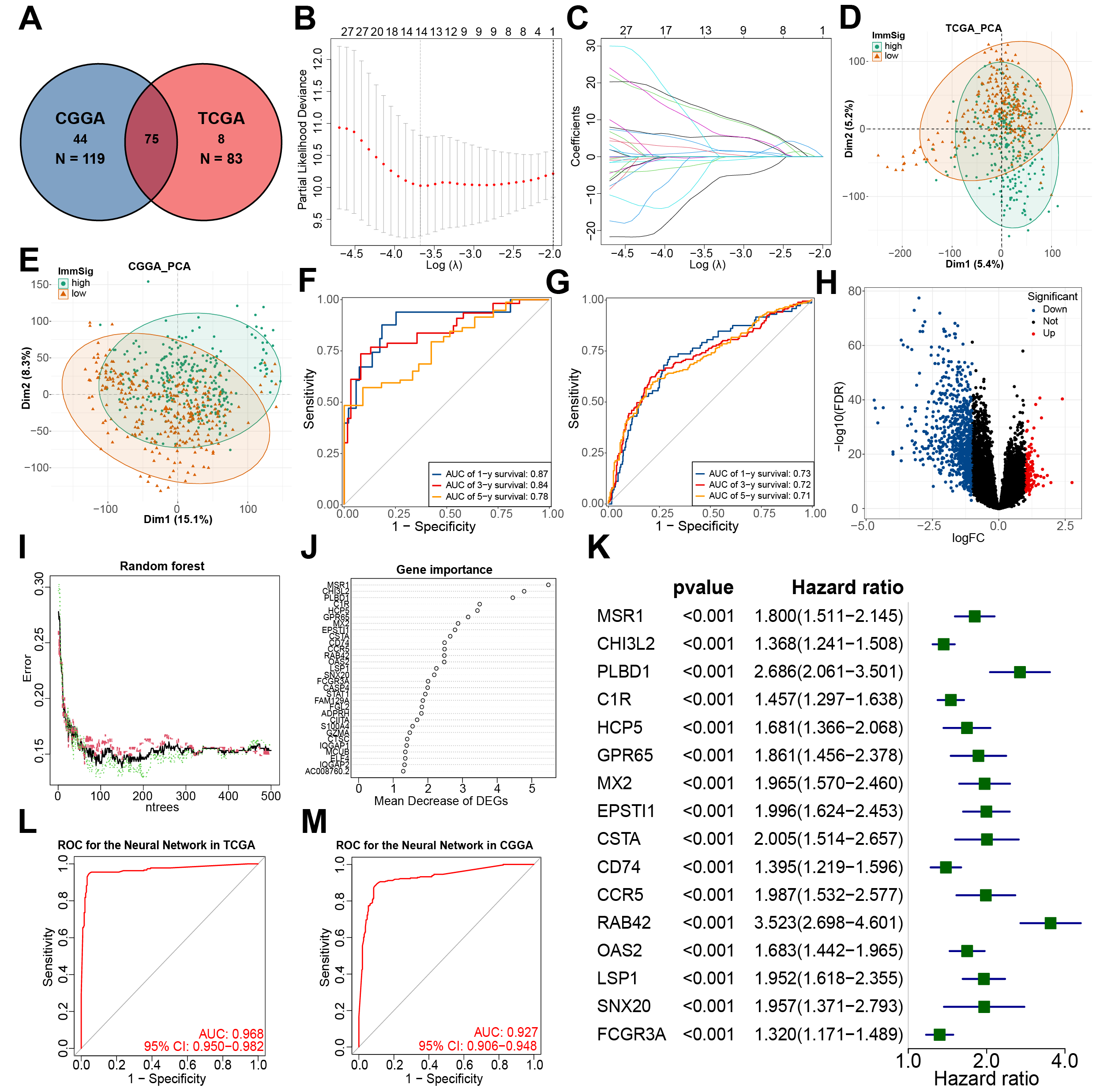

Supplement: Supplementary file 27 — Supplementary Material 27 [file 12967_2026_8009_MOESM27_ESM.tif]
