# Supplementary material for: M2-like GAMs secreting CSTA drive glioblastoma progression via the ITGB4-TGFB1 feedback axis
Source: J Transl Med. 2026 Mar 14;24:567. doi: 10.1186/s12967-026-08009-0 (PMC13101109; doi:10.1186/s12967-026-08009-0)

Figure 1F

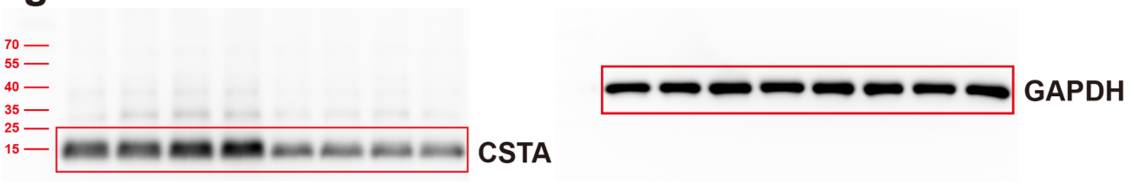

Figure 1H

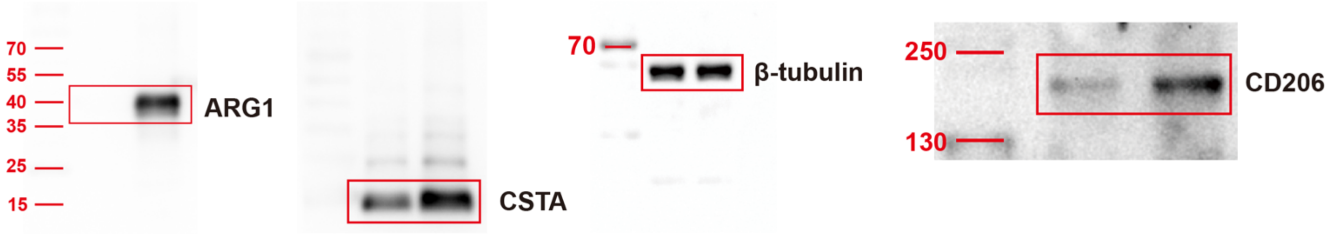

Figure S2H

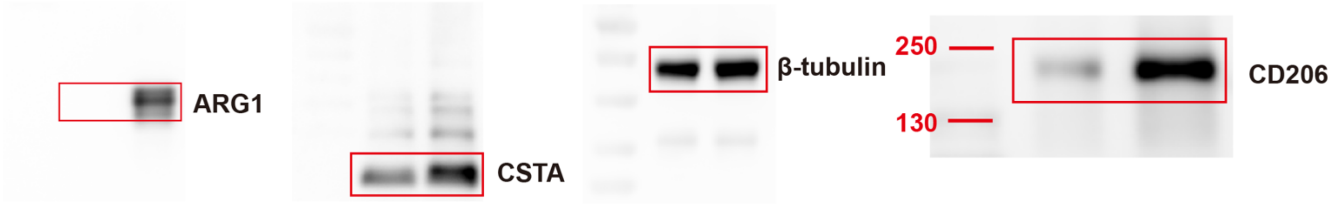

Figure S2I

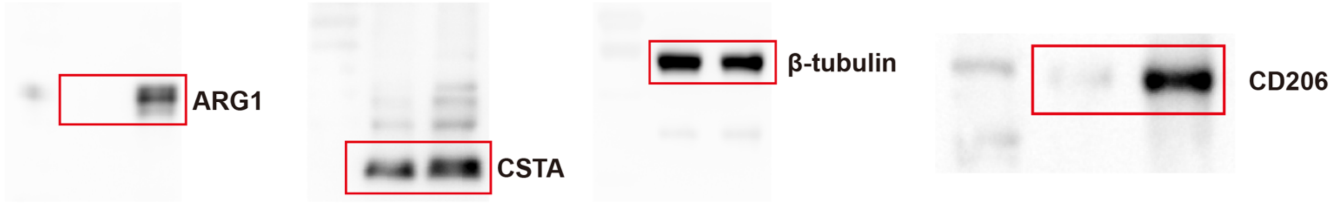

Figure S2J

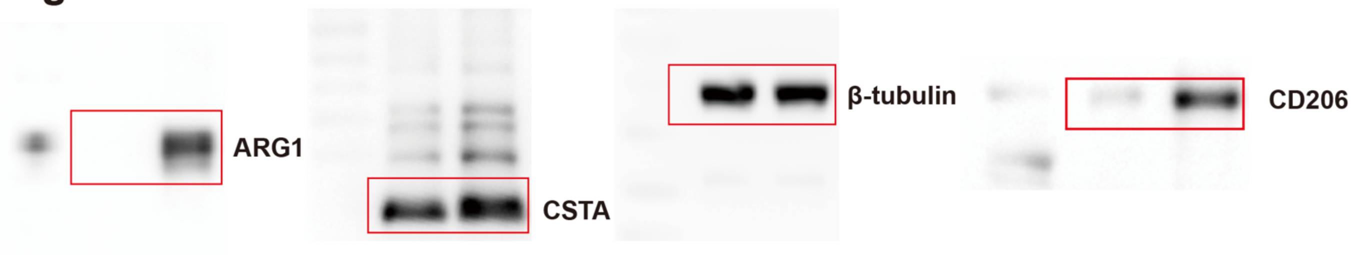

Figure S2K

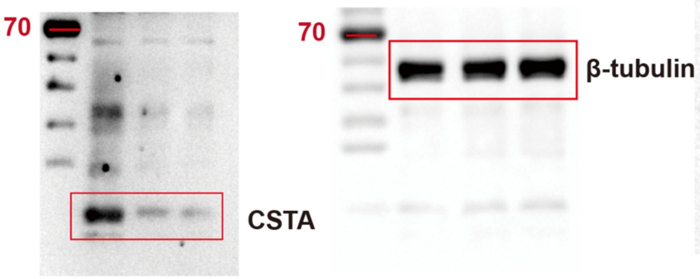

Figure S2L

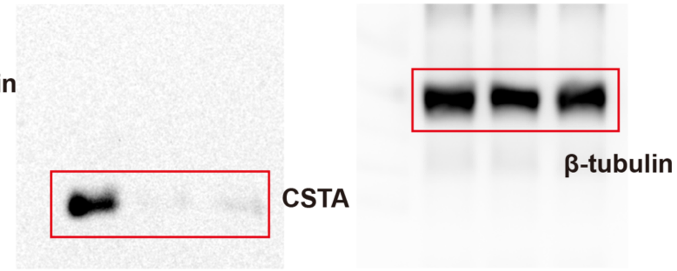

Figure S2M

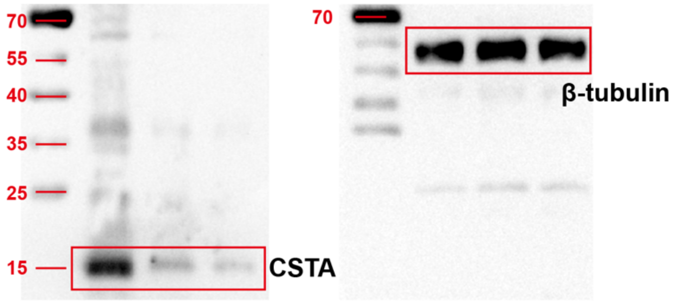

Figure S2N

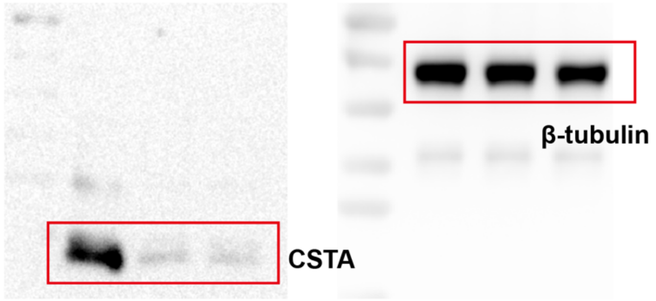

Figure 1M

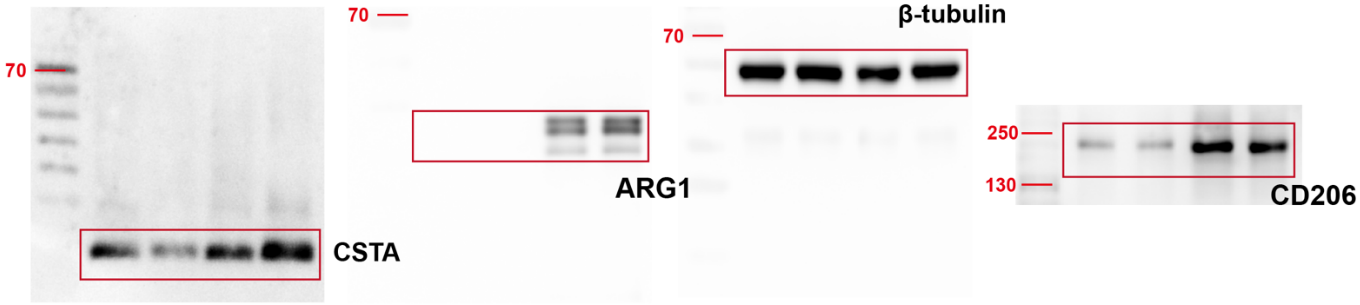

Figure S3H

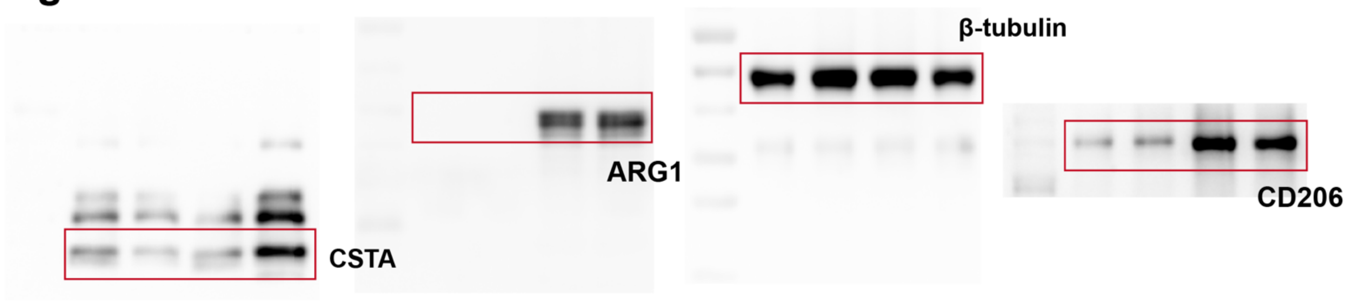

Figure S3I

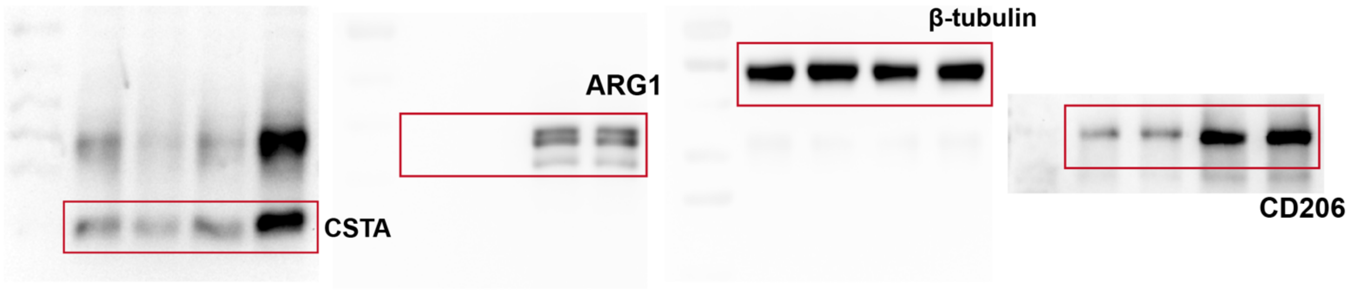

Figure S3J

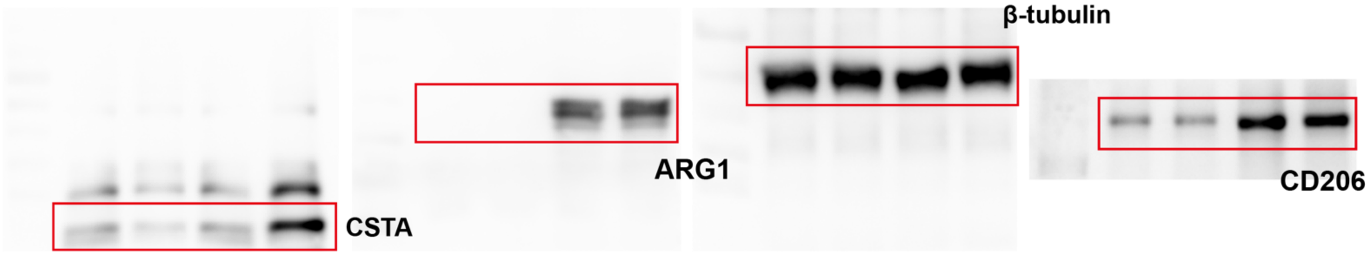

Figure S9A

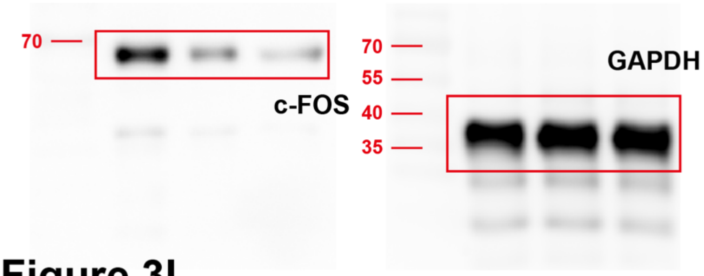

Figure S9B

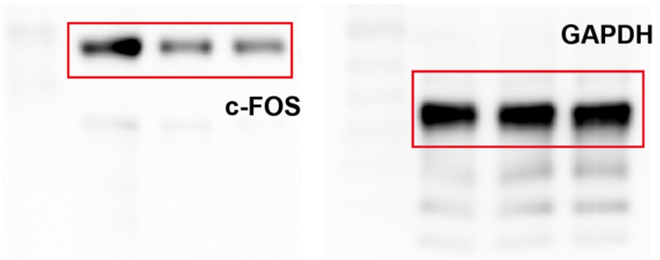

Figure 3I

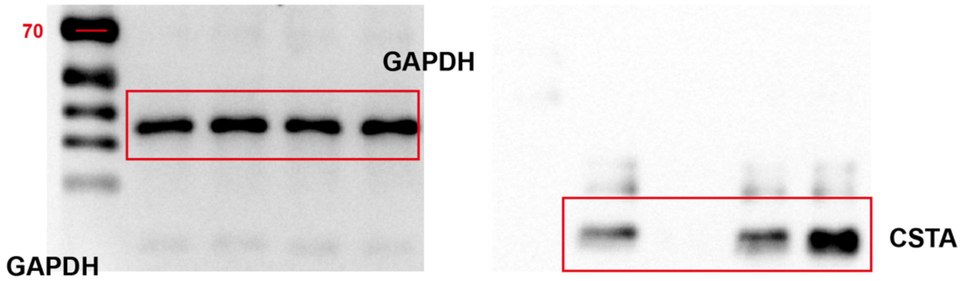

Figure S9E

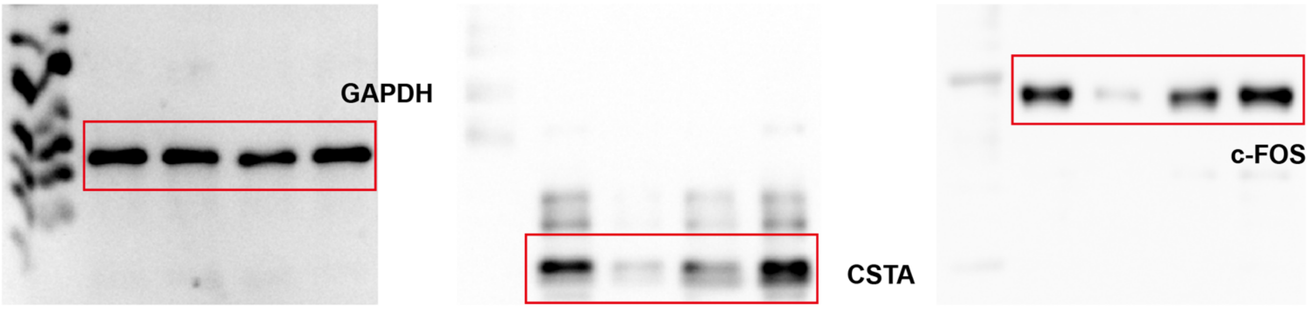

Figure S9F

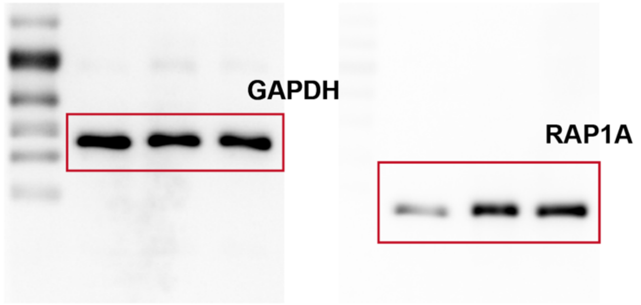

Figure S9G

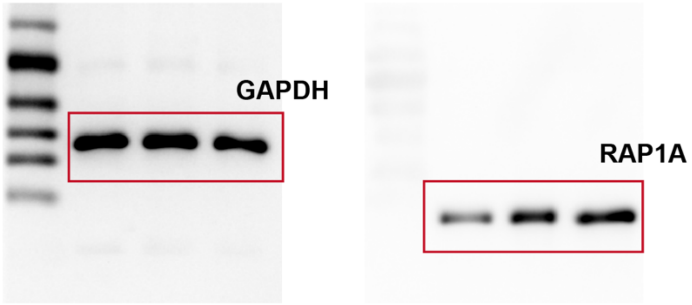

Figure 3J

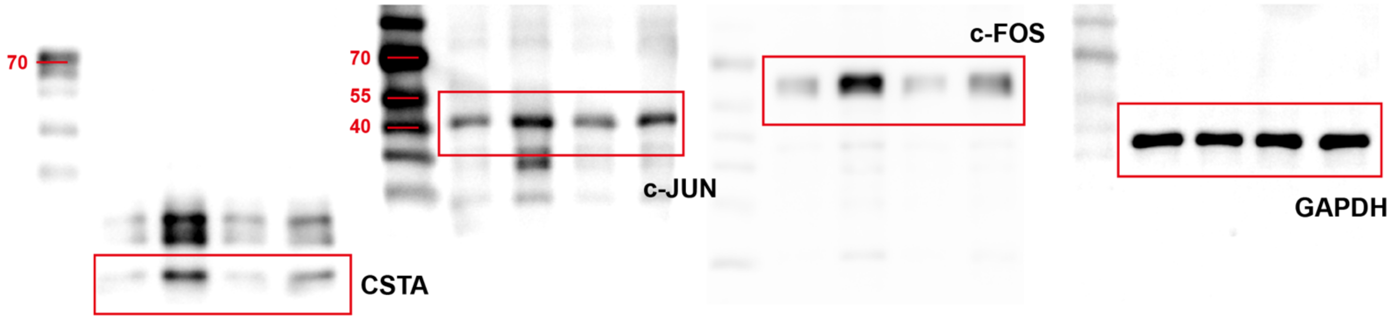

Figure 3J

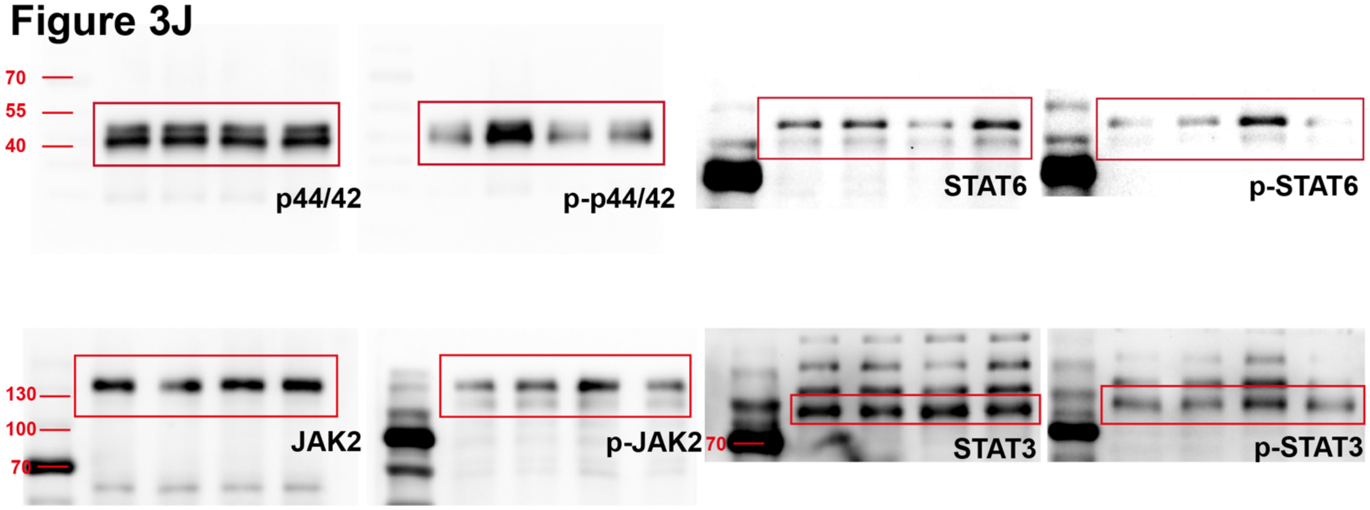

Figure S9H

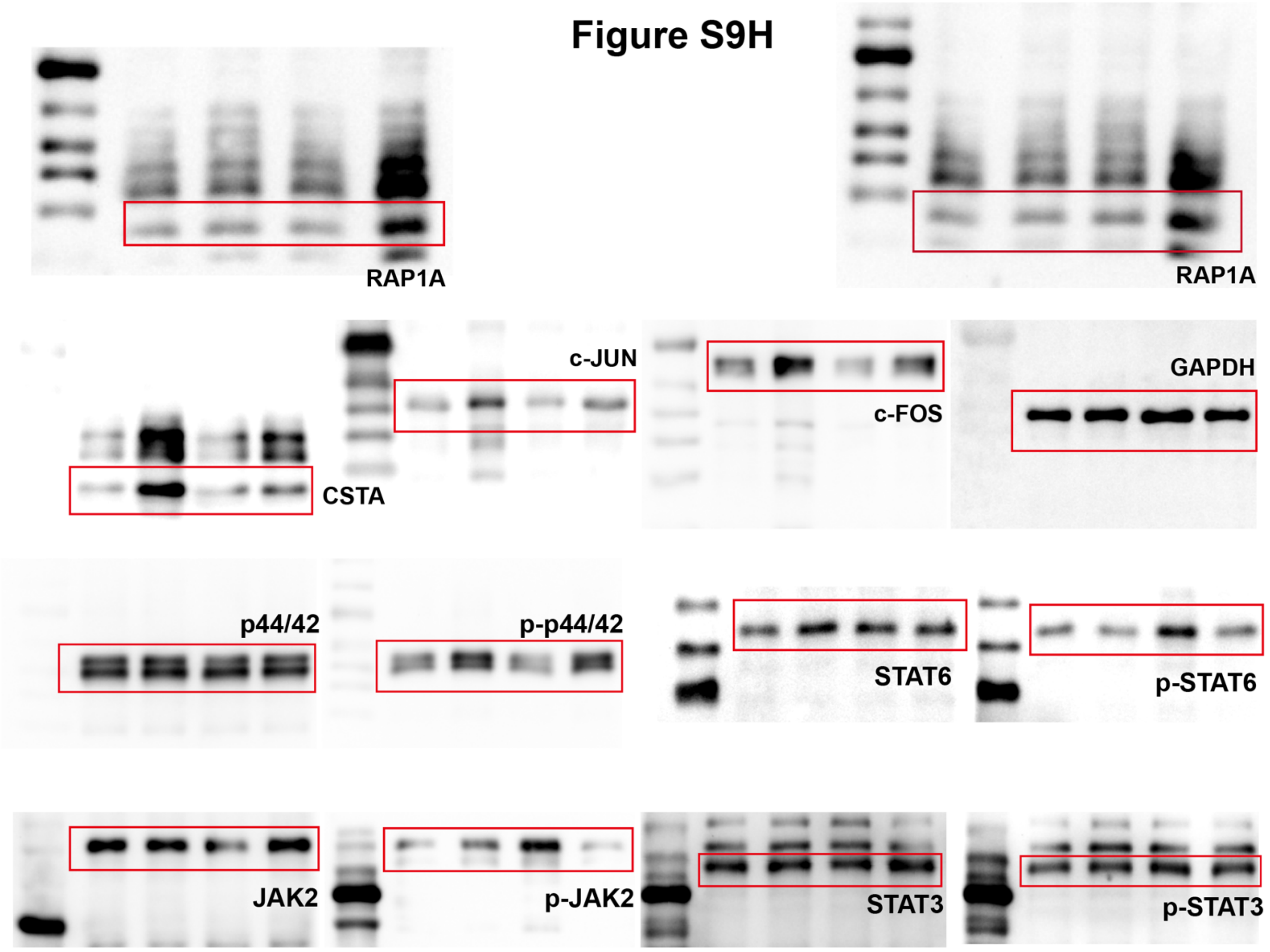

Figure 3K

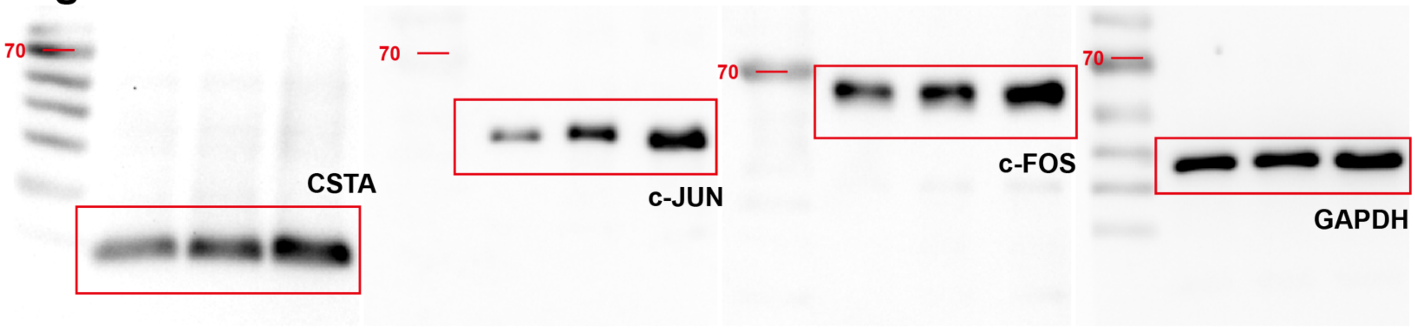

Figure S9I

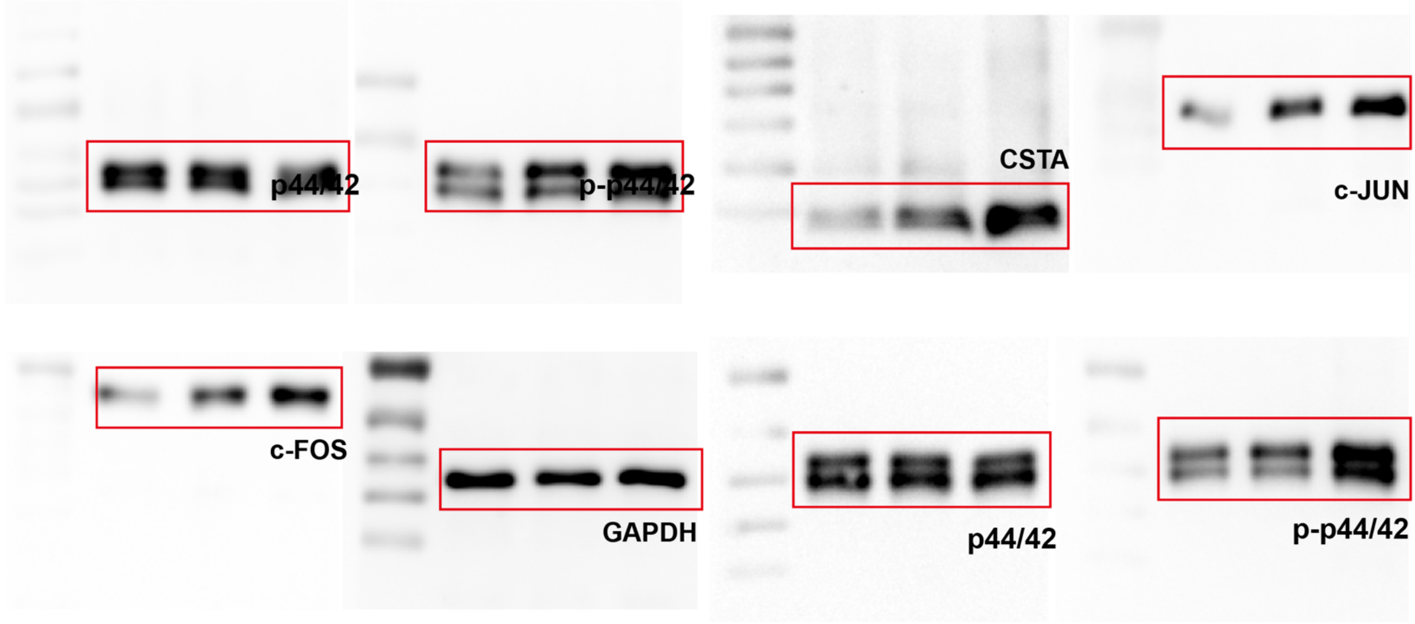

Figure 3L

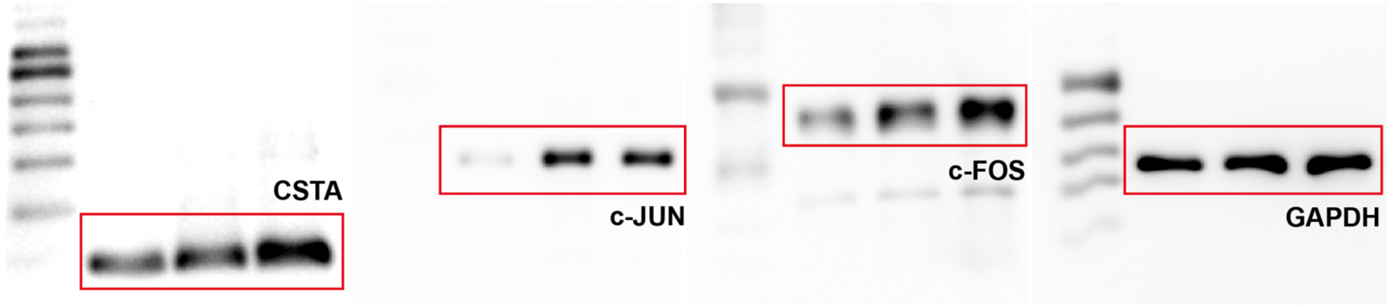

Figure 3L

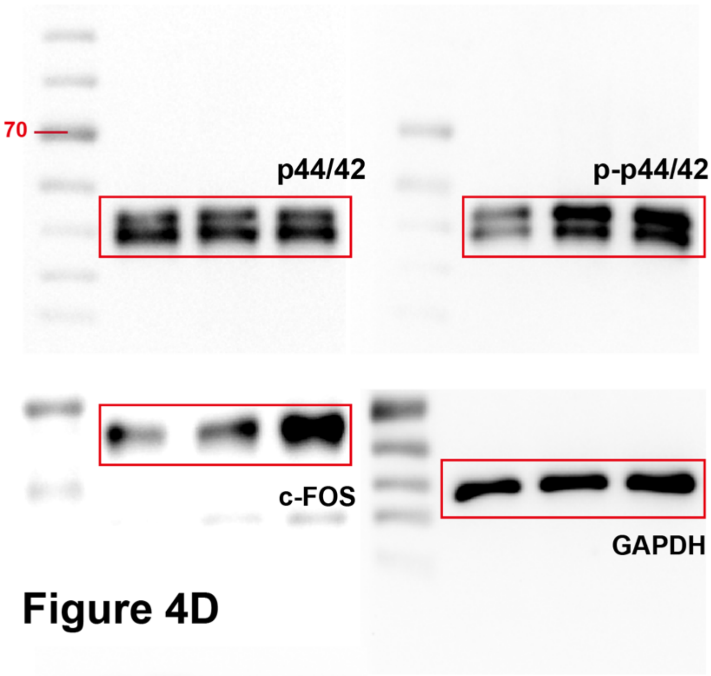

Figure S9J

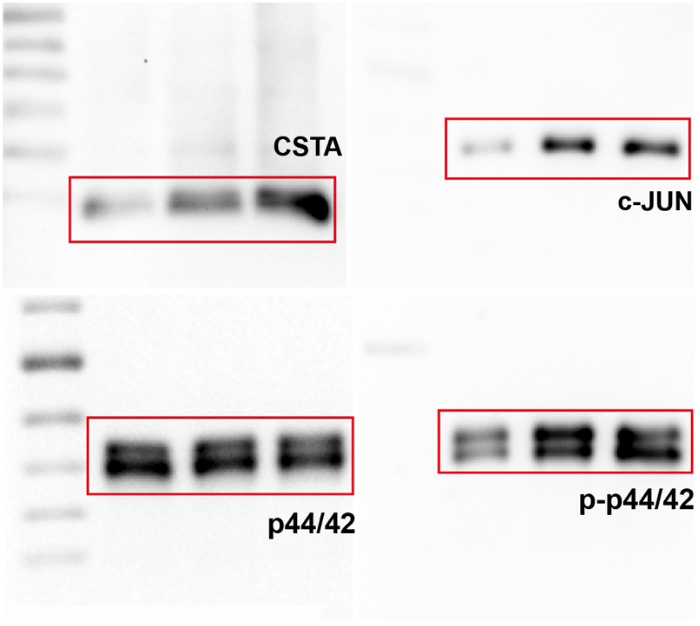

Figure 4D

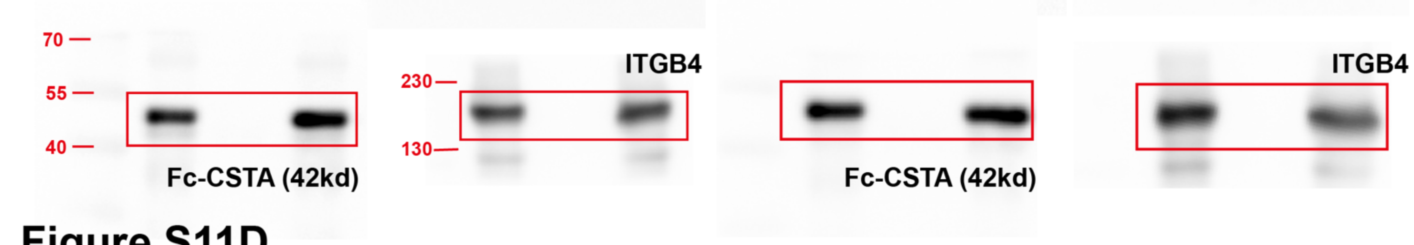

Figure S11D

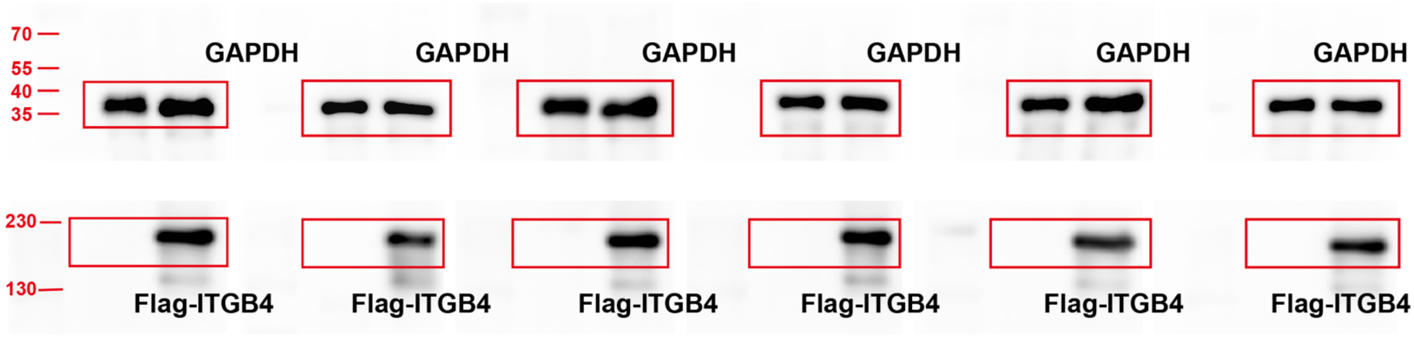

Figure S11B

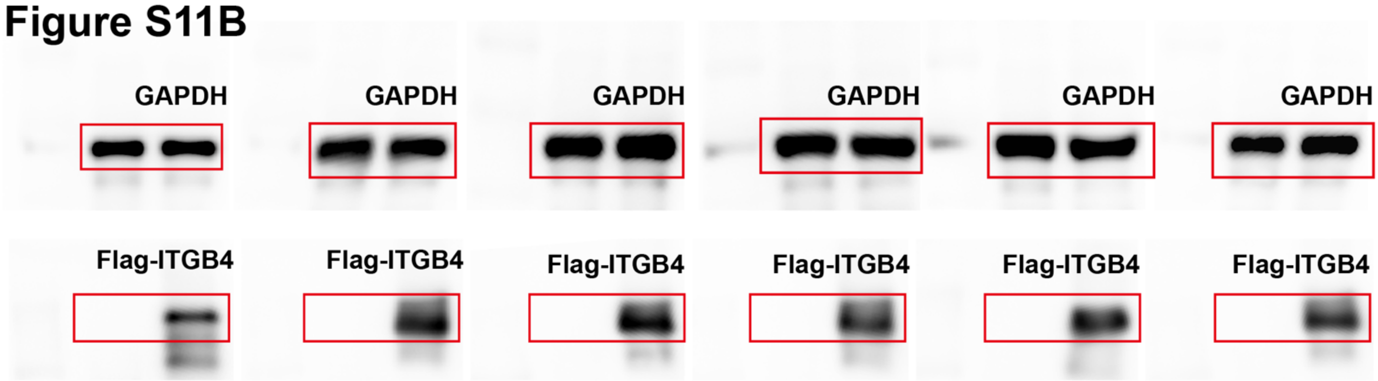

Figure 4H

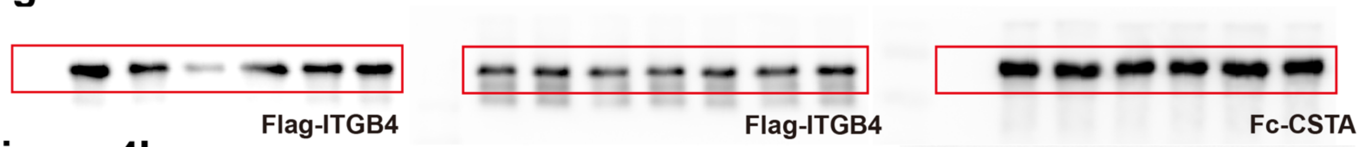

Figure 4I

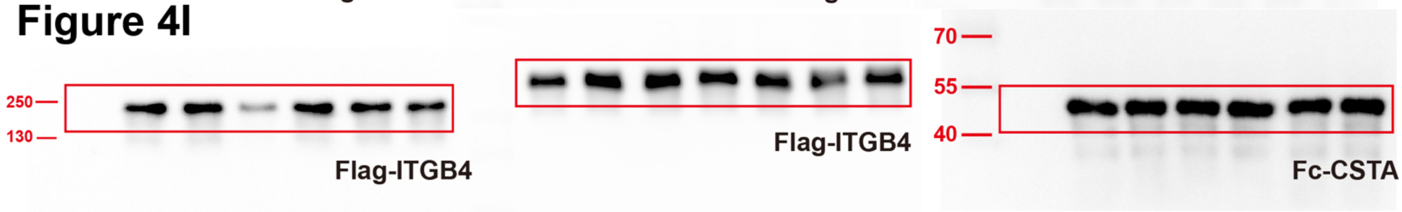

Figure S11F

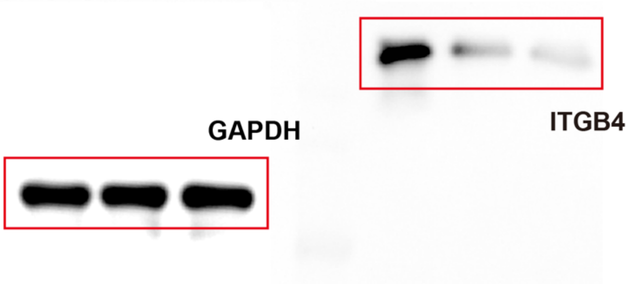

Figure S11G

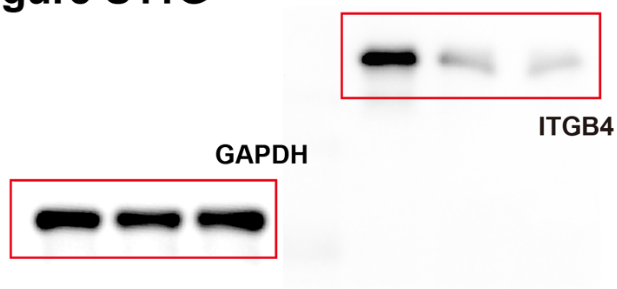

Figure 4J

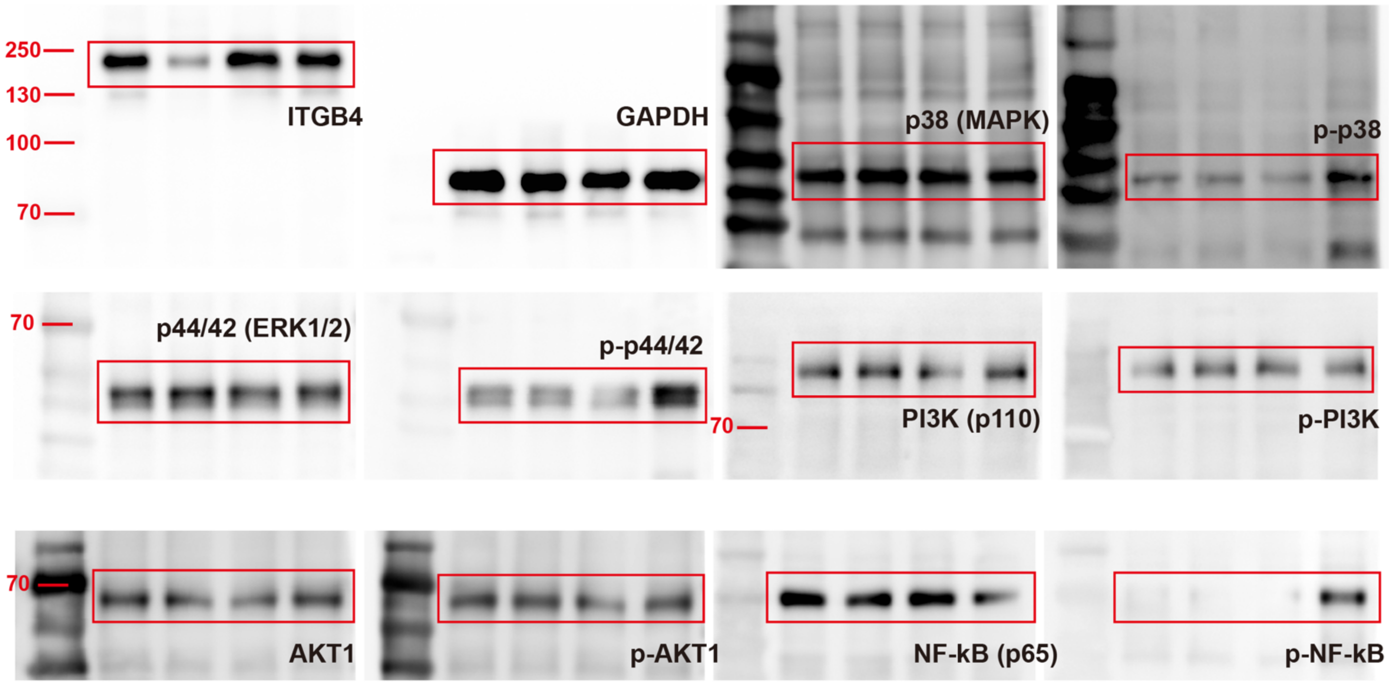

Figure 4J

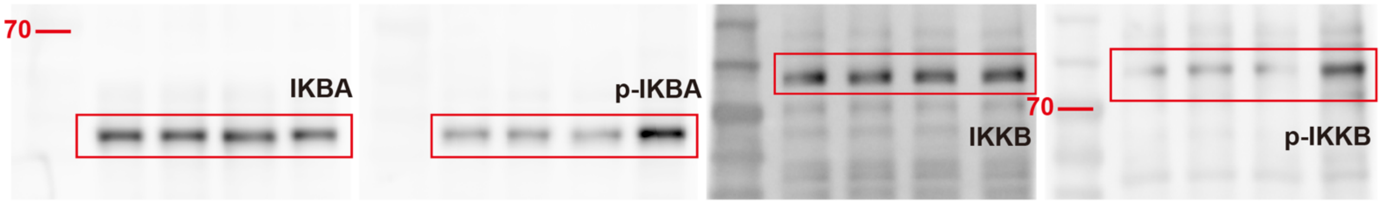

Figure S11H

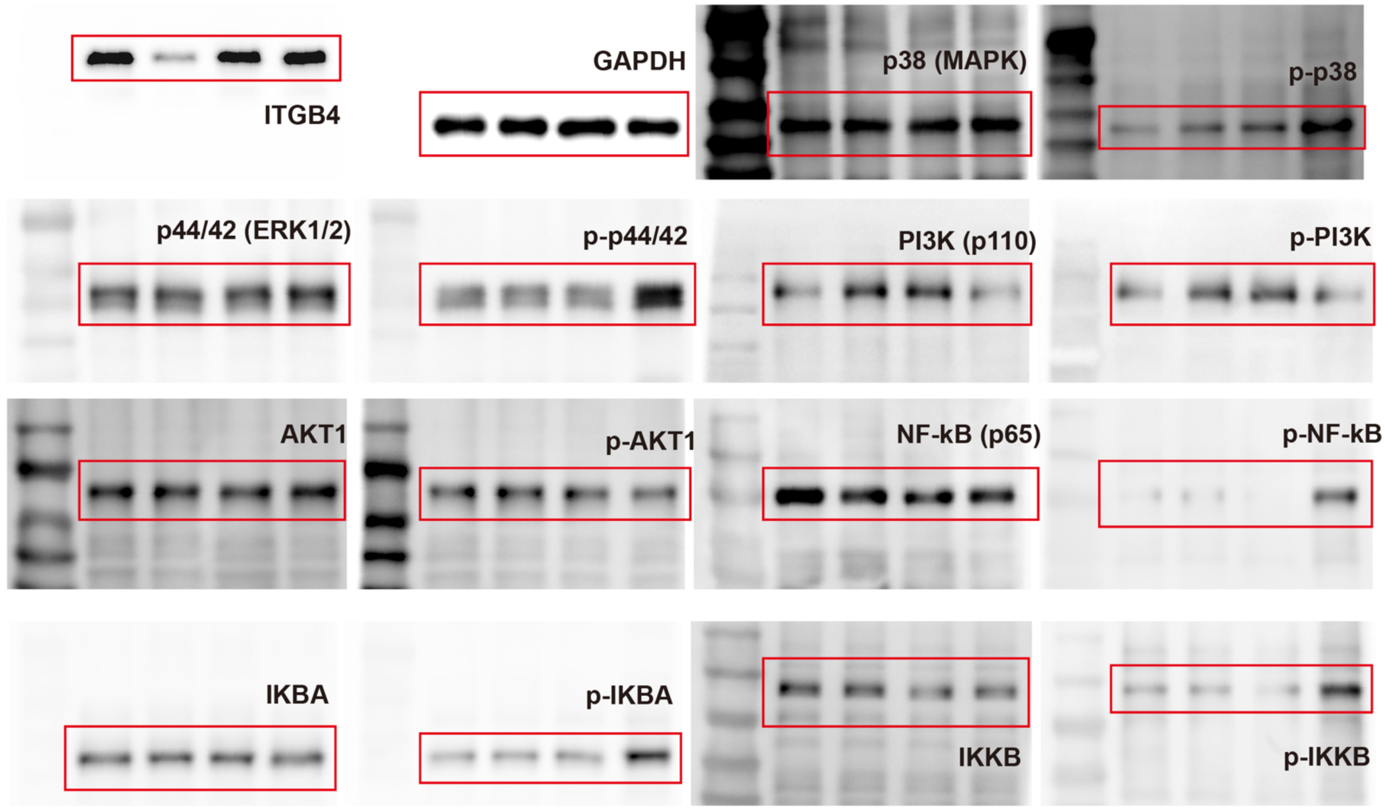

Figure 5F

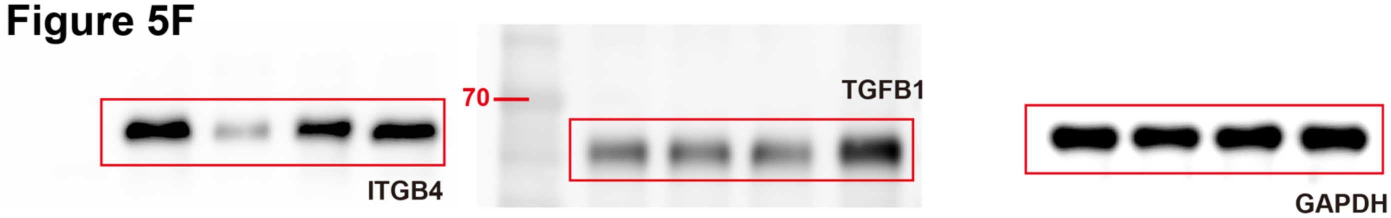

Figure S15A

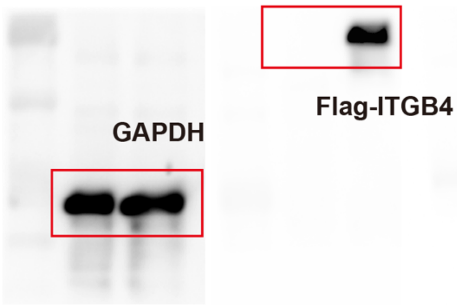

Figure S15H

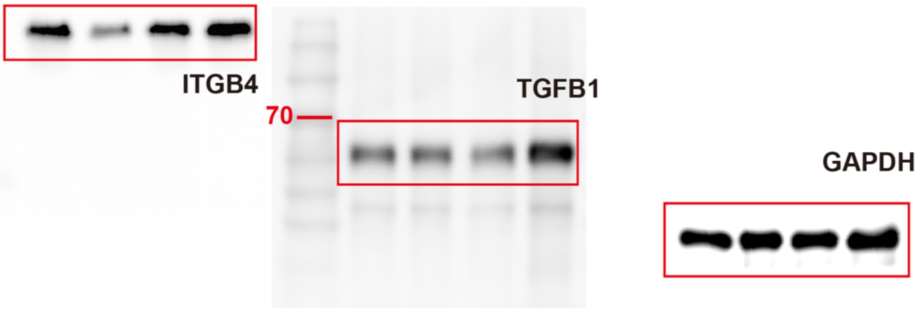

New added WB:

Figure S3C

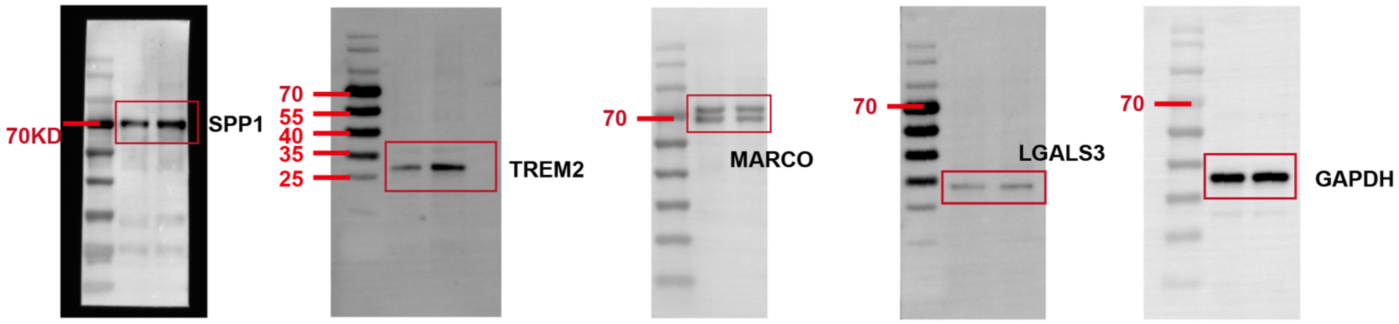

Figure S3D

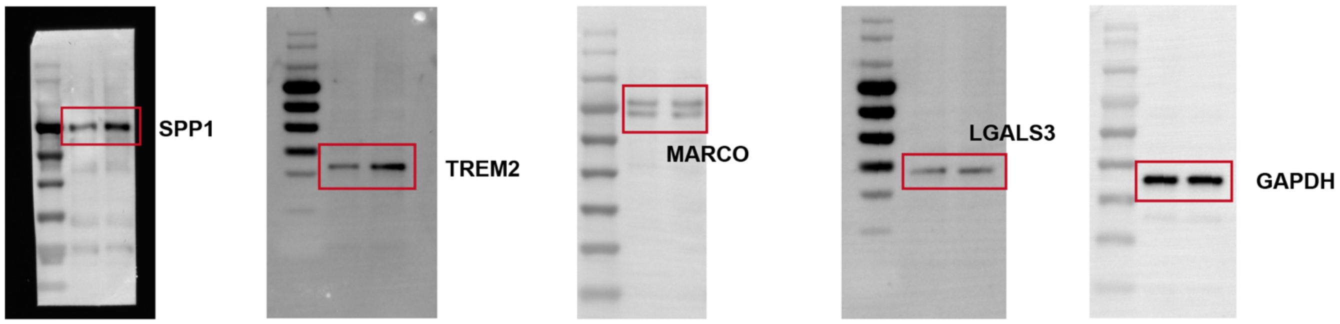

Figure 4E

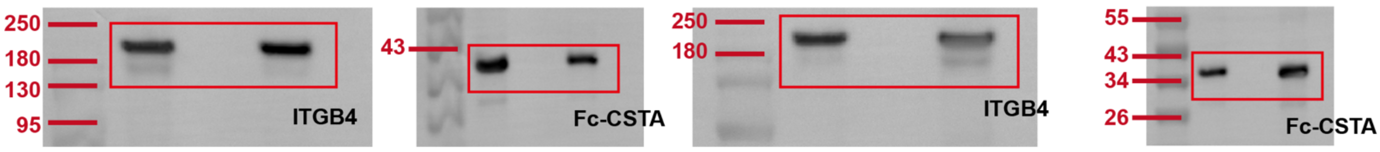

Figure 4G

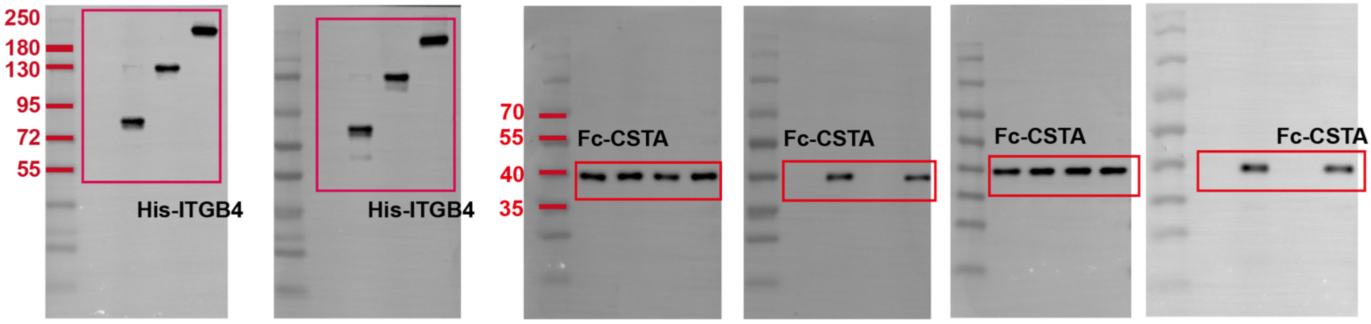

Figure S11K

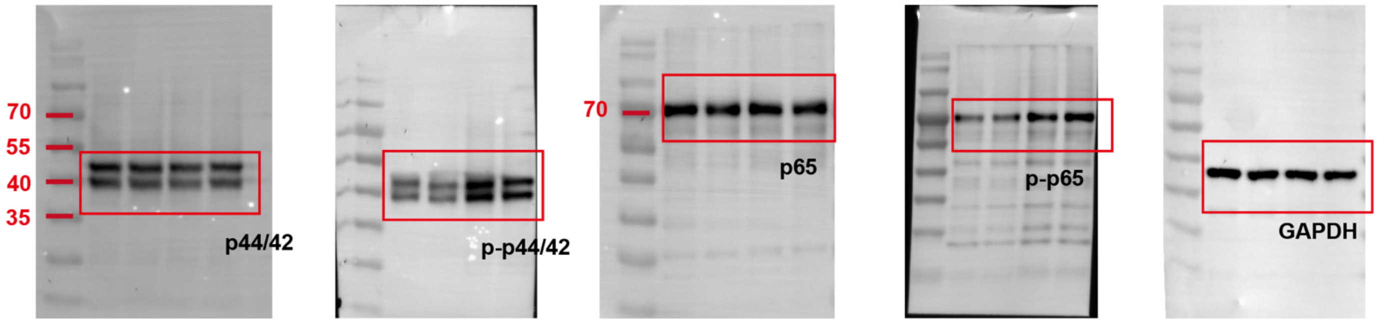

New added WB:

Figure S11L

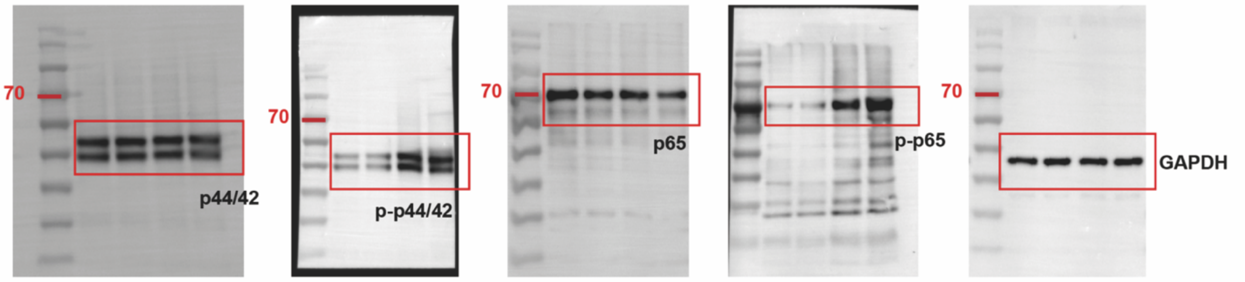

Figure 5M

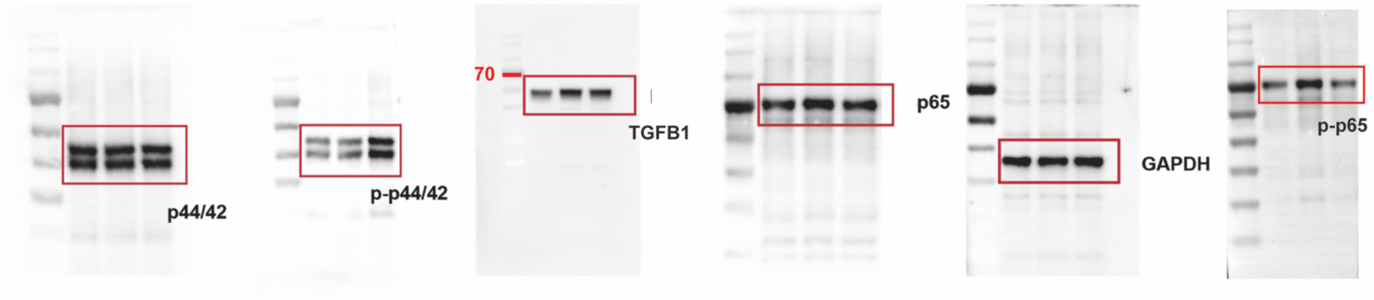

Figure S15I

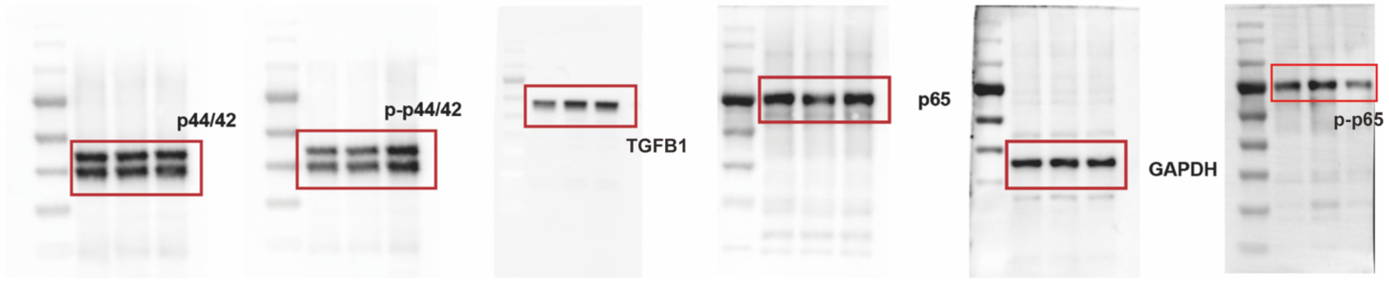

Supplement: Supplementary file 25 — Supplementary Material 25 [file 12967_2026_8009_MOESM25_ESM.pdf]
