# Supplementary material for: M2-like GAMs secreting CSTA drive glioblastoma progression via the ITGB4-TGFB1 feedback axis
Source: J Transl Med. 2026 Mar 14;24:567. doi: 10.1186/s12967-026-08009-0 (PMC13101109; doi:10.1186/s12967-026-08009-0)
Supplement: Supplementary file 26 — Supplementary Material 26 [file 12967_2026_8009_MOESM26_ESM.docx]

**Figure S1:**

**A.** The Venn plot presented the intersection of immune signatures calculated by ssGSEA with significant prognostic implications between the TCGA and CGGA cohorts. Immune signatures with hazard ratios greater than 1 or less than 1 simultaneously between these two cohorts were finally retained.

**B.** The relationship between penalty coefficient (log(λ)) values and partial likelihood deviance. The point with the lowest partial likelihood deviance corresponds to the best log(λ) value.

**C.** Relationship between the weight coefficients (Coef) corresponding to the immune signatures in the least absolute shrinkage and selection operator (LASSO) regression and log(λ) value.

**D, E.** Differences in principal components between high and low ImmSig subgroups of the TCGA **(D)** and CGGA cohort **(E)**.

**F, G.** ROC curves for ImmSig of overall survival (OS) in the TCGA **(F)** and CGGA **(G)** cohorts.

**H.** The volcano plot displayed the distribution of differentially expressed genes (DEGs) between high and low ImmSig subgroups of the TCGA cohort. DEGs with FDR <0.05 and |logFC|>1 were retained.

**I.** The correlation between systematic error and the branch number of the random forest.

**J.** The top 30 differentially expressed genes (DEGs) with the highest Mean Decrease. DEGs with Mean Decrease > 2 were selected as the input layer of the neural network.

**K.** The forest plot presented the results of the univariate Cox analysis of input layer DEGs with OS in LGG patients of the TCGA cohort.

**L, M.** The ROC curves and the corresponding AUC values reflected the accuracy of the neural network as a classifier for identifying the high and low ImmSig subgroups in the TCGA **(L)** and CGGA **(M)** cohorts, with corresponding AUC values of 0.968 (95%CI: 0.950-0.982) and 0.927 (95%CI: 0.906-0.948).

**Figure S2:**

**A.** UMAP showing spatial distribution of 26 cell subsets after dimensionality reduction clustering of GSE182109 at resolution = 0.8.

**B.** InferCNV-predicted copy number variation counts across single-cell subsets.

**C.** Intersection of subset-specific DEGs with 16 neural network immune signature genes, retaining 14 immune signatures.

**D, E, F, G.** K-M curves for prognostic relevance of CSTA in CGGA-LGG **(D)**, TCGA-LGG **(E)**, CGGA-GBM **(F)**, and TCGA-GBM **(G)** cohorts.

**H, I, J.** Differences in protein levels of CSTA, CD206, and ARG1 in RAW264.7 **(H)**, HMC3 **(I)**, and THP1 **(J)** cells with or without IL-4 treatment.

**K, L, M, N.** Construction of CSTA-knockdown cell lines in BV2 **(K)**, THP1 **(L)**, RAW264.7 **(M)**, and HMC3 **(N)**, corresponding sh.2, sh.2, sh.2, and sh.1 were selected as representative sh-CSTA lines based on knockdown efficiency.

*p<0.05, **p<0.01, ***p<0.001, ****p<0.0001.

**Figure S3:**

**A, B.** Differences in the RNA levels of GBM-promoting markers SPP1, TREM2, MARCO and LGALS3 in BV2 **(A)** and HMC3 **(B)** cells before and after IL-4 induction.

**C, D.** Differences in the protein levels of GBM-promoting markers SPP1, TREM2, MARCO and LGALS3 in BV2 (C) and HMC3 (D) cells before and after IL-4 induction.

**E.** IF co-localization staining results of CSTA with TREM2 and SPP1.

**F, G.** Correlation analysis of fluorescence intensity of CSTA with TREM2 **(F)** and SPP1 **(G)** in co-localized cells.

**H, I, J.** WB results showing the effect of CSTA knockdown on protein levels of M2-like polarization markers ARG1 and CD206 in HMC3 **(H)**, RAW264.7 **(I)**, and THP1 cells **(J)**.

*p<0.05, **p<0.01, ***p<0.001, ****p<0.0001.

**Figure S4:**

**A.** CCK-8 assay showing the correlation of GL261, U251, and LN229 cells proliferation rate with CSTA stimulation concentration and time.

**B, C, D.** Colony formation assay displaying the effect on CT2A proliferation in BV2-CT2A co-culture system under CSTA recombinant protein stimulation alone **(B)**, CSTA intervention after BV2 M2-like polarization **(C)**, and exogenous CSTA supplementation after BV2 M2-like polarization plus CSTA intervention **(D)**.

**E, F, G.** Scratch assay displaying the effect on CT2A migration in BV2-CT2A co-culture system under CSTA recombinant protein stimulation alone **(E)**, CSTA intervention after BV2 M2-like polarization **(F)**, and exogenous CSTA supplementation after BV2 M2-like polarization plus CSTA intervention **(G)**.

**H, I, J.** Transwell assay displaying the effect on CT2A invasion in BV2-CT2A co-culture system under CSTA recombinant protein stimulation alone **(H)**, CSTA intervention after BV2 M2-like polarization **(I)**, and exogenous CSTA supplementation after BV2 M2-like polarization plus CSTA intervention **(J)**.

*p<0.05, **p<0.01, ***p<0.001, ****p<0.0001.

**Figure S5:**

**A, B, C.** EdU assay displaying the effect on GL261 proliferation in BV2-GL261 co-culture system under CSTA recombinant protein stimulation alone **(A)**, CSTA intervention after BV2 M2-like polarization **(B)**, and exogenous CSTA supplementation after BV2 M2-like polarization plus CSTA intervention **(C)**.

**D, E, F.** Colony formation assay displaying the effect on GL261 proliferation in BV2-GL261 co-culture system under CSTA recombinant protein stimulation alone **(D)**, CSTA intervention after BV2 M2-like polarization **(E)**, and exogenous CSTA supplementation after BV2 M2-like polarization plus CSTA intervention **(F)**.

**G, H, I.** Scratch assay displaying the effect on GL261 migration in BV2-GL261 co-culture system under CSTA recombinant protein stimulation alone **(G)**, CSTA intervention after BV2 M2-like polarization **(H)**, and exogenous CSTA supplementation after BV2 M2-like polarization plus CSTA intervention **(I)**.

**J, K, L.** Transwell assay displaying the effect on GL261 invasion in BV2-GL261 co-culture system under CSTA recombinant protein stimulation alone **(J)**, CSTA intervention after BV2 M2-like polarization **(K)**, and exogenous CSTA supplementation after BV2 M2-like polarization plus CSTA intervention **(L)**. *p<0.05, **p<0.01, ***p<0.001, ****p<0.0001.

**Figure S6:**

**A, B, C.** EdU assay displaying the effect on LN229 proliferation in HMC3-LN229 co-culture system under CSTA recombinant protein stimulation alone **(A)**, CSTA intervention after HMC3 M2-like polarization **(B)**, and exogenous CSTA supplementation after HMC3 M2-like polarization plus CSTA intervention **(C)**.

**D, E, F.** Colony formation assay displaying the effect on LN229 proliferation in HMC3-LN229 co-culture system under CSTA recombinant protein stimulation alone **(D)**, CSTA intervention after HMC3 M2-like polarization **(E)**, and exogenous CSTA supplementation after HMC3 M2-like polarization plus CSTA intervention **(F)**.

**G, H, I.** Scratch assay displaying the effect on LN229 migration in HMC3-LN229 co-culture system under CSTA recombinant protein stimulation alone **(G)**, CSTA intervention after HMC3 M2-like polarization **(H)**, and exogenous CSTA supplementation after HMC3 M2-like polarization plus CSTA intervention **(I)**.

**J, K, L.** Transwell assay displaying the effect on LN229 invasion in HMC3-LN229 co-culture system under CSTA recombinant protein stimulation alone **(J)**, CSTA intervention after HMC3 M2-like polarization **(K)**, and exogenous CSTA supplementation after HMC3 M2-like polarization plus CSTA intervention **(L)**. *p<0.05, **p<0.01, ***p<0.001, ****p<0.0001.

**Figure S7:**

**A, B, C.** EdU assay displaying the effect on U251 proliferation in HMC3-U251 co-culture system under CSTA recombinant protein stimulation alone **(A)**, CSTA intervention after HMC3 M2-like polarization **(B)**, and exogenous CSTA supplementation after HMC3 M2-like polarization plus CSTA intervention **(C)**.

**D, E, F.** Colony formation assay displaying the effect on U251 proliferation in HMC3-U251 co-culture system under CSTA recombinant protein stimulation alone **(D)**, CSTA intervention after HMC3 M2-like polarization **(E)**, and exogenous CSTA supplementation after HMC3 M2-like polarization plus CSTA intervention **(F)**.

**G, H, I.** Scratch assay displaying the effect on U251 migration in HMC3-U251 co-culture system under CSTA recombinant protein stimulation alone **(G)**, CSTA intervention after HMC3 M2-like polarization **(H)**, and exogenous CSTA supplementation after HMC3 M2-like polarization plus CSTA intervention **(I)**.

**J, K, L.** Transwell assay displaying the effect on U251 invasion in HMC3-U251 co-culture system under CSTA recombinant protein stimulation alone **(J)**, CSTA intervention after HMC3 M2-like polarization **(K)**, and exogenous CSTA supplementation after HMC3 M2-like polarization plus CSTA intervention **(L)**. *p<0.05, **p<0.01, ***p<0.001, ****p<0.0001.

**Figure S8:**

**A** In the survival analysis cohorts of CT2A- and GL261-BV2 mixed tumor formation models, significant fluorescent readings detected by in vivo imaging on day 7 confirmed successful model establishment.

**B, C.** In vivo imaging **(B)** and fluorescence readout statistics **(C)** at 7, 14 and 21 days in time-point analysis cohort in the GL261-BV2 mixed orthotopic GBM xenograft models.

**D, E.** HE staining of mouse brain coronal sections **(D)** and maximum cross-sectional area statistics **(E)** at 21 days in time-point analysis cohort.

**F.** K-M curve showing survival time and status of different subgroups in survival analysis cohort of the GL261-BV2 mixed tumor formation model.

*p<0.05, **p<0.01, ***p<0.001, ****p<0.0001.

**Figure S9:**

**A, B.** WB validation of c-FOS knockdown in BV2 **(A)** and HMC3 **(B)** cells.

**C, D.** Quantification of WB results for c-FOS knockdown in BV2 **(C)** and HMC3 **(D)** cells, with both sh.2 (highest efficiency) designated as representative sh-FOS for each cell line. **E.** Effect of c-FOS knockdown on CSTA protein levels in IL-4-induced HMC3 cells.

**F, G.** WB validation of RAP1A overexpression in BV2 **(F)** and HMC3 **(G)** cells, with both OE.2 (highest efficiency) designated as representative OE-RAP1A for each cell line.

**H.** Changes in protein levels of CSTA, c-JUN, c-FOS, and phosphorylated signaling molecules in HMC3 cells after separate activation of MAPK, JAK/STAT, and RAP1 signaling pathways.

**I, J.** Changes in protein levels of CSTA, c-JUN, c-FOS, and phosphorylated signaling molecules in HMC3 cells after simultaneous MAPK inhibition and IL-4 induction **(I)**, as well as after simultaneous sh-FOS and MAPK activation **(J)**.

*p<0.05, **p<0.01, ***p<0.001, ****p<0.0001.

**Figure S10:**

Top 5 plasma membrane proteins from affinity IP-MS results ranked by RRA: PTPRG, PTPRK, AAK1, SLC7A1, ITGB4. Prognostic relevance tested in TCGA, CGGA, CPTAC, and Gravendeel cohorts; ITGB4 emerged as the most robust, serving as a significant prognostic risk factor across all four cohorts.

**Figure S11:**

**A.** Molecular docking prediction of human CSTA and ITGB4, enlarged area shows potential binding sites and corresponding amino acid residues.

**B, C.** Validation of ITGA6 knockdown efficiency in CT2A **(B)** and LN229 **(C)** cells, sh.1 in CT2A and sh.2 in LN229 were finally selected.

**D, E.** Construction of Flag-tagged ITGB4 with 87–91 amino acid point mutations (AAPM) in BV2 **(D)** and LN229 **(E)** cells.

**F, G.** Construction of ITGB4-knockdown CT2A **(F)** and LN229 **(G)** cells, with both sh.2 (best knockdown efficiency) designated as sh-ITGB4.

**H.** Phosphorylation levels of signaling molecules in ITGB4-related pathways (MAPK, PI3K/AKT, NF-κB) in CT2A cells after CSTA stimulation, and effects of AAPM #88 and sh-ITGB4.

**I, J.** Validation of ITGB1 knockdown efficiency in LN229 **(I)** and CT2A **(J)** cells, sh.2 in CT2A and sh.1 in LN229 were finally selected.

**K, L.** Effects of shITGA6 and shITB1 on the phosphorylation levels of key signaling molecules in the MAPK and NF-κB pathways in CT2A **(K)** and LN229 **(L)** cells after CSTA induction

*p<0.05, **p<0.01, ***p<0.0 01, ****p<0.0001

**Figure S12:**

**A, B, C.** EdU **(A)**, scratch **(B)**, and Transwell **(C)** assays assessing the impact of MAPK or NF-κB inhibition on CSTA-induced CT2A cell proliferation, invasion, and migration.

**D, E, F.** Statistical analyses of EdU **(D)**, scratch **(E)**, and Transwell **(F)** results in CT2A cells.

**G, H, I.** EdU **(G)**, scratch **(H)**, and Transwell **(I)** assays assessing the impact of MAPK or NF-κB inhibition on CSTA-induced LN229 cell proliferation, invasion, and migration.

**J, K, L.** Statistical analyses of EdU **(J)**, scratch **(K)**, and Transwell **(L)** results in LN229 cells.

*p<0.05, **p<0.01, ***p<0.0 01, ****p<0.0001

**Figure S13:**

**A, B, C.** EdU **(A)**, scratch **(B)**, and Transwell **(C)** assays assessing the impact of shITGA6 or shITGB1 on CSTA-induced CT2A cell proliferation, invasion, and migration.

**D, E, F.** Statistical analyses of EdU **(D)**, scratch **(E)**, and Transwell **(F)** results in CT2A cells.

**G, H, I.** EdU **(G)**, scratch **(H)**, and Transwell **(I)** assays assessing the impact of shITGA6 or shITGB1 on CSTA-induced LN229 cell proliferation, invasion, and migration.

**J, K, L.** Statistical analyses of EdU **(J)**, scratch **(K)**, and Transwell **(L)** results in LN229 cells.

*p<0.05, **p<0.01, ***p<0.0 01, ****p<0.0001

**Figure S14:**

**A, B, C.** EdU **(A)**, scratch **(B)**, and Transwell **(C)** assays assessing the impact of ITGB4 knockdown or AAPM #88 on CSTA-induced CT2A cell proliferation, invasion, and migration.

**D, E, F.** Statistical analyses of EdU **(D)**, scratch **(E)**, and Transwell **(F)** results in CT2A cells.

**G, H, I.** EdU **(G)**, scratch **(H)**, and Transwell **(I)** assays assessing the impact of ITGB4 knockdown or AAPM #88 on CSTA-induced LN229 cell proliferation, invasion, and migration.

**J, K, L.** Statistical analyses of EdU **(J)**, scratch **(K)**, and Transwell **(L)** results in LN229 cells.

*p<0.05, **p<0.01, ***p<0.0 01, ****p<0.0001

**Figure S15:**

**A.** Construction of Flag-tagged ITGB4 AAPM #88 cell line in GL261 cells.

**B.** In the survival analysis cohorts of CT2A- and GL261-BV2 mixed orthotopic GBM xenograft models, significant fluorescent readings detected by in vivo imaging on day 7 confirmed successful model establishment.

**C, D.** In vivo imaging **(C)** and fluorescence readout statistics **(D)** at 7, 14 and 21 days in time-point analysis cohort in the GL261-BV2 mixed tumor formation model.

**E, F.** HE staining of mouse brain coronal sections **(E)** and maximum cross-sectional area statistics **(F)** at 21 days in time-point analysis cohort.

**G.** K-M curves showing survival time and status of different subgroups in survival analysis cohort of the GL261-BV2 mixed orthotopic GBM xenograft models.

**H.** Protein levels of TGFB1 in LN229 cells after CSTA stimulation, and effects of AAPM #88 and sh-ITGB4.

**I.** Differences in the phosphorylation of key proteins of the relevant signaling pathways and TGFB1 protein levels in LN229 cells following treatment with MAPK and NF-κB signaling pathway agonists

*p<0.05, **p<0.01, ***p<0.001, ****p<0.0001.

**Figure S16:**

**A, B, C.** IF staining of TGFB1 and macrophage/microglia marker IBA1 in brain sections from GL261-BV2 orthotopic GBM xenograft models **(A)**, with statistics on the proportions of TGFB1+ **(B)** and IBA1+ **(C)** cells among total cells across groups.

**D, E.** IF staining of IBA1 and ARG1 **(D)**, and statistics on the proportion of IBA1+ARG1+ cells relative to total cells in the visual field across groups **(E)**.

**F.** Correlation between TGFB1+ and IBA1+ARG1+ cell proportions, with each point representing values from an entire field.

**G.** CSTA concentrations in serum samples from patients with different glioma grades.

**H.** CSTA concentrations in preoperative serum samples from primary vs. recurrent glioma patients.

**I, J.** ROC analysis of serum CSTA concentrations in glioma vs. normal patients **(I)** and in LGG vs. GBM patients **(J)**, with corresponding AUC values of 0.9873 and 0.9653.

**K.** Paired t-test of serum CSTA concentrations in patients with different glioma grades before and 1 week after surgery.

*p<0.05, **p<0.01, ***p<0.001, ****p<0.0001.
